# Effects of Nut Consumption on Body Composition: A Systematic Review and Meta-Analysis of Randomized Controlled Trials with Subgroup Analyses by Nut Fatty-Acid Profile

**DOI:** 10.3390/foods15203594

**Published:** 2026-10-10

**Authors:** Weili Hu, Meihan Chen, Zixi Wang, Sunhye Shin, Jung Eun Kim

**Affiliations:** 1 Department of Food Science and Technology, National University of Singapore, 2 Science Drive 2, Block S14, Level 6, Singapore 117542, Singapore; wendy.weili.hu@nus.edu.sg (W.H.);; 2 Department of Food and Nutrition, Seoul Women’s University, 621 Hwarang-ro, Nowon-gu, Seoul 01797, Republic of Korea; 3 Bezos Center for Sustainable Protein, National University of Singapore, Singapore 117643, Singapore

**Keywords:** nuts, monounsaturated fatty acids, polyunsaturated fatty acids body composition, lipid profile

## Abstract

Background: Despite their high fat content, nut consumption is suggested to benefit body composition. However, it remains unclear whether nuts with different fatty-acid compositions affect body composition differently. This study aimed to assess the effects of monounsaturated fatty acid (MUFA)-rich and polyunsaturated fatty acid (PUFA)-rich nut consumption on body composition. Methods: A systematic search of PubMed, Embase, CINAHL, Cochrane, and additional sources identified 3118 articles, with 154 meeting the inclusion criteria. Subgroup analyses were conducted by nut fatty-acid composition and control diet type. Nuts were classified as MUFA-rich (MUFA:PUFA ratio ≥ 1.3) or PUFA-rich (<1.3) based on the percentages of total fat content. Results: Although there were no effects on body mass, BMI, or waist circumference, nut consumption significantly reduced % fat mass (WMD: −0.28%, 95% CI: −0.53 to −0.02) and fat mass (WMD: −0.41 kg, 95% CI: −0.58 to −0.27) and increased % lean mass (WMD: 0.71%, 95% CI: 0.32 to 1.10). However, absolute lean mass did not change significantly. Benefits were observed only in the MUFA-rich subgroup, although subgroup differences were not significant. Both MUFA- and PUFA-rich nut consumption showed reduced total cholesterol and low-density lipoprotein cholesterol levels, while MUFA-rich nut consumption further reduced triglyceride levels. Conclusion: Nut consumption, particularly MUFA-rich nuts, can improve body composition by percentage fat mass and absolute fat mass. Consuming either MUFA-rich and PUFA-rich nuts can further improve lipid–lipoprotein profiles.

## 1. Introduction

Fat accumulation is a fundamental aspect of body composition and its excessive accumulation has been associated with an increased risk of obesity, cardiovascular diseases, and diabetes mellitus [1,2,3]. Conversely, enhanced body composition by reduction in fat mass percentage and increase in lean mass percentage has been associated with improved insulin sensitivity and secretion, lower blood pressure, and reduced all-cause mortality [4,5,6]. Therefore, maintaining an optimal body composition—characterized by lower fat mass and higher lean mass—is essential for mitigating the risks associated with obesity and metabolic disorders, ultimately contributing to improved cardiovascular health.

Dietary interventions are a key strategy for managing body composition and specifically, consumption of nuts has been associated with beneficial effects on body composition [7]. Nuts, including both tree nuts and peanuts, are recognized as nutrient-dense foods and they are particularly rich in unsaturated fatty acids as well as proteins, fibers, vitamins, minerals, and phytochemicals [8]. Although nuts have a high fat content, their consumption does not generally appear to increase the risk of obesity; on the contrary, it may reduce the risk of obesity, cardiometabolic diseases, and diabetes mellitus [9].

One potential mechanism underlying these beneficial effects is increasing the proportion of unsaturated fatty acids in the diet by including nuts. In particular, polyunsaturated fatty acids (PUFA) can enhance fat oxidation by activating peroxisome proliferator-activated receptors (PPAR) and promote satiety as well as decrease energy intake by stimulating the secretion of peptide YY (PYY). This combination contributes to weight loss and improvements in body composition by supporting reductions in fat mass [10,11]. Research also suggests that particularly long-chain n-3 PUFA can enhance muscle protein synthesis and could be linked to improved body composition, as an 8-week supplementation with this PUFA significantly increased muscle protein synthesis rates in healthy young and middle-aged adults [12]. Additionally, higher dietary intake of PUFA has been associated with a reduced risk of low muscle mass, suggesting their role in preserving lean body mass [13]. Other researchers also suggested that it could be due to the consumption of monounsaturated fatty acids (MUFA) from nuts; a MUFA-rich meal acutely results in a lower respiratory exchange ratio and greater diet-induced thermogenesis (DIT), indicating enhanced energy expenditure and preferential utilization of fat for energy compared to a PUFA-rich meal [14,15].

Given the inconsistencies in the reported comparative effectiveness of MUFA and PUFA intake on body composition and weight management, the present study examines whether the effects of nut consumption differ according to the fatty-acid composition of the nuts. Specifically, the present study aimed to systematically assess the effects of nut consumption on body composition through a meta-analysis of relevant randomized controlled trials. Subgroup analyses were conducted to compare MUFA-rich and PUFA-rich nuts. Additionally, meta-regression was performed to examine the association between MUFA intake and changes in body composition.

## 2. Materials and Methods

### 2.1. Search Strategy

The participant, intervention, comparison, outcome, and study design (PICOS) statement is shown in Table 1. The systematic review of the published literature was conducted according to the Preferred Reporting Items for Systematic Reviews and Meta-Analyses (PRISMA) guidelines and was registered in PROSPERO (no. CRD42023480512) [16].

### 2.2. Study Selection, Data Extraction, and Quality Assessment

Independent screening of titles and abstracts was conducted by two independent reviewers based on the following exclusion criteria: (1) non-randomized controlled trial (RCT) study design; (2) study populations involving children aged under 18 years, pregnant or breastfeeding women, animals, or *in vitro* studies; (3) primary outcome not reported; (4) interventions involving physical activity, as it may confound the effects of the intervention; (5) interventions utilizing processed nut products (e.g., nut butter or nut oils); (6) articles in which nuts were combined with other foods, where the observed effects could not be attributed solely to nuts; and (7) mixed nut diets where the composition of the nuts was not specified. Any discrepancies during the screening and extraction process were discussed and resolved through consensus between the two reviewers, with a third reviewer consulted when necessary.

During the review process, data were independently extracted by the two reviewers. The extracted data included: author, the year of publication, study intervals, number of subjects in each study group, study duration, participant age, type of nut used in the intervention, intervention dosage, as well as pre- and post-intervention mean values and standard deviations for both primary and secondary outcomes. The outcomes assessed included body mass, body mass index (BMI), waist circumference (WC), % fat mass, fat mass, % lean mass, lean mass, fasting blood glucose (FBG), lipid profile, energy intake, resting energy expenditure (REE), and appetite-related parameters and hormones. Secondary outcomes included triglycerides (TG), high-density lipoprotein cholesterol (HDL-C), low-density lipoprotein cholesterol (LDL-C), and total cholesterol (TC). Corresponding authors of the selected articles were contacted when further information was required.

The risk-of-bias tool for randomized trials version 2 (RoB 2), as outlined in the Cochrane Handbook for Systematic Reviews of Interventions, was employed to assess the risk of bias in each included study. Articles were categorized as having low risk, some concerns, or high risk of bias across the following domains: the randomization process, deviations from intended interventions, missing outcome data, measurement of the outcome, selection of the reported result, and overall bias [17]. The risk of bias was independently evaluated by two researchers, with any discrepancies resolved through discussion with the other co-authors.

### 2.3. Data Synthesis and Analysis

Extracted data were reported as means with standard deviations or as weighted mean differences (WMDs) and 95% confidence intervals (CIs). Meta-analyses were conducted for the primary outcomes, which included body mass, BMI, WC, % fat mass, fat mass, % lean mass, lean mass, and energy intake, as well as for the secondary outcomes. Subgroup analyses were performed based on the type of nuts (classified as either MUFA-rich or PUFA-rich) and the diet employed in the control group, where applicable. The MUFA/PUFA content for the nuts was calculated using the USDA database and extracted from the study by George et al. [18]. Nuts were classified as MUFA-rich or PUFA-rich according to their MUFA ratio. The fatty-acid composition of each tree nut was obtained from the USDA database. Saturated fatty acids (SFA), MUFA, and PUFA were expressed as percentages of total lipid, and the MUFA ratio was calculated by dividing the MUFA percentage by the PUFA percentage. For this subgroup analysis, a MUFA ratio of 1.3 was used as an operational cutoff. This cutoff was adapted from the MUFA and PUFA components of the approximate whole-diet SFA:MUFA ratio of 1:1.3:1 discussed by Hayes [19]. Accordingly, nuts with a MUFA ratio ≥ 1.3 were classified as MUFA-rich, whereas nuts with a ratio <1.3 were classified as PUFA-rich. The fatty-acid composition, calculated ratio, and classification of each nut type are presented in Supplementary Table S2. Control group diets were categorized as follows: CARB diet, in which an equal amount or caloric equivalent of carbohydrate-rich snacks, such as crackers, was provided; FAT diet, in which an equivalent amount or caloric content of fats, such as olive oil, was administered; HABITUAL/HLS diet, where the habitual diet was maintained, in some cases specifying no nut consumption; HYPOENERGETIC, in which both groups were subjected to caloric restriction; or ISOENERGETIC, in which the control group received a mixed snack that was isoenergetic in terms of macronutrient content.

The overall effect sizes were calculated using the ‘metagen’ function (R version 4.3.3), with the random-effects model to account for heterogeneity and variance among the RCTs. For crossover trials, paired intervention and control measurements from the same participants were used to calculate the mean within-participant treatment difference and its standard error, thereby accounting for the correlation between treatment periods. For parallel-group trials, baseline–follow-up correlations were used, where required, to calculate change-score standard deviations within each study arm; intervention and control groups were otherwise treated as independent. Sensitivity analyses were performed by sequentially removing high-risk articles to assess the robustness of the meta-analysis. Additionally, meta-regression was conducted using the ‘metareg’ function to evaluate the association between the amount of MUFA supplied by the additional nuts in the intervention (g/day) and relative body composition indicators. When not directly reported, this amount was calculated by multiplying the prescribed daily nut dose (g/day) by the MUFA content of the corresponding nut (g/g). All data analyses were carried out using RStudio software (R version 4.3.3), with statistical significance set at a *p*-value of <0.05.

## 3. Results

### 3.1. Literature Search

**Figure 1:**
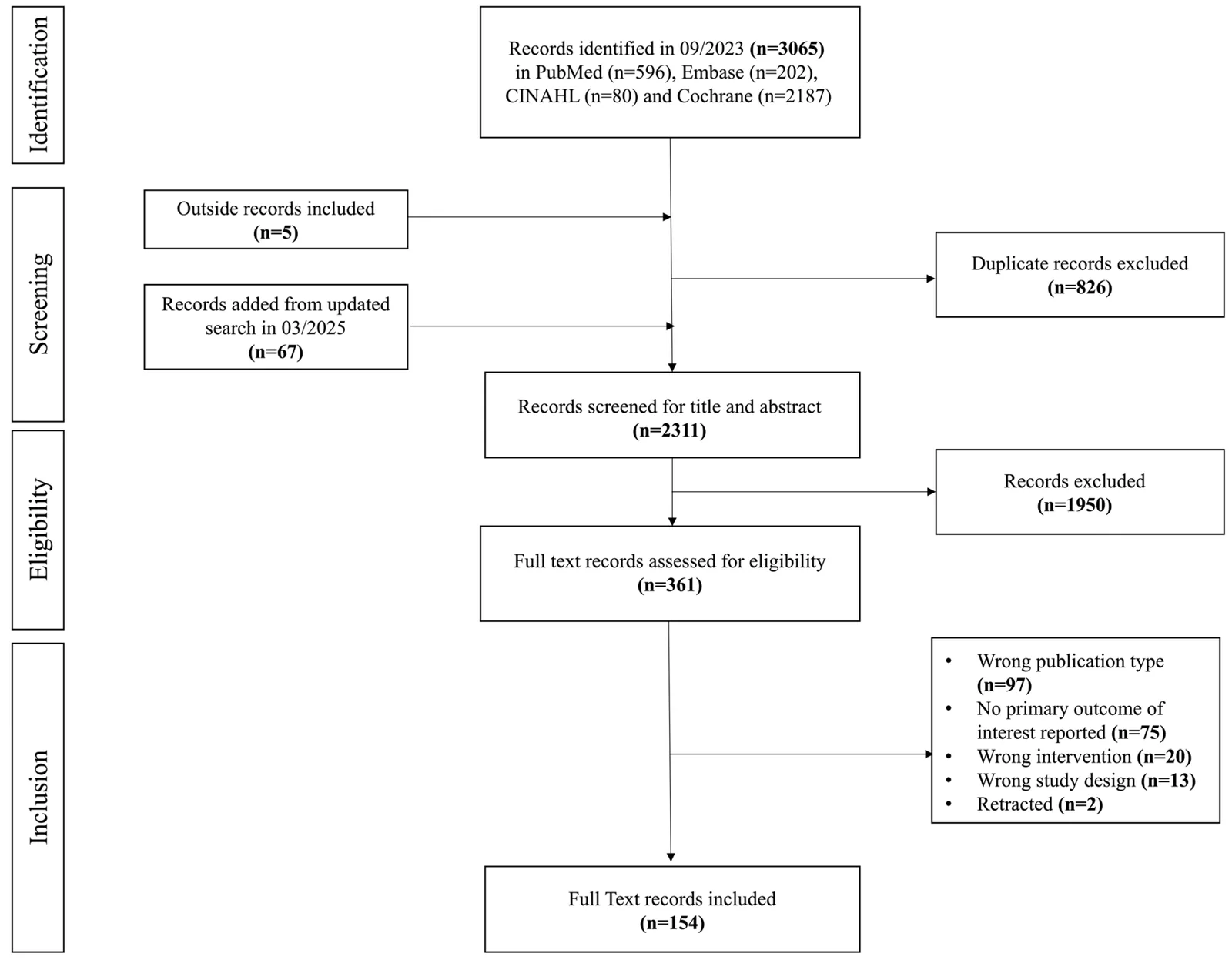
PRISMA flow diagram of the study selection process.

The study selection process is shown in Figure 1. A total of 3065 articles (PubMed *n* = 596, Embase *n* = 202, CINAHL *n* = 80 and Cochrane *n* = 2187) with an additional 5 articles obtained from external sources and 67 articles from the updated search in 2025 were identified from the search process. After the removal of duplicates (*n* = 826), 2311 articles remained for screening. Of these, 1950 articles were excluded, leaving 361 records for full-text review. Among these, 207 articles were further excluded for the following reasons: 97 were of an incorrect publication type, 75 did not report the primary outcomes of interest, 20 employed an incorrect intervention, 13 utilized an inappropriate study design, and 2 were retracted. Ultimately, 154 articles were included in this systematic review and 102 were included in the further quantitative meta-analysis.

### 3.2. Study Characteristics

**Figure 2:**
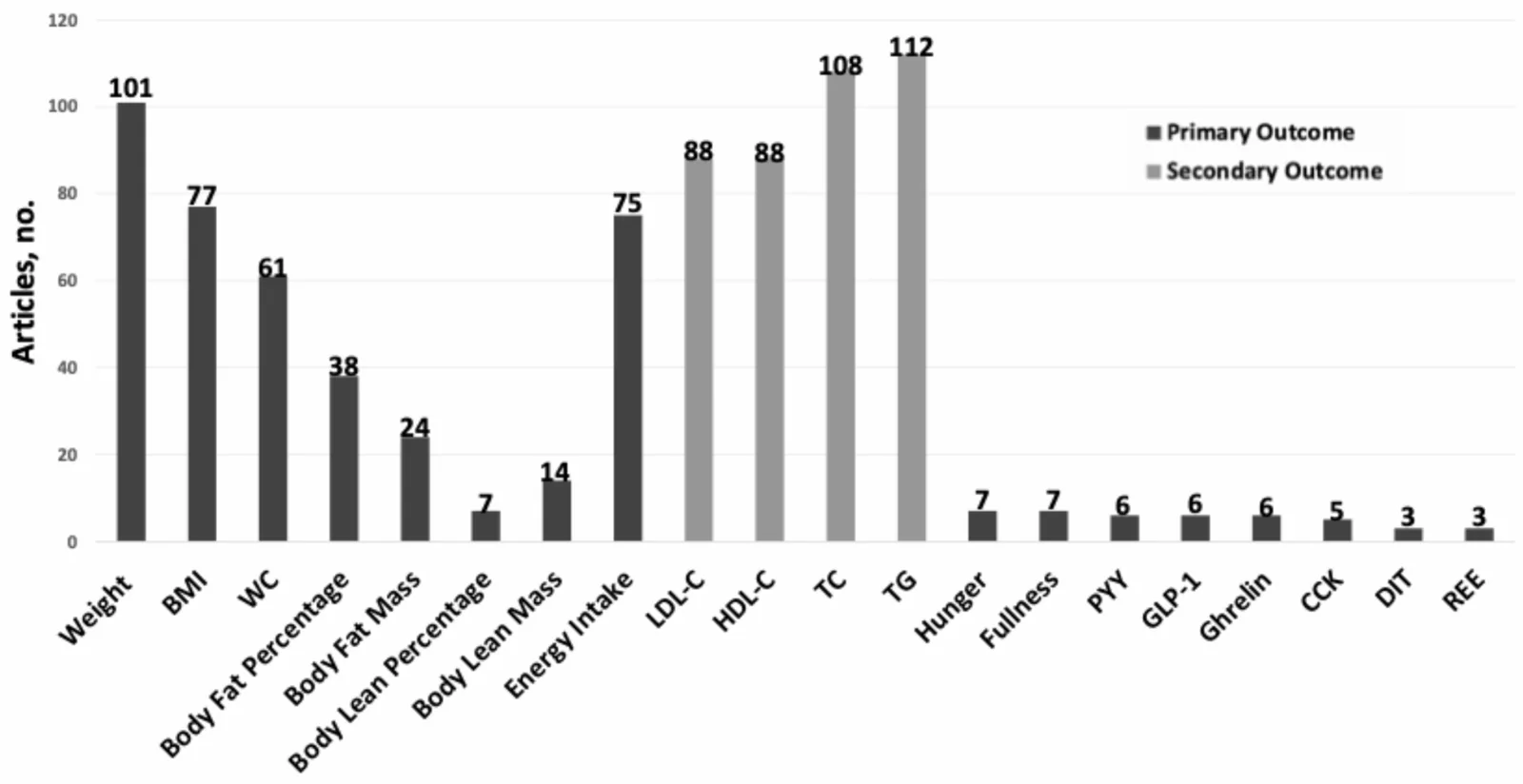
Number of articles reporting primary and secondary outcomes. CCK (cholecystokinin); DIT (diet-induced thermogenesis); energy intake (daily energy consumption); GLP-1 (glucagon-like peptide-1); HDL-C (high-density lipoprotein cholesterol); LDL-C (low-density lipoprotein cholesterol); PYY (peptide YY); REE (resting energy expenditure); TC (total cholesterol); TG (triglycerides); WC (waist circumference).

A summary of the number of articles for both primary and secondary outcomes is provided in Figure 2. A relatively sufficient number of articles were included to evaluate primary outcomes such as body weight, BMI, WC, and body composition. However, a smaller subset of articles reported on appetite and energy metabolism-related biomarkers. Additionally, a substantial number of articles also reported secondary outcomes, particularly the lipid-lipoprotein profile, indicating a broader representation in this area. Detailed major findings of the included articles and dietary interventions are summarized in Table S3 and the summary for studies comparing two different types of nuts and their effects on health outcomes are presented in Table S4.

The median follow-up duration was 21 weeks, with a range from 2 to 260 weeks. The systematic review included 154 articles representing 119 unique RCTs. Of these articles, 134 reported long-term outcomes and 24 reported acute postprandial outcomes, with 4 articles reporting both. Overall, 57 articles used crossover designs and 97 used parallel-group designs [20,21,22,23,24,25,26,27,28,29,30,31,32,33,34,35,36,37,38,39,40,41,42,43,44,45,46,47,48,49,50,51,52,53,54,55,56,57,58,59,60,61,62,63,64,65,66,67,,68,69,70,71,72,73,74,75,76,77,78,79,80,81,82,83,84,85,86,87,88,89,90,91,92,93,94,95,96,97,98,99,100,101,102,103,104,105,106,107,108,109,110,111,112,113,114,115,116,117,118,119,120,121,122,123,124,125,126,127,128,129,130,131,132,133,134,135,136,137,138,139,140,141,142,143,144,145,146,147,148,149,150,151,152,153,154,155,156,157,158,159,160,161,162,163,164,165,166,167,168,169,170,171,172]. The participant conditions varied and included overweight or obese, healthy individuals, type 2 diabetes mellitus, hypercholesterolemia, cardiovascular disease, hyperlipidemia, metabolic syndrome, high-fasting glucose, kidney disease, insulin resistance, insulin sensitivity, and polycystic ovary syndrome.

### 3.3. Impact of Nut Consumption on Anthropometric Parameters and Energy Intake

Table 2 summarizes the effects of nut consumption on body mass, BMI, WC, and daily total energy intake. For further details, the corresponding forest plots from the meta-analysis are provided in Figures S1–S8. Overall, nut consumption did not have a significant effect on body mass, BMI, and WC, although a significant increase in energy intake was observed after nut interventions (WMD: 120 kcal/day, 95% CI: 35 to 205).

Subgroup analyses based on the type of nuts revealed that neither MUFA-rich nor PUFA-rich nuts showed significant effects on body mass, BMI, or WC. However, only the intervention involving MUFA-rich nuts resulted in a significant increase in energy intake (WMD: 101 kcal/day, 95% CI: 30 to 172). Subgroup analysis by type of control group diet indicated that, compared to CARB, ISOENERGETIC, and HYPOENERGETIC control groups, nut consumption did not affect weight, BMI, WC, or energy intake. In contrast, among studies using HABITUAL/HLS control diets, nut consumption significantly reduced body weight (WMD: −0.24 kg, 95% CI: −0.42 to −0.05) and WC (WMD: −0.49 cm, 95% CI: −0.84 to −0.13), although energy intake increased significantly (WMD: 204 kcal/day, 95% CI: 28 to 380). In articles with FAT control groups, nut consumption led to a significant decrease in energy intake (WMD: −140 kcal/day, 95% CI: 9 to 270).

### 3.4. Impact of Nut Consumption on Body Composition

Table 3 presents the effects of nut consumption on body composition, namely % fat mass, fat mass, % lean mass, lean mass and detailed forest plots from the meta-analysis are available in Figures S9–S15. Overall, nut consumption led to a significant reduction in % fat mass (WMD: −0.29%, 95% CI: −0.54 to −0.04) and fat mass (WMD: −0.41 kg, 95% CI: −0.57 to −0.26), along with increases in % lean mass (WMD: 0.71%, 95% CI: 0.32 to 1.10).

Subgroup analyses by nut type showed significant reductions in both % fat mass (WMD: −0.28%, 95% CI: −0.53 to −0.02) and fat mass (WMD: −0.42 kg, 95% CI: −0.58 to −0.27), along with increases in % lean mass (WMD: 0.71%, 95% CI: 0.32 to 1.10), within the MUFA-rich subgroup. The corresponding estimates within the PUFA-rich subgroup were not statistically significant. However, formal tests showed no significant subgroup differences for percentage fat mass (*p* = 0.439), fat mass (*p* = 0.296), or absolute lean mass (*p* = 0.993). The subgroup difference for percentage lean mass could not be estimated because all contributing comparisons involved MUFA-rich nuts. Subgroup analysis by type of control group diet revealed that, compared to CARB control group, nut consumption resulted in significant reductions in % fat mass (WMD: −0.58%, 95% CI: −0.93 to −0.23) and fat mass (WMD: −0.50 kg, 95% CI: −0.74 to −0.27). In HYPOENERGETIC groups, nut consumption significantly increased both % lean mass (WMD: 1.40%, 95% CI: 0.29 to 2.51) and lean mass (WMD: 0.97 kg, 95% CI: 0.41 to 1.53), as well as decreasing % fat mass (WMD: −1.23%, 95% CI: −2.41 to −0.06).

### 3.5. Impact of Nut Consumption on Appetite and Energy Metabolism

Table S5 shows the number of articles reporting changes (either increases or decreases) in the total number of articles assessing each outcome, including hunger, fullness, cholecystokinin (CCK), ghrelin, glucagon-like peptide-1 (GLP-1), PYY, DIT, and REE. For appetite, out of seven articles, two reported a decrease in hunger, while two reported an increase in fullness. Regarding appetite-regulating hormones, one of five articles reported an increase in CCK, two of six articles observed an increase in ghrelin, while one of six articles reported a decrease in GLP-1, and two of six articles showed an increase in PYY. For energy metabolism, two of three articles reported an increase in DIT, but no changes were observed for resting energy expenditure REE, as reported by zero of three articles.

### 3.6. Nut Consumption and Its Impact on Lipid-LipoproteinProfile

Nut consumption significantly reduced TC, LDL-C, and TG while having no significant effect on HDL-C (Table 4 and Figures S16–S23). Consuming MUFA-rich and PUFA-rich nuts both led to reductions in TC and LDL-C, with consuming MUFA-rich nuts additionally lowered TG levels. In subgroup analysis by type of control group diet, nut consumption consistently reduced TC, LDL-C, and TG, particularly when compared to CARB diets as control groups, while showing no significant changes in HDL-C across all control groups.

### 3.7. MUFA Intake from MUFA-Rich Nuts and Changes in Body Composition

**Figure 3:**
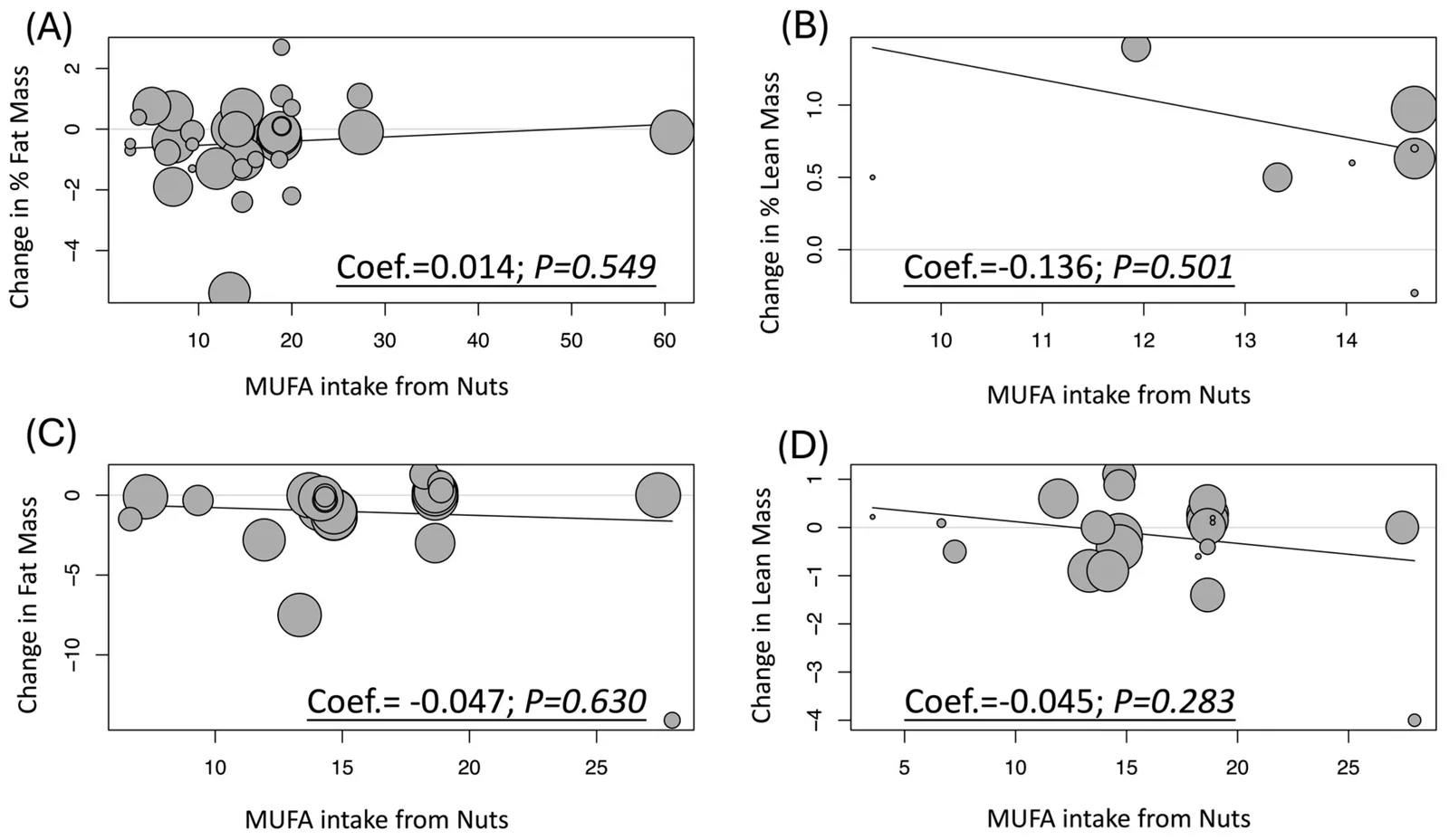
Meta-regression analysis of MUFA intake from MUFA-rich nuts and changes in body-composition parameters: (**A**) percentage fat mass (k = 28 studies; *n* = 35 data points); (**B**) percentage lean mass (k = 5 studies; *n* = 7 data points); (**C**) fat mass (kg) (k = 21 studies; *n* = 28 data points); and (**D**) lean mass (kg) (k = 11 studies; *n* = 14 data points). Each bubble represents one data point. Here, k denotes the number of distinct studies and *n* denotes the number of plotted data points; *n* may exceed k because some studies contributed more than one eligible comparison.

Meta-regression analysis on the relationship between MUFA intake per day from MUFA-rich nuts and body composition changes—body fat mass percentage, body fat mass, body lean mass, and body lean percentage—all revealed no significant correlations (Figure 3).

### 3.8. Risk of Bias, Small-Study Effects, and Certainty of Evidence

Figure S24a,b show the Cochrane risk of bias assessment for the 154 included articles, categorized by parallel and crossover designs, respectively. The results indicated that the majority of trials were assessed as having a low risk of bias (100 trials), with 45 trials classified as having some concerns, and 9 trials identified as high risk. Sensitivity analysis, performed by excluding the high-risk articles, did not result in discrepancies to the reported conclusions, thereby confirming the robustness of the findings.

Potential small-study effects were evaluated using contour-enhanced funnel plots and Egger regression for outcomes with at least 10 contributing intervention comparisons (Figure S25). Possible small-study effects were identified for energy intake (*p* = 0.046), absolute lean mass (*p* = 0.006), LDL-C (*p* = 0.001), and triglycerides (*p* = 0.039), but not for the other assessable outcomes. Percentage lean mass was not assessed because only eight comparisons contributed. Because funnel-plot asymmetry may arise from heterogeneity, methodological differences, chance, or publication bias, these findings were interpreted cautiously.

The risk-of-bias and small-study-effect assessments were incorporated into the outcome-level GRADE evaluation presented in Table S6. The certainty of evidence was rated as moderate for % fat mass, fat mass, and % lean mass; low for body weight, BMI, TC, and LDL-C; and very low for WC, energy intake, lean mass, HDL-C, and TG. Downgrading was primarily attributable to inconsistency, imprecision, and, for selected outcomes, possible small-study effects.

## 4. Discussion

Consumption of nuts, particularly those rich in unsaturated fatty acids, is widely recognized for promoting heart health and improving lipid profiles due to their nutrient-dense composition and bioactive compounds [173]. However, the specific effects of nut consumption on body composition, particularly regarding the type of dietary fats and their impact on appetite regulation and energy metabolism, remain less clear. The findings from this systematic review and meta-analysis revealed that overall nut consumption decreased body fat percentage and fat mass while increasing body lean percentage, and these results are mainly explained by MUFA-rich nut consumption. However, benefits on body composition by MUFA-rich nut consumption cannot be explained by the meta-regression, as no significant correlations were found between daily MUFA intake from nuts and changes in body composition. Additionally, significant improvements in lipid-lipoprotein profiles were observed with nut consumption, evidenced by the reductions in TC, LDL-C and TG for MUFA-rich nuts and TC and LDL-C for PUFA-rich nuts.

Albeit no changes in anthropometric parameters such as body weight, BMI, or WC were observed, there was an increase in energy intake with nut consumption. This observation may be attributed to the fact that most included studies instructed participants to consume nuts in addition to their usual diet since nuts are an inherently energy-dense food due to their high protein and fat content, thereby increasing total caloric intake [174]. However, when further investigated on body composition, the consumption of nuts generally decreased body fat percentage and fat mass. Nuts are rich in unsaturated fatty acids and they can act as ligands for PPAR, particularly PPAR-α, which leads to increased fatty-acid oxidation and energy expenditure, promoting the utilization of stored fats and contributing to a reduction in fat mass [175]. In addition to these benefits, MUFA-rich nuts further offered an increase in body lean percentage, although the increase in absolute lean mass was not statistically significant. Potential mechanisms may include the prevention of muscle atrophy and promotion muscle differentiation by enhancing mitochondrial function and reducing mitochondrial reactive oxygen species [176,177]. This, in turn, supports the maintenance and even growth of lean mass. This dual effect of MUFA, in reducing fat mass while increasing lean mass, may account for the significant improvements in body composition observed with MUFA-rich nut consumption and also effectively offsetting the additional caloric intake from nuts to maintain overall anthropometric parameters.

The systematic review indicated that only a subset of articles supported the potential role of MUFA-rich nuts in regulating appetite, appetite-related hormones, and energy metabolism. Although MUFAs were shown by past researchers to enhance satiety and stimulate appetite-related hormones such as CCK and PYY, limited data in this study did not permit a robust meta-analysis [178,179]. The limited evidence from acute intervention trials and notable methodological heterogeneity—such as differences in duration, dosage, and nut varieties—likely contributed to these inconsistent findings.

Meta-regression was conducted to further explore the relationship between MUFA intake and body composition and it did not find a linear association between MUFA intake and improvements in body composition, indicating that the amount of MUFA consumed from nuts did not explain the variation in the observed body-composition effects and does not provide evidence that MUFA itself was responsible for the findings within the MUFA-rich subgroup. Nut type was examined separately in subgroup analyses, but nut dose and nut type were not simultaneously adjusted for in the meta-regression; therefore, their independent contributions could not be determined. These subgroup findings may instead reflect other characteristics of MUFA-rich nuts, including their overall food matrix, co-occurring nutrients and bioactive compounds, comparator diets, or differences among the study populations. However, it must be noted that a limited number of contributing publications, particularly for percentage lean mass, reduces statistical power; thus, the nonsignificant findings do not exclude a dose–response relationship. While MUFAs are recognized for their beneficial role in weight management, excessive fat intake, in general, is often linked to adverse effects on body composition [180]. Therefore, the beneficial impact of MUFAs may be insufficient to counteract the negative consequences associated with excessive dietary fat consumption, highlighting the importance of considering both the quantity and context of nut consumption when assessing their health effects.

In this study, a significant decrease in TC was observed following nut consumption, consistent with findings from previous research [181]. The primary mechanism behind this reduction is likely due to the inhibitory effects of both PUFA and MUFA on the β-Hydroxy β-methylglutaryl-CoA reductase enzyme, which is a key enzyme in cholesterol synthesis, resulting in lower TC levels [182,183]. The decrease in LDL-C could be due to the increasing expression of LDL-C receptors with nut consumption [184,185]. This facilitates greater LDL-C uptake and catabolism into free fatty acids, thereby reducing circulating LDL-C concentrations [186]. The decrease in TG levels was only observed in the MUFA-rich nut group and various studies have demonstrated the role of MUFA in promoting the breakdown of triglyceride-rich lipoproteins and inhibiting hepatic secretion of very-low-density lipoprotein [187,188]. MUFA have also been reported to enhance the activity of lipoprotein lipase, an enzyme responsible for hydrolyzing triglycerides in chylomicrons and very-low-density lipoprotein particles, thereby effectively reducing TG levels [189]. No significant change in HDL-C levels was observed following nut consumption, which contrasts with findings from previous systematic reviews and meta-analyses that reported increases in HDL-C levels after consuming MUFA- and PUFA-rich foods [181,190]. Regulation of HDL-C involves complex metabolic pathways and is influenced by various factors, including genetic predisposition, hormonal status, and overall dietary patterns [191]. While the past reviews included studies with all types of food, including from the pure oil form, the modest intake of MUFA and PUFA from nuts may not be sufficient to induce significant alterations in these pathways.

Although substantial evidence supports the TG-lowering effects of PUFA, this effect was not observed in the current study [192,193]. A potential explanation could be related to findings from the control group analysis, where a decrease in TG levels was only evident when compared to the CARB control group. This aligns with the predicted changes based on dietary fat replacements, where substituting 5% of energy from carbohydrates with different types of fatty acids, including MUFA and PUFA, is known to lower TG levels [194]. However, in this study, most CARB control groups utilized MUFA-rich interventions, which may explain the observed outcomes.

Subgroup analyses based on control diet types highlighted the importance of comparator selection in randomized controlled trials investigating the effects of nut consumption. When compared to CARB control groups, nut consumption significantly reduced % fat mass, alongside consistent improvements in lipid-lipoprotein profiles and similarly in studies with HYPOENERGETIC control groups. These benefits may be attributable to the replacement of dietary carbohydrates with unsaturated fats and dietary fiber from nuts, or to reduced energy intake, both of which were shown to favorably influence lipid metabolism and body fat distribution [195,196]. Additionally, within HYPOENERGETIC control groups, nut consumption increased lean mass and % lean mass. This is likely attributable to the protein content of nuts, which could help preserve muscle mass during energy restriction as higher nut intake has been linked to improved physical fitness and reduced muscle soreness [197,198]. However, this finding was not supported by the recent systematic review and meta-analysis by Vilela et al., which reported no significant effects of nut consumption on lean mass or other anthropometric measures within energy-restricted diets, although subgroup analyses by nut type suggested small beneficial changes [199]. The discrepancy between these results could be attributed to the limited number of RCTs included in both analyses, as well as the inclusion of newer studies in this study, which were unavailable at the time of Vilela et al.’s review. Conversely, studies employing HABITUAL/HLS and FAT control groups observed reductions in waist circumference, despite increased energy intake, indicating potential compensatory dietary adjustments. This suggests that nut consumption within a stable dietary pattern may lead to compensatory reductions in other energy sources or improvements in overall diet quality [200,201]. Overall, subgroup analyses highlight that the selection of control diets and having standardized dietary controls in nut intervention trials is critical for accurately interpreting body composition and metabolic outcomes.

One of the key strengths of this systematic review, meta-analysis, and meta-regression is the comprehensive evaluation of the effects of MUFA-rich nut supplementation not only on body composition and lipid-lipoprotein profiles but also on appetite regulation and energy metabolism that can further explain body composition management. This broad scope enhances the applicability of the findings for improving various aspects of human health. Another strength of this study is the detailed subgroup assessment by control diet type, highlighting how variations in dietary comparators can lead to divergent results regarding body composition and metabolic outcomes. However, several limitations must be acknowledged. The MUFA-rich and PUFA-rich classifications were based on an operational cutoff adapted from the approximate whole-diet fatty-acid ratio proposed by Hayes [19] and not originally developed or validated for classifying individual foods. Additionally, the subgroup analysis cannot isolate the independent effect of MUFA or PUFA because nut types differ in characteristics other than fatty-acid composition, including protein, fiber, micronutrients, phytochemicals, energy density, and physical structure. Therefore, the subgroup findings should be interpreted as exploratory. Body-composition outcomes were measured using different methods, primarily dual-energy X-ray absorptiometry and bioelectrical impedance analysis. Because these methods differ in their underlying assumptions and have method-specific limitations, measurement differences may have contributed to between-study heterogeneity, and the pooled estimates should be interpreted cautiously [202]. Another limitation is that potential small-study effects should also be considered when interpreting the findings. Funnel-plot asymmetry and Egger regression suggested possible small-study effects for energy intake, lean mass, LDL-C, and TG. However, funnel-plot asymmetry is not specific to publication bias and may also arise from heterogeneity, methodological differences, or chance. Publication bias could not be formally assessed for percentage lean mass because only eight comparisons contributed. Furthermore, because some trials contributed more than one intervention comparison, the asymmetry analyses should be regarded as exploratory. These limitations, together with the wide prediction intervals and substantial heterogeneity observed for several outcomes, were reflected in the GRADE certainty assessments.

## 5. Conclusions

In conclusion, nut consumption reduced percentage fat mass and fat mass and increased percentage lean mass, while absolute lean mass did not change significantly. The most significant benefits were observed in the MUFA-rich subgroup, although subgroup differences were not significant. These effects were also not associated with MUFA intake from nuts. Both MUFA-rich and PUFA-rich nuts were effective in lowering TC and LDL-C, and MUFA-rich nuts also lowered TG, highlighting their benefits for lipid-lipoprotein metabolism. When compared to different control groups, the beneficial effect of nut consumption may vary.

## Figures and Tables

**Table 1:** PICOS criteria.

Criteria	Description
Participants	Adults with mean age ≥ 19 years
Interventions	Consumption of tree nuts and peanuts
Comparators	No tree nuts and peanuts consumption OR consumption of a different type of tree nuts and peanuts
Outcomes	Primary: body composition, appetite and energy metabolism Secondary: blood lipid-lipoprotein profile
Setting	Randomized controlled trial

**Table 2:** Effects of nut consumption on body mass, body mass index (BMI), waist circumference (WC), and total daily energy intake, with subgroup analyses by nut type (MUFA-rich and PUFA-rich) and control type (CARB, FAT, HABITUAL/HLS, HYPOENERGETIC, and ISOENERGETIC).

	WMD [95% CI]	Body Mass (kg)	BMI (kg/m^2^)	WC (cm)	Energy Intake (kcal/Day)
	Overall	0.03 [−0.25; 0.31]	−0.02 [−0.10; 0.06]	−0.46 [−0.97; 0.06]	**120** **[35; 205]**
Nut Type	MUFA-rich	0.09 [−0.25; 0.44]	−0.03 [−0.16; 0.10]	−0.50 [−1.11; 0.11]	**101** **[31; 172]**
	PUFA-rich	−0.19 [−0.41; 0.03]	0.01 [−0.03; 0.07]	−0.48 [−1.08; 0.12]	205 [−223; 632]
Control Type	CARB	0.32 [−0.59; 1.23]	−0.39 [−1.14; 0.35]	−0.75 [−1.86; 0.37]	85 [−19; 189]
	FAT	−0.07 [−2.38; 2.25]	0.10 [−0.05; 0.25]	1.07 [−0.01; 2.15]	**140** **[9; 270]**
	HABITUAL/HLS	**−0.24** **[−0.42;−0.05]**	−0.02 [−0.14;0.02]	**−0.49** **[−0.76; −0.22]**	**204** **[28; 390]**
	HYPOENERGETIC	−0.01 [−0.84; 0.82]	−0.05 [−0.45; 0.33]	0.46 [−0.70; 1.61]	85 [−56; 2256]
	ISOENERGETIC	−0.14 [−0.62; 0.34]	−0.08 [−0.54; 0.38]	−1.59 [−4.78; 1.59]	−89 [−179; 0]

Values are presented as weighted mean differences (WMDs) with 95% confidence intervals (CIs). MUFA-rich and PUFA-rich are operational subgroup classifications based on the ratio of monounsaturated fatty acids (MUFA) to polyunsaturated fatty acids (PUFA), expressed as percentages of total lipid: MUFA-rich, MUFA:PUFA ratio ≥ 1.3; PUFA-rich, MUFA:PUFA ratio < 1.3. BMI, body mass index; CARB, carbohydrate-rich control; FAT, fat-based control; HABITUAL/HLS, habitual-diet or healthy-lifestyle control; HYPOENERGETIC, energy-restricted control; ISOENERGETIC, energy- and macronutrient-matched control; WC, waist circumference. Bold values indicate statistically significant effects for which the 95% CI does not include zero.

**Table 3:** Effects of nut consumption on body composition, with subgroup analyses by nut type (MUFA-rich and PUFA-rich) and control type (CARB, FAT, HABITUAL/HLS, HYPOENERGETIC, and ISOENERGETIC).

	WMD [95% CI]	% Fat Mass (%)	Fat Mass (kg)	% Lean Mass (%)	Lean Mass ^*^ (kg)
	Overall	**−0.29** **[−0.54; −0.04]**	**−0.41** **[−0.57; −0.26]**	**0.71** **[0.32; 1.10]**	0.25 [−0.10; 0.60]
Nut Type	MUFA-rich	**−0.28** **[−0.53; −0.02]**	**−0.42** **[−0.58; −0.27]**	**0.71** **[0.32; 1.10]**	0.26 [−0.12; 0.63]
	PUFA-rich	−1.00 [−2.81; 0.81]	0.33 [−1.08; 1.75]	-	−0.15 [−1.87; 1.57]
Control Type	CARB	**−0.58** **[−0.93; −0.23]**	**−0.50** **[−0.74; −0.27]**	0.51 [−0.07; 1.09]	−0.33 [−1.17; 1.21]
	FAT	0.80 [−0.37; 1.97]	0.00 [−1.13; 1.13]	-	−0.50 [−1.79; 0.79]
	HABITUAL/HLS	−0.15 [−0.47; 0.17]	−0.04 [−0.45; 0.36]	0.21 [−1.36; 1.79]	0.13 [−0.27; 0.53]
	HYPOENERGETIC	**−1.23** **[−2.41; −0.06]**	−0.51 [−0.91; −0.10]	**1.40** **[0.29; 2.51]**	**0.97** **[0.73; 1.21]**
	ISOENERGETIC	−0.07 [−0.77; 0.62]	−0.44 [−1.05; 0.17]	**0.81** **[0.17; 1.45]**	0.55 [−0.18; 1.29]

Values are presented as weighted mean differences (WMDs) with 95% confidence intervals (CIs). MUFA-rich and PUFA-rich are operational subgroup classifications based on the ratio of monounsaturated fatty acids (MUFA) to polyunsaturated fatty acids (PUFA), expressed as percentages of total lipid: MUFA-rich, MUFA:PUFA ratio ≥ 1.3; PUFA-rich, MUFA:PUFA ratio < 1.3. CARB, carbohydrate-rich control; FAT, fat-based control; HABITUAL/HLS, habitual-diet or healthy-lifestyle control; HYPOENERGETIC, energy-restricted control; ISOENERGETIC, energy- and macronutrient-matched control. Bold values indicate statistically significant effects for which the 95% CI does not include zero. A dash indicates that no eligible comparison was available. Percentage values are expressed in percentage points. Absolute fat mass and lean mass are expressed in kilograms. * Lean mass includes fat-free mass.

**Table 4:** Effects of nut consumption on blood lipid profile, with subgroup analyses by nut type (MUFA-rich and PUFA-rich) and control type (CARB, FAT, HABITUAL/HLS, HYPOENERGETIC, and ISOENERGETIC).

	WMD [95% CI]	TC (mmol/L)	LDL-C (mmol/L)	HDL-C (mmol/L)	TG (mmol/L)
	Overall	**−0.15** **[−0.27; −0.03]**	**−0.17** **[−0.24; −0.10]**	0.02 [−0.02, 0.07]	**−0.57** **[−1.04; −0.10]**
Nut Type	MUFA-rich	**−0.16** **[−0.30; −0.01]**	**−0.16** **[−0.25; −0.08]**	0.02 [−0.04, 0.09]	**−0.63** **[−1.21; −0.05]**
	PUFA-rich	**−0.12** **[−0.22; −0.01]**	**−0.19** **[−0.25; −0.12]**	0.02 [−0.02, 0.07]	−0.06 [−0.15; 0.03]
Control Type	CARB	**−0.16** **[−0.25; −0.07]**	**−0.27** **[−0.33; −0.20]**	−0.01 [−0.04; 0.02]	**−0.17** **[−0.25; −0.10]**
	FAT	**−0.34** **[−0.55; −0.13]**	−0.12 [−0.26; 0.01]	−0.03 [−0.23; 0.17]	−0.08 [−0.21; 0.05]
	HABITUAL/HLS	−0.15 [−0.39; 0.09]	**−0.22** **[−0.34; −0.10]**	0.06 [−0.09; 0.20]	−0.93 [−1.90; 0.05]
	HYPOENERGETIC	0.15 [−0.18; 0.48]	0.14 [−0.12; 0.39]	0.05 [−0.03; 0.14]	−0.36 [−0.83; 0.11]
	ISOENERGETIC	−0.18 [−0.39; 0.02]	**−0.13** **[−0.24; −0.03]**	0.05 [−0.00; 0.10]	**−0.16** **[−0.19; −0.12]**

Values are presented as weighted mean differences (WMDs) with 95% confidence intervals (CIs), expressed in mmol/L. MUFA-rich and PUFA-rich denote nuts with monounsaturated fatty acid (MUFA):polyunsaturated fatty acid (PUFA) ratios ≥ 1.3 and <1.3, respectively. CARB, carbohydrate-rich control; FAT, fat-based control; HABITUAL/HLS, habitual-diet or healthy-lifestyle control; HYPOENERGETIC, energy-restricted control; ISOENERGETIC, energy- and macronutrient-matched control; TC, total cholesterol; LDL-C, low-density lipoprotein cholesterol; HDL-C, high-density lipoprotein cholesterol; TG, triglycerides. Bold values indicate statistically significant effects for which the 95% CI does not include zero.

## Data Availability

The original contributions presented in this study are included in the article/Supplementary Material. Further inquiries can be directed to the corresponding authors.

## Supplementary Materials

The following supporting information can be downloaded at https://www.mdpi.com/article/10.3390/foods15203594/s1: Figure S1: Random-effects meta-analysis of the weighted mean difference (WMD) in body weight (kg) following nut supplementation, with subgroup analysis by nut type; Figure S2: Random-effects meta-analysis of the WMD in body weight (kg) following nut supplementation, with subgroup analysis by control group diets; Figure S3: Random-effects meta-analysis of the WMD in BMI (kg/m^2^) following nut supplementation, with subgroup analysis by nut type; Figure S4: Random-effects meta-analysis of the WMD in BMI (kg/m^2^) following nut supplementation, with subgroup analysis by control group diets; Figure S5: Random-effects meta-analysis of the WMD in WC (cm) following nut supplementation, with subgroup analysis by nut type; Figure S6: Random-effects meta-analysis of the WMD in WC (cm) following nut supplementation, with subgroup analysis by control group diets; Figure S7: Random-effects meta-analysis of the WMD in total energy intake per day (kcal/d) following nut supplementation, with subgroup analysis by nut type; Figure S8: Random-effects meta-analysis of the WMD in total energy intake per day (kcal/d) following nut supplementation, with subgroup analysis by control group diets; Figure S9: Random-effects meta-analysis of the WMD in body fat percentage (%) following nut supplementation, with subgroup analysis by nut type; Figure S10: Random-effects meta-analysis of the WMD in body fat percentage (%) following nut supplementation, with subgroup analysis by control group diets; Figure S11: Random-effects meta-analysis of the WMD in body fat mass (kg) following nut supplementation, with subgroup analysis by nut type; Figure S12: Random-effects meta-analysis of the WMD in body fat mass (kg) following nut supplementation, with subgroup analysis by control group diets; Figure S13: Random-effects meta-analysis of the WMD in body lean percentage (%) following nut supplementation, with subgroup analysis by control group diets; Figure S14: Random-effects meta-analysis of the WMD in body lean mass (kg) following nut supplementation, with subgroup analysis by nut type; Figure S15: Random-effects meta-analysis of the WMD in body lean mass (kg) following nut supplementation, with subgroup analysis by control group diets; Figure S16: Random-effects meta-analysis of the WMD in TC (mmol/L) following nut supplementation, with subgroup analysis by nut type; Figure S17: Random-effects meta-analysis of the WMD in TC (mmol/L) following nut supplementation, with subgroup analysis by control group diets; Figure S18: Random-effects meta-analysis of the WMD in LDL-C (mmol/L) following nut supplementation, with subgroup analysis by nut type; Figure S19: Random-effects meta-analysis of the WMD in LDL-C (mmol/L) following nut supplementation, with subgroup analysis by control group diets; Figure S20: Random-effects meta-analysis of the WMD in HDL-C (mmol/L) following nut supplementation, with subgroup analysis by nut type; Figure S21: Random-effects meta-analysis of the WMD in HDL-C (mmol/L) following nut supplementation, with subgroup analysis by control group diets; Figure S22: Random-effects meta-analysis of the WMD in TG (mmol/L) following nut supplementation, with subgroup analysis by nut type; Figure S23: Random-effects meta-analysis of the WMD in TG (mmol/L) following nut supplementation, with subgroup analysis by control group diets; Figure S24: Risk-of-bias proportion graphs (percentage) for all included randomized controlled trials: (A) parallel and (B) crossover designs; Figure S25. Funnel plots for outcomes with at least 10 contributing intervention comparisons; Table S1: Search terms and results for the systematic review assessing the effects of nut consumption (September 2023); Table S2: Fatty-acid composition, calculated MUFA ratio, and operational classification of included nut types; Table S3: Summary of the 154 articles eligible for the systematic review; Table S4: Summary of articles comparing two different types of nuts; Table S5: Summary of articles reporting changes in appetite, appetite-regulating hormones, and energy metabolism in response to nut interventions; Table S6. GRADE certainty of evidence.

## Abbreviations

The following abbreviations are used in this manuscript:

ADD	Addition intervention
AHA	American Heart Association
BMI	Body mass index
CARB	Carbohydrate-rich control diet
CCK	Cholecystokinin
CI	Confidence interval
CINAHL	Cumulative Index to Nursing and Allied Health Literature
CRF	Corticotropin-releasing factor
CVD	Cardiovascular disease
CVP	Conventional peanuts
DIT	Diet-induced thermogenesis
DM	Diabetes mellitus
EVOO	Extra-virgin olive oil
FBG	Fasting blood glucose
GIP	Glucose-dependent insulinotropic polypeptide
GLP-1	Glucagon-like peptide-1
HDL-C	High-density lipoprotein cholesterol
HL	Hyperlipidemia
HMG-CoA	3-Hydroxy-3-methylglutaryl coenzyme A
LDL-C	Low-density lipoprotein cholesterol
MetS	Metabolic syndrome
MN	Mixed nuts
mTOR	Mechanistic target of rapamycin
MUFA	Monounsaturated fatty acid
NR	Not reported
OW/OB	Overweight or obese
OXM	Oxyntomodulin
PCOS	Polycystic ovary syndrome
PP	Pancreatic polypeptide
PPAR	Peroxisome proliferator-activated receptor
PRISMA	Preferred Reporting Items for Systematic Reviews and Meta-Analyses
PUFA	Polyunsaturated fatty acid
PYY	Peptide YY
RCT	Randomized controlled trial
REE	Resting energy expenditure
RER	Respiratory exchange ratio
RMR	Resting metabolic rate
RoB 2	Risk of Bias 2
SFA	Saturated fatty acid
SUB	Substitution intervention
T2D	Type 2 diabetes
TC	Total cholesterol
TG	Triglycerides
USDA	United States Department of Agriculture
VLDL	Very-low-density lipoprotein
WC	Waist circumference
WMD	Weighted mean difference

## References

[1] Unger R.H. (2003). Minireview: Weapons of Lean Body Mass Destruction: The Role of Ectopic Lipids in the Metabolic Syndrome. Endocrinology.

[2] Piché M.-E., Tchernof A., Després J.-P. (2020). Obesity Phenotypes, Diabetes, and Cardiovascular Diseases. Circ. Res..

[3] Yumuk V., Tsigos C., Fried M., Schindler K., Busetto L., Micic D., Toplak H. (2015). Obesity Management Task Force of the European Association for the Study of Obesity European Guidelines for Obesity Management in Adults. Obes. Facts.

[4] Buemann B., Sørensen T.I.A., Pedersen O., Black E., Holst C., Toubro S., Echwald S., Holst J.J., Rasmussen C., Astrup A. (2005). Lower-Body Fat Mass as an Independent Marker of Insulin Sensitivity--the Role of Adiponectin. Int. J. Obes..

[5] Zhao S., Tang J., Zhao Y., Xu C., Xu Y., Yu S., Zhang Y. (2022). The Impact of Body Composition and Fat Distribution on Blood Pressure in Young and Middle-Aged Adults. Front. Nutr..

[6] Lee D.H., Keum N., Hu F.B., Orav E.J., Rimm E.B., Willett W.C., Giovannucci E.L. (2018). Predicted Lean Body Mass, Fat Mass, and All Cause and Cause Specific Mortality in Men: Prospective US Cohort Study. BMJ.

[7] Nishi S.K., Viguiliouk E., Blanco Mejia S., Kendall C.W.C., Bazinet R.P., Hanley A.J., Comelli E.M., Salas Salvadó J., Jenkins D.J.A., Sievenpiper J.L. (2021). Are Fatty Nuts a Weighty Concern? A Systematic Review and Meta-analysis and Dose–Response Meta-regression of Prospective Cohorts and Randomized Controlled Trials. Obes. Rev..

[8] Ros E., Mataix J. (2006). Fatty Acid Composition of Nuts--Implications for Cardiovascular Health. Br. J. Nutr..

[9] Luo C., Zhang Y., Ding Y., Shan Z., Chen S., Yu M., Hu F.B., Liu L. (2014). Nut Consumption and Risk of Type 2 Diabetes, Cardiovascular Disease, and All-Cause Mortality: A Systematic Review and Meta-Analysis1234. Am. J. Clin. Nutr..

[10] Pawar A., Jump D.B. (2003). Unsaturated Fatty Acid Regulation of Peroxisome Proliferator-Activated Receptor α Activity in Rat Primary Hepatoctes*. J. Biol. Chem..

[11] Yokoi H., Mizukami H., Nagatsu A., Tanabe H., Inoue M. (2010). Hydroxy Monounsaturated Fatty Acids as Agonists for Peroxisome Proliferator-Activated Receptors. Biol. Pharm. Bull..

[12] Smith G.I., Atherton P., Reeds D.N., Mohammed B.S., Rankin D., Rennie M.J., Mittendorfer B. (2011). Omega-3 Polyunsaturated Fatty Acids Augment the Muscle Protein Anabolic Response to Hyperaminoacidemia-Hyperinsulinemia in Healthy Young and Middle Aged Men and Women. Clin. Sci..

[13] Zou H., Zheng L., Zeng C. (2025). Polyunsaturated Fatty Acids and Reduced Risk of Low Muscle Mass in Adults. Nutrients.

[14] Polley K.R., Miller M.K., Johnson M., Vaughan R., Paton C.M., Cooper J.A. (2018). Metabolic Responses to High-Fat Diets Rich in MUFA v. PUFA. Br. J. Nutr..

[15] Jones P.J.H., Jew S., AbuMweis S. (2008). The Effect of Dietary Oleic, Linoleic, and Linolenic Acids on Fat Oxidation and Energy Expenditure in Healthy Men. Metabolism.

[16] Page M.J., McKenzie J.E., Bossuyt P.M., Boutron I., Hoffmann T.C., Mulrow C.D., Shamseer L., Tetzlaff J.M., Akl E.A., Brennan S.E. (2021). The PRISMA 2020 Statement: An Updated Guideline for Reporting Systematic Reviews. BMJ.

[17] Sterne J.A.C., Savović J., Page M.J., Elbers R.G., Blencowe N.S., Boutron I., Cates C.J., Cheng H.-Y., Corbett M.S., Eldridge S.M. (2019). RoB 2: A Revised Tool for Assessing Risk of Bias in Randomised Trials. BMJ.

[18] George E.S., Daly R.M., Tey S.L., Brown R., Wong T.H.T., Tan S.-Y. (2022). Perspective: Is It Time to Expand Research on “Nuts” to Include “Seeds”? Justifications and Key Considerations. Adv. Nutr..

[19] Hayes K.C. (2002). Dietary Fat and Heart Health: In Search of the Ideal Fat. Asia Pac. J. Clin. Nutr..

[20] Abazarfard Z., Eslamian G., Salehi M., Keshavarzi S. (2016). A Randomized Controlled Trial of the Effects of an Almond-Enriched, Hypocaloric Diet on Liver Function Tests in Overweight/Obese Women. Iran. Red. Crescent Med. J..

[21] Ashwini K., Abirami K., Gayathri R., Sasikala S., Sudha V., Shobana S., Jeevan R.G., Krishnaswamy K., Deepika V., Rajalakshmi M. (2025). Effect of Premeal Pistachio Supplementation on Cardiometabolic Risk Factors among Asian Indian Adults with Prediabetes: A Randomized Controlled Trial. J. Nutr..

[22] Abazarfard Z., Salehi M., Keshavarzi S. (2014). The Effect of Almonds on Anthropometric Measurements and Lipid Profile in Overweight and Obese Females in a Weight Reduction Program: A Randomized Controlled Clinical Trial. J. Res. Med. Sci. Off. J. Isfahan Univ. Med. Sci..

[23] Huang L.-C., Henderson G.C., Mattes R.D. (2024). Effects of Daily Almond Consumption on Glycaemia in Adults with Elevated Risk for Diabetes: A Randomised Controlled Trial. Br. J. Nutr..

[24] Al Abdrabalnabi A., Rajaram S., Bitok E., Oda K., Beeson W.L., Kaur A., Cofan M., Serra-Mir M., Roth I., Ros E. (2020). Effects of Supplementing the Usual Diet with a Daily Dose of Walnuts for Two Years on Metabolic Syndrome and Its Components in an Elderly Cohort. Nutrients.

[25] Alper C.M., Mattes R.D. (2002). Effects of Chronic Peanut Consumption on Energy Balance and Hedonics. Int. J. Obes. Relat. Metab. Disord..

[26] Alvarez-Perez J., Sanchez-Villegas A., Diaz-Benitez E.M., Ruano-Rodriguez C., Corella D., Martinez-Gonzalez M.A., Estruch R., Salas-Salvado J., Serra-Majem L., Investigators P.S. (2016). Influence of a Mediterranean Dietary Pattern on Body Fat Distribution: Results of the PREDIMED-Canarias Intervention Randomized Trial. J. Am. Coll. Nutr..

[27] Alves R.D., Moreira A.P., Macedo V.S., de Cassia Goncalves Alfenas R., Bressan J., Mattes R., Costa N.M. (2014). Regular Intake of High-Oleic Peanuts Improves Fat Oxidation and Body Composition in Overweight/Obese Men Pursuing a Energy-Restricted Diet. Obesity.

[28] Arpon A., Milagro F.I., Razquin C., Corella D., Estruch R., Fito M., Marti A., Martinez-Gonzalez M.A., Ros E., Salas-Salvado J. (2017). Impact of Consuming Extra-Virgin Olive Oil or Nuts within a Mediterranean Diet on DNA Methylation in Peripheral White Blood Cells within the PREDIMED-Navarra Randomized Controlled Trial: A Role for Dietary Lipids. Nutrients.

[29] Baer D.J., Novotny J.A. (2018). Metabolizable Energy from Cashew Nuts Is Less than That Predicted by Atwater Factors. Nutrients.

[30] Baer D.J., Novotny J.A. (2019). Consumption of Cashew Nuts Does Not Influence Blood Lipids or Other Markers of Cardiovascular Disease in Humans: A Randomized Controlled Trial. Am. J. Clin. Nutr..

[31] Barbour J.A., Howe P.R., Buckley J.D., Wright G.C., Bryan J., Coates A.M. (2014). Lower Energy Intake Following Consumption of Hi-Oleic and Regular Peanuts Compared with Iso-Energetic Consumption of Potato Crisps. Appetite.

[32] Barbour J.A., Howe P.R., Buckley J.D., Bryan J., Coates A.M. (2015). Effect of 12 Weeks High Oleic Peanut Consumption on Cardio-Metabolic Risk Factors and Body Composition. Nutrients.

[33] Barbour J.A., Howe P.R.C., Buckley J.D., Bryan J., Coates A.M. (2017). Cerebrovascular and Cognitive Benefits of High-Oleic Peanut Consumption in Healthy Overweight Middle-Aged Adults. Nutr. Neurosci..

[34] Barbour J.A., Stojanovski E., Moran L.J., Howe P.R.C., Coates A.M. (2017). The Addition of Peanuts to Habitual Diets Is Associated with Lower Consumption of Savory Non-Core Snacks by Men and Sweet Non-Core Snacks by Women. Nutr. Res..

[35] Bento A.P., Cominetti C., Simoes Filho A., Naves M.M. (2014). Baru Almond Improves Lipid Profile in Mildly Hypercholesterolemic Subjects: A Randomized, Controlled, Crossover Study. Nutr. Metab. Cardiovasc. Dis..

[36] Berryman C.E., West S.G., Fleming J.A., Bordi P.L., Kris-Etherton P.M. (2015). Effects of Daily Almond Consumption on Cardiometabolic Risk and Abdominal Adiposity in Healthy Adults with Elevated LDL-Cholesterol: A Randomized Controlled Trial. J. Am. Heart Assoc..

[37] Bitok E., Rajaram S., Jaceldo-Siegl K., Oda K., Sala-Vila A., Serra-Mir M., Ros E., Sabate J. (2018). Effects of Long-Term Walnut Supplementation on Body Weight in Free-Living Elderly: Results of a Randomized Controlled Trial. Nutrients.

[38] Bowen J., Luscombe-Marsh N.D., Stonehouse W., Tran C., Rogers G.B., Johnson N., Thompson C.H., Brinkworth G.D. (2019). Effects of Almond Consumption on Metabolic Function and Liver Fat in Overweight and Obese Adults with Elevated Fasting Blood Glucose: A Randomised Controlled Trial. Clin. Nutr. ESPEN.

[39] Brown R.C., Ware L., Gray A.R., Tey S.L., Chisholm A. (2023). Comparing the Effects of Consuming Almonds or Biscuits on Body Weight in Habitual Snackers: A 1-Year Randomized Controlled Trial. Am. J. Clin. Nutr..

[40] Brown R., Ware L., Gray A.R., Chisholm A., Tey S.L. (2021). Snacking on Almonds Lowers Glycaemia and Energy Intake Compared to a Popular High-Carbohydrate Snack Food: An Acute Randomised Crossover Study. Int. J. Environ. Res. Public Health.

[41] Caldas A.P.S., Alves R.D.M., Hermsdorff H.H.M., de Oliveira L.L., Bressan J. (2020). Effects of High-Oleic Peanuts within a Hypoenergetic Diet on Inflammatory and Oxidative Status of Overweight Men: A Randomised Controlled Trial. Br. J. Nutr..

[42] Caldas A.P.S., Rocha D., Dionisio A.P., Hermsdorff H.H.M., Bressan J. (2022). Brazil and Cashew Nuts Intake Improve Body Composition and Endothelial Health in Women at Cardiometabolic Risk (Brazilian Nuts Study): A Randomized Controlled Trial. Br. J. Nutr..

[43] Carter S., Hill A.M., Buckley J.D., Tan S.Y., Rogers G.B., Coates A.M. (2023). Acute Feeding with Almonds Compared to a Carbohydrate-Based Snack Improves Appetite-Regulating Hormones with No Effect on Self-Reported Appetite Sensations: A Randomised Controlled Trial. Eur. J. Nutr..

[44] Carter S., Hill A.M., Mead L.C., Wong H.Y., Yandell C., Buckley J.D., Tan S.Y., Rogers G.B., Fraysse F., Coates A.M. (2023). Almonds vs. Carbohydrate Snacks in an Energy-Restricted Diet: Weight and Cardiometabolic Outcomes from a Randomized Trial. Obesity.

[45] Carughi A., Bellisle F., Dougkas A., Giboreau A., Feeney M.J., Higgs J. (2019). A Randomized Controlled Pilot Study to Assess Effects of a Daily Pistachio (Pistacia Vera) Afternoon Snack on Next-Meal Energy Intake, Satiety, and Anthropometry in French Women. Nutrients.

[46] Casas-Agustench P., Lopez-Uriarte P., Bullo M., Ros E., Cabre-Vila J.J., Salas-Salvado J. (2011). Effects of One Serving of Mixed Nuts on Serum Lipids, Insulin Resistance and Inflammatory Markers in Patients with the Metabolic Syndrome. Nutr. Metab. Cardiovasc. Dis..

[47] Chen C.M., Liu J.F., Li S.C., Huang C.L., Hsirh A.T., Weng S.F., Chang M.L., Li H.T., Mohn E., Chen C.O. (2017). Almonds Ameliorate Glycemic Control in Chinese Patients with Better Controlled Type 2 Diabetes: A Randomized, Crossover, Controlled Feeding Trial. Nutr. Metab..

[48] Claesson A.-L., Holm G., Ernersson A., Lindström T., Nystrom F.H. (2009). Two Weeks of Overfeeding with Candy, but Not Peanuts, Increases Insulin Levels and Body Weight. Scand. J. Clin. Lab. Investig..

[49] Coates A.M., Morgillo S., Yandell C., Scholey A., Buckley J.D., Dyer K.A., Hill A.M. (2020). Effect of a 12-Week Almond-Enriched Diet on Biomarkers of Cognitive Performance, Mood, and Cardiometabolic Health in Older Overweight Adults. Nutrients.

[50] Cofan M., Checa A., Serra-Mir M., Roth I., Valls-Pedret C., Lopez-Illamola A., Domenech M., Rajaram S., Lazaro I., Sabate J. (2024). A Walnut-Enriched Diet for 2 Years Changes the Serum Oxylipin Profile in Healthy Older Persons. J. Nutr..

[51] Cogan B., Pearson R.C., Jenkins N.T., Paton C.M., Cooper J.A. (2023). A Pecan-Enriched Diet Reduced Postprandial Appetite Intensity and Enhanced Peptide YY Secretion: A Randomized Control Trial. Clin. Nutr. ESPEN.

[52] Cogan B., Pearson R.C., Paton C.M., Jenkins N.T., Cooper J.A. (2023). Pecan-Enriched Diet Improves Cholesterol Profiles and Enhances Postprandial Microvascular Reactivity in Older Adults. Nutr. Res..

[53] Cogan B., Pearson R.C., Jenkins N.T., Paton C.M., Cooper J.A. (2023). A 4-Week Pecan-Enriched Diet Improves Postprandial Lipid Peroxidation in Aging Adults. J. Med. Food.

[54] Cohen A.E., Johnston C.S. (2011). Almond Ingestion at Mealtime Reduces Postprandial Glycemia and Chronic Ingestion Reduces Hemoglobin A(1c) in Individuals with Well-Controlled Type 2 Diabetes Mellitus. Metabolism.

[55] Damasceno N.R., Perez-Heras A., Serra M., Cofan M., Sala-Vila A., Salas-Salvado J., Ros E. (2011). Crossover Study of Diets Enriched with Virgin Olive Oil, Walnuts or Almonds. Effects on Lipids and Other Cardiovascular Risk Markers. Nutr. Metab. Cardiovasc. Dis..

[56] Damasceno N.R., Sala-Vila A., Cofan M., Perez-Heras A.M., Fito M., Ruiz-Gutierrez V., Martinez-Gonzalez M.A., Corella D., Aros F., Estruch R. (2013). Mediterranean Diet Supplemented with Nuts Reduces Waist Circumference and Shifts Lipoprotein Subfractions to a Less Atherogenic Pattern in Subjects at High Cardiovascular Risk. Atherosclerosis.

[57] Damavandi R.D., Eghtesadi S., Shidfar F., Heydari I., Foroushani A.R. (2013). Effects of Hazelnuts Consumption on Fasting Blood Sugar and Lipoproteins in Patients with Type 2 Diabetes. J. Res. Med. Sci. Off. J. Isfahan Univ. Med. Sci..

[58] Darvish Damavandi R., Mousavi S.N., Shidfar F., Mohammadi V., Rajab A., Hosseini S., Heshmati J. (2019). Effects of Daily Consumption of Cashews on Oxidative Stress and Atherogenic Indices in Patients with Type 2 Diabetes: A Randomized, Controlled-Feeding Trial. Int. J. Endocrinol. Metab..

[59] de Oliveira Fialho C.G., Moreira A.P.B., Bressan J., de Cassia Goncalves Alfenas R., Mattes R., Costa N.M.B. (2022). Effects of Whole Peanut within an Energy-Restricted Diet on Inflammatory and Oxidative Processes in Obese Women: A Randomized Controlled Trial. J. Sci. Food Agric..

[60] de Souza R.G.M., Gomes A.C., de Castro I.A., Mota J.F. (2018). A Baru Almond-Enriched Diet Reduces Abdominal Adiposity and Improves High-Density Lipoprotein Concentrations: A Randomized, Placebo-Controlled Trial. Nutrition.

[61] de Souza R.G.M., Gomes A.C., Navarro A.M., Cunha L.C.D., Silva M.A.C., Junior F.B., Mota J.F. (2019). Baru Almonds Increase the Activity of Glutathione Peroxidase in Overweight and Obese Women: A Randomized, Placebo-Controlled Trial. Nutrients.

[62] Dhillon J., Thorwald M., De La Cruz N., Vu E., Asghar S.A., Kuse Q., Diaz Rios L.K., Ortiz R.M. (2018). Glucoregulatory and Cardiometabolic Profiles of Almond vs. Cracker Snacking for 8 Weeks in Young Adults: A Randomized Controlled Trial. Nutrients.

[63] Dikariyanto V., Smith L., Francis L., Robertson M., Kusaslan E., O’Callaghan-Latham M., Palanche C., D’Annibale M., Christodoulou D., Basty N. (2020). Snacking on Whole Almonds for 6 Weeks Improves Endothelial Function and Lowers LDL Cholesterol but Does Not Affect Liver Fat and Other Cardiometabolic Risk Factors in Healthy Adults: The ATTIS Study, a Randomized Controlled Trial. Am. J. Clin. Nutr..

[64] Djoussé L., Lu B., Gaziano J.M. (2016). Effects of Walnut Consumption on Endothelial Function in People with Type 2 Diabetes: A Randomized Pilot Trial. Curr. Nutr. Rep..

[65] Dos Santos J.L., Portal V.L., Markoski M.M., de Quadros A.S., Bersch-Ferreira A., Marcadenti A. (2022). Effect of Pecan Nuts and Extra-Virgin Olive Oil on Glycemic Profile and Nontraditional Anthropometric Indexes in Patients with Coronary Artery Disease: A Randomized Clinical Trial. Eur. J. Clin. Nutr..

[66] Duarte Moreira Alves R., Boroni Moreira A.P., Silva Macedo V., Brunoro Costa N.M., Goncalves Alfenas Rde C., Bressan J. (2014). High-Oleic Peanuts Increase Diet-Induced Thermogenesis in Overweight and Obese Men. Nutr. Hosp..

[67] Dusanov S., Svendsen M., Ruzzin J., Kiviranta H., Gulseth H.L., Klemsdal T.O., Tonstad S. (2020). Effect of Fatty Fish or Nut Consumption on Concentrations of Persistent Organic Pollutants in Overweight or Obese Men and Women: A Randomized Controlled Clinical Trial. Nutr. Metab. Cardiovasc. Dis..

[68] Estruch R., Martínez-González M.Á., Corella D., Salas-Salvadó J., Ruiz-Gutiérrez V., Covas M.I., Fiol M., Gómez-Gracia E., López-Sabater M.C., Vinyoles E. (2006). Effects of a Mediterranean-Style Diet on Cardiovascular Risk Factors. Ann. Intern. Med..

[69] Fantino M., Bichard C., Mistretta F., Bellisle F. (2020). Daily Consumption of Pistachios over 12 Weeks Improves Dietary Profile without Increasing Body Weight in Healthy Women: A Randomized Controlled Intervention. Appetite.

[70] Foster G.D., Shantz K.L., Vander Veur S.S., Oliver T.L., Lent M.R., Virus A., Szapary P.O., Rader D.J., Zemel B.S., Gilden-Tsai A. (2012). A Randomized Trial of the Effects of an Almond-Enriched, Hypocaloric Diet in the Treatment of Obesity. Am. J. Clin. Nutr..

[71] Fraser G.E., Bennett H.W., Jaceldo K.B., Sabate J. (2002). Effect on Body Weight of a Free 76 Kilojoule (320 Calorie) Daily Supplement of Almonds for Six Months. J. Am. Coll. Nutr..

[72] Gayathri R., Abirami K., Kalpana N., Manasa V.S., Sudha V., Shobana S., Jeevan R.G., Kavitha V., Parkavi K., Anjana R.M. (2022). Effect of Almond Consumption on Insulin Sensitivity and Serum Lipids among Asian Indian Adults with Overweight and Obesity- A Randomized Controlled Trial. Front. Nutr..

[73] Gebauer S.K., West S.G., Kay C.D., Alaupovic P., Bagshaw D., Kris-Etherton P.M. (2008). Effects of Pistachios on Cardiovascular Disease Risk Factors and Potential Mechanisms of Action: A Dose-Response Study. Am. J. Clin. Nutr..

[74] Gepner Y., Bril N., Shelef I., Schwarzfuchs D., Serfaty D., Rein M., Cohen N., Shemesh E., Tangi-Rosental O., Sarusi B. (2016). Higher Visceral Adiposity Is Associated with an Enhanced Early Thermogenic Response to Carbohydrate-Rich Food. Clin. Nutr..

[75] Gravesteijn E., Mensink R.P., Plat J. (2023). The Effects of Long-Term Almond Consumption on Whole-Body Insulin Sensitivity, Postprandial Glucose Responses, and 48 h Continuous Glucose Concentrations in Males and Females with Prediabetes: A Randomized Controlled Trial. Eur. J. Nutr..

[76] Guarneiri L.L., Paton C.M., Cooper J.A. (2022). Changes in Body Weight in Response to Pecan-Enriched Diets with and without Substitution Instructions: A Randomised, Controlled Trial. J. Nutr. Sci..

[77] Guarneiri L.L., Paton C.M., Cooper J.A. (2022). Appetite Responses to Pecan-Enriched Diets. Appetite.

[78] Guarneiri L.L., Paton C.M., Cooper J.A. (2022). Pecan-Enriched Diets Increase Energy Expenditure and Fat Oxidation in Adults at-Risk for Cardiovascular Disease in a Randomised, Controlled Trial. J. Hum. Nutr. Diet..

[79] Gulati S., Misra A., Pandey R.M., Bhatt S.P., Saluja S. (2014). Effects of Pistachio Nuts on Body Composition, Metabolic, Inflammatory and Oxidative Stress Parameters in Asian Indians with Metabolic Syndrome: A 24-Wk, Randomized Control Trial. Nutrition.

[80] Hardman W.E., Primerano D.A., Legenza M.T., Morgan J., Fan J., Denvir J. (2019). Dietary Walnut Altered Gene Expressions Related to Tumor Growth, Survival, and Metastasis in Breast Cancer Patients: A Pilot Clinical Trial. Nutr. Res..

[81] Gulati S., Misra A., Tiwari R., Sharma M., Pandey R.M., Upadhyay A.D., Chandra Sati H. (2023). Premeal Almond Load Decreases Postprandial Glycaemia, Adiposity and Reversed Prediabetes to Normoglycemia: A Randomized Controlled Trial. Clin. Nutr. ESPEN.

[82] Gulati S., Misra A., Tiwari R., Sharma M., Pandey R.M., Upadhyay A.D., Sati H.C. (2023). Beneficial Effects of Premeal Almond Load on Glucose Profile on Oral Glucose Tolerance and Continuous Glucose Monitoring: Randomized Crossover Trials in Asian Indians with Prediabetes. Eur. J. Clin. Nutr..

[83] Hernandez-Alonso P., Salas-Salvado J., Baldrich-Mora M., Juanola-Falgarona M., Bullo M. (2014). Beneficial Effect of Pistachio Consumption on Glucose Metabolism, Insulin Resistance, Inflammation, and Related Metabolic Risk Markers: A Randomized Clinical Trial. Diabetes Care.

[84] Holligan S.D., West S.G., Gebauer S.K., Kay C.D., Kris-Etherton P.M. (2014). A Moderate-Fat Diet Containing Pistachios Improves Emerging Markers of Cardiometabolic Syndrome in Healthy Adults with Elevated LDL Levels. Br. J. Nutr..

[85] Hollingworth S., Dalton M., Blundell J.E., Finlayson G. (2019). Evaluation of the Influence of Raw Almonds on Appetite Control: Satiation, Satiety, Hedonics and Consumer Perceptions. Nutrients.

[86] Hollis J., Mattes R. (2007). Effect of Chronic Consumption of Almonds on Body Weight in Healthy Humans. Br. J. Nutr..

[87] Hou Y.Y., Ojo O., Wang L.L., Wang Q., Jiang Q., Shao X.Y., Wang X.H. (2018). A Randomized Controlled Trial to Compare the Effect of Peanuts and Almonds on the Cardio-Metabolic and Inflammatory Parameters in Patients with Type 2 Diabetes Mellitus. Nutrients.

[88] Hull S., Re R., Chambers L., Echaniz A., Wickham M.S. (2015). A Mid-Morning Snack of Almonds Generates Satiety and Appropriate Adjustment of Subsequent Food Intake in Healthy Women. Eur. J. Nutr..

[89] Hunter S.R., Considine R.V., Mattes R.D. (2022). Almond Consumption Decreases Android Fat Mass Percentage in Adults with High Android Subcutaneous Adiposity but Does Not Change HbA1c in a Randomised Controlled Trial. Br. J. Nutr..

[90] Hwang H.J., Liu Y., Kim H.S., Lee H., Lim Y., Park H. (2019). Daily Walnut Intake Improves Metabolic Syndrome Status and Increases Circulating Adiponectin Levels: Randomized Controlled Crossover Trial. Nutr. Res. Pract..

[91] Jamshed H., Sultan F.A., Iqbal R., Gilani A.H. (2015). Dietary Almonds Increase Serum HDL Cholesterol in Coronary Artery Disease Patients in a Randomized Controlled Trial. J. Nutr..

[92] Jenkins D.J., Kendall C.W., Marchie A., Parker T.L., Connelly P.W., Qian W., Haight J.S., Faulkner D., Vidgen E., Lapsley K.G. (2002). Dose Response of Almonds on Coronary Heart Disease Risk Factors: Blood Lipids, Oxidized Low-Density Lipoproteins, Lipoprotein(a), Homocysteine, and Pulmonary Nitric Oxide: A Randomized, Controlled, Crossover Trial. Circulation.

[93] Jenkins D.J.A., Kendall C.W.C., Marchie A., Josse A.R., Nguyen T.H., Faulkner D.A., Lapsley K.G., Blumberg J. (2008). Almonds Reduce Biomarkers of Lipid Peroxidation in Older Hyperlipidemic Subjects. J. Nutr..

[94] Johnston C.S., Trier C.M., Fleming K.R. (2013). The Effect of Peanut and Grain Bar Preloads on Postmeal Satiety, Glycemia, and Weight Loss in Healthy Individuals: An Acute and a Chronic Randomized Intervention Trial. Nutr. J..

[95] Jones J.L., Sabate J., Heskey C., Oda K., Miles F., Rajaram S. (2023). Macadamia Nut Effects on Cardiometabolic Risk Factors: A Randomised Trial. J. Nutr. Sci..

[96] Jung H., Chen C.O., Blumberg J.B., Kwak H.K. (2018). The Effect of Almonds on Vitamin E Status and Cardiovascular Risk Factors in Korean Adults: A Randomized Clinical Trial. Eur. J. Nutr..

[97] Kalgaonkar S., Almario R.U., Gurusinghe D., Garamendi E.M., Buchan W., Kim K., Karakas S.E. (2011). Differential Effects of Walnuts vs Almonds on Improving Metabolic and Endocrine Parameters in PCOS. Eur. J. Clin. Nutr..

[98] Kasliwal R.R., Bansal M., Mehrotra R., Yeptho K.P., Trehan N. (2015). Effect of Pistachio Nut Consumption on Endothelial Function and Arterial Stiffness. Nutrition.

[99] Katz D.L., Davidhi A., Ma Y., Kavak Y., Bifulco L., Njike V.Y. (2012). Effects of Walnuts on Endothelial Function in Overweight Adults with Visceral Obesity: A Randomized, Controlled, Crossover Trial. J. Am. Coll. Nutr..

[100] Kendall C.W., West S.G., Augustin L.S., Esfahani A., Vidgen E., Bashyam B., Sauder K.A., Campbell J., Chiavaroli L., Jenkins A.L. (2014). Acute Effects of Pistachio Consumption on Glucose and Insulin, Satiety Hormones and Endothelial Function in the Metabolic Syndrome. Eur. J. Clin. Nutr..

[101] Khorramirad A., Heidari S., Parham M., Arsang Jang S., Hozoori M. (2021). Pistachio Nut Effects on the Blood Lipid Profile in Patients With Type II Diabetes Mellitus: A Single-Blind Randomized Crossover Controlled Clinical Trial. Iran. Heart J..

[102] Kocyigit A., Koylu A.A., Keles H. (2006). Effects of Pistachio Nuts Consumption on Plasma Lipid Profile and Oxidative Status in Healthy Volunteers. Nutr. Metab. Cardiovasc. Dis..

[103] Konieczna J., Ruiz-Canela M., Galmes-Panades A.M., Abete I., Babio N., Fiol M., Martin-Sanchez V., Estruch R., Vidal J., Buil-Cosiales P. (2023). An Energy-Reduced Mediterranean Diet, Physical Activity, and Body Composition: An Interim Subgroup Analysis of the PREDIMED-Plus Randomized Clinical Trial. JAMA Netw. Open.

[104] Lasa A., Miranda J., Bullo M., Casas R., Salas-Salvado J., Larretxi I., Estruch R., Ruiz-Gutierrez V., Portillo M.P. (2014). Comparative Effect of Two Mediterranean Diets versus a Low-Fat Diet on Glycaemic Control in Individuals with Type 2 Diabetes. Eur. J. Clin. Nutr..

[105] Le T., Flatt S.W., Natarajan L., Pakiz B., Quintana E.L., Heath D.D., Rana B.K., Rock C.L. (2016). Effects of Diet Composition and Insulin Resistance Status on Plasma Lipid Levels in a Weight Loss Intervention in Women. J. Am. Heart Assoc..

[106] Lee Y.J., Nam G.E., Seo J.A., Yoon T., Seo I., Lee J.H., Im D., Bahn K.N., Jeong S.A., Kang T.S. (2014). Nut Consumption Has Favorable Effects on Lipid Profiles of Korean Women with Metabolic Syndrome. Nutr. Res..

[107] Li S.C., Liu Y.H., Liu J.F., Chang W.H., Chen C.M., Chen C.Y. (2011). Almond Consumption Improved Glycemic Control and Lipid Profiles in Patients with Type 2 Diabetes Mellitus. Metabolism.

[108] Li Z., Song R., Nguyen C., Zerlin A., Karp H., Naowamondhol K., Thames G., Gao K., Li L., Tseng C.H. (2010). Pistachio Nuts Reduce Triglycerides and Body Weight by Comparison to Refined Carbohydrate Snack in Obese Subjects on a 12-Week Weight Loss Program. J. Am. Coll. Nutr..

[109] Liu Y., Hwang H.J., Kim H.S., Park H. (2018). Time and Intervention Effects of Daily Almond Intake on the Changes of Lipid Profile and Body Composition Among Free-Living Healthy Adults. J. Med. Food.

[110] Liu Y., Hwang H.J., Ryu H., Lee Y.S., Kim H.S., Park H. (2017). The Effects of Daily Intake Timing of Almond on the Body Composition and Blood Lipid Profile of Healthy Adults. Nutr. Res. Pract..

[111] Ma Y., Njike V.Y., Millet J., Dutta S., Doughty K., Treu J.A., Katz D.L. (2010). Effects of Walnut Consumption on Endothelial Function in Type 2 Diabetic Subjects: A Randomized Controlled Crossover Trial. Diabetes Care.

[112] Mayumi Usuda Prado Rocha D., Paula Silva Caldas A., Simoes E.S.A.C., Bressan J., Hermana Miranda Hermsdorff H. (2023). Nut-Enriched Energy Restricted Diet Has Potential to Decrease Hunger in Women at Cardiometabolic Risk: A Randomized Controlled Trial (Brazilian Nuts Study). Nutr. Res..

[113] McKay D.L., Eliasziw M., Chen C.Y.O., Blumberg J.B. (2018). A Pecan-Rich Diet Improves Cardiometabolic Risk Factors in Overweight and Obese Adults: A Randomized Controlled Trial. Nutrients.

[114] Mercanligil S.M., Arslan P., Alasalvar C., Okut E., Akgul E., Pinar A., Geyik P.O., Tokgozoglu L., Shahidi F. (2007). Effects of Hazelnut-Enriched Diet on Plasma Cholesterol and Lipoprotein Profiles in Hypercholesterolemic Adult Men. Eur. J. Clin. Nutr..

[115] Mohan V., Gayathri R., Jaacks L.M., Lakshmipriya N., Anjana R.M., Spiegelman D., Jeevan R.G., Balasubramaniam K.K., Shobana S., Jayanthan M. (2018). Cashew Nut Consumption Increases HDL Cholesterol and Reduces Systolic Blood Pressure in Asian Indians with Type 2 Diabetes: A 12-Week Randomized Controlled Trial. J. Nutr..

[116] Moreira A.P.B., Teixeira T.F.S., Alves R.D.M., Peluzio M.C.G., Costa N.M.B., Bressan J., Mattes R., Alfenas R.C.G. (2016). Effect of a High-Fat Meal Containing Conventional or High-Oleic Peanuts on Post-Prandial Lipopolysaccharide Concentrations in Overweight/Obese Men. J. Hum. Nutr. Diet..

[117] Moreira Alves R.D., Boroni Moreira A.P., Macedo V.S., Bressan J., de Cassia Goncalves Alfenas R., Mattes R., Brunoro Costa N.M. (2014). High-Oleic Peanuts: New Perspective to Attenuate Glucose Homeostasis Disruption and Inflammation Related Obesity. Obesity.

[118] Morgan J.M., Horton K., Reese D., Carey C., Walker K., Capuzzi D.M. (2002). Effects of Walnut Consumption as Part of a Low-Fat, Low-Cholesterol Diet on Serum Cardiovascular Risk Factors. Int. J. Vitam. Nutr. Res..

[119] Morgan W.A., Clayshulte B.J. (2000). Pecans Lower Low-Density Lipoprotein Cholesterol in People with Normal Lipid Levels. J. Am. Diet. Assoc..

[120] Mori A.M., Considine R.V., Mattes R.D. (2011). Acute and Second-Meal Effects of Almond Form in Impaired Glucose Tolerant Adults: A Randomized Crossover Trial. Nutr. Metab..

[121] Mukuddem-Petersen J., Stonehouse Oosthuizen W., Jerling J.C., Hanekom S.M., White Z. (2007). Effects of a High Walnut and High Cashew Nut Diet on Selected Markers of the Metabolic Syndrome: A Controlled Feeding Trial. Br. J. Nutr..

[122] Ndanuko R.N., Tapsell L.C., Charlton K.E., Neale E.P., Batterham M.J. (2018). Effect of Individualised Dietary Advice for Weight Loss Supplemented with Walnuts on Blood Pressure: The HealthTrack Study. Eur. J. Clin. Nutr..

[123] Neale E.P., Tapsell L.C., Martin A., Batterham M.J., Wibisono C., Probst Y.C. (2017). Impact of Providing Walnut Samples in a Lifestyle Intervention for Weight Loss: A Secondary Analysis of the HealthTrack Trial. Food Nutr. Res..

[124] Nijssen K.M., Mensink R.P., Plat J., Ivanov D., Preissl H., Joris P.J. (2024). Mixed Nut Consumption Improves Brain Insulin Sensitivity: A Randomized, Single-Blinded, Controlled, Crossover Trial in Older Adults with Overweight or Obesity. Am. J. Clin. Nutr..

[125] Nijssen K.M.R., Mensink R.P., Plat J., Joris P.J. (2023). Longer-Term Mixed Nut Consumption Improves Brain Vascular Function and Memory: A Randomized, Controlled Crossover Trial in Older Adults. Clin. Nutr..

[126] Njike V.Y., Ayettey R., Petraro P., Treu J.A., Katz D.L. (2015). Walnut Ingestion in Adults at Risk for Diabetes: Effects on Body Composition, Diet Quality, and Cardiac Risk Measures. BMJ Open Diabetes Res. Care.

[127] Okburan G., Mercanlıgil S.M., Kabaran S., Ogmen S. (2019). Effects of Walnut-Enriched Diet on Blood Lipids and Glucose Profiles in Hyperlipidemic Subjects: A Randomized-Controlled Trial. Progr. Nutr..

[128] Osorio-Conles O., Olbeyra R., Moize V., Ibarzabal A., Giro O., Viaplana J., Jimenez A., Vidal J., de Hollanda A. (2022). Positive Effects of a Mediterranean Diet Supplemented with Almonds on Female Adipose Tissue Biology in Severe Obesity. Nutrients.

[129] Palacios O.M., Maki K.C., Xiao D., Wilcox M.L., Dicklin M.R., Kramer M., Trivedi R., Burton-Freeman B., Edirisinghe I. (2020). Effects of Consuming Almonds on Insulin Sensitivity and Other Cardiometabolic Health Markers in Adults With Prediabetes. J. Am. Coll. Nutr..

[130] Petersen K.S., Murphy J., Whitbread J., Clifton P.M., Keogh J.B. (2022). The Effect of a Peanut-Enriched Weight Loss Diet Compared to a Low-Fat Weight Loss Diet on Body Weight, Blood Pressure, and Glycemic Control: A Randomized Controlled Trial. Nutrients.

[131] Pinto X., Fanlo-Maresma M., Corbella E., Corbella X., Mitjavila M.T., Moreno J.J., Casas R., Estruch R., Corella D., Bullo M. (2019). A Mediterranean Diet Rich in Extra-Virgin Olive Oil Is Associated with a Reduced Prevalence of Nonalcoholic Fatty Liver Disease in Older Individuals at High Cardiovascular Risk. J. Nutr..

[132] Prater M.C., Guadagni A.J., Cooper J.A. (2024). Postprandial Appetite Responses to a Pecan Enriched Meal: A Randomized Crossover Trial. Appetite.

[133] Rajaram S., Connell K.M., Sabate J. (2010). Effect of Almond-Enriched High-Monounsaturated Fat Diet on Selected Markers of Inflammation: A Randomised, Controlled, Crossover Study. Br. J. Nutr..

[134] Razquin C., Martinez J.A., Martinez-Gonzalez M.A., Salas-Salvado J., Estruch R., Marti A. (2010). A 3-Year Mediterranean-Style Dietary Intervention May Modulate the Association between Adiponectin Gene Variants and Body Weight Change. Eur. J. Nutr..

[135] Reis B.Z., Duarte G.B.S., Vargas-Mendez E., Ferreira L.R.P., Barbosa F., Cercato C., Rogero M.M., Cozzolino S.M.F. (2019). Brazil Nut Intake Increases Circulating miR-454-3p and miR-584-5p in Obese Women. Nutr. Res..

[136] Reis C.E., Ribeiro D.N., Costa N.M., Bressan J., Alfenas R.C., Mattes R.D. (2013). Acute and Second-Meal Effects of Peanuts on Glycaemic Response and Appetite in Obese Women with High Type 2 Diabetes Risk: A Randomised Cross-over Clinical Trial. Br. J. Nutr..

[137] Ren M., Zhang H., Qi J., Hu A., Jiang Q., Hou Y., Feng Q., Ojo O., Wang X. (2020). An Almond-Based Low Carbohydrate Diet Improves Depression and Glycometabolism in Patients with Type 2 Diabetes through Modulating Gut Microbiota and GLP-1: A Randomized Controlled Trial. Nutrients.

[138] Riley T.M., Kris-Etherton P.M., Hart T.L., Petersen K.S. (2024). Intake of Pistachios as a Nighttime Snack Has Similar Effects on Short- and Longer-Term Glycemic Control Compared with Education to Consume 1-2 Carbohydrate Exchanges in Adults with Prediabetes: A 12-Wk Randomized Crossover Trial. J. Nutr..

[139] Rock C.L., Flatt S.W., Barkai H.S., Pakiz B., Heath D.D. (2017). A Walnut-Containing Meal Had Similar Effects on Early Satiety, CCK, and PYY, but Attenuated the Postprandial GLP-1 and Insulin Response Compared to a Nut-Free Control Meal. Appetite.

[140] Rock C.L., Flatt S.W., Barkai H.S., Pakiz B., Heath D.D. (2017). Walnut Consumption in a Weight Reduction Intervention: Effects on Body Weight, Biological Measures, Blood Pressure and Satiety. Nutr. J..

[141] Rock C.L., Flatt S.W., Pakiz B., Quintana E.L., Heath D.D., Rana B.K., Natarajan L. (2016). Effects of Diet Composition on Weight Loss, Metabolic Factors and Biomarkers in a 1-Year Weight Loss Intervention in Obese Women Examined by Baseline Insulin Resistance Status. Metabolism.

[142] Rock C.L., Zunshine E., Nguyen H.T., Perez A.O., Zoumas C., Pakiz B., White M.M. (2020). Effects of Pistachio Consumption in a Behavioral Weight Loss Intervention on Weight Change, Cardiometabolic Factors, and Dietary Intake. Nutrients.

[143] Rodrigues L.L., Cooper J.A., Paton C.M. (2019). Acute Consumption of Black Walnuts Increases Fullness and Decreases Lipid Peroxidation in Humans. Nutr. Res..

[144] Ros E., Nunez I., Perez-Heras A., Serra M., Gilabert R., Casals E., Deulofeu R. (2004). A Walnut Diet Improves Endothelial Function in Hypercholesterolemic Subjects: A Randomized Crossover Trial. Circulation.

[145] Ruisinger J.F., Gibson C.A., Backes J.M., Smith B.K., Sullivan D.K., Moriarty P.M., Kris-Etherton P. (2015). Statins and Almonds to Lower Lipoproteins (the STALL Study). J. Clin. Lipidol..

[146] Sabate J., Haddad E., Tanzman J.S., Jambazian P., Rajaram S. (2003). Serum Lipid Response to the Graduated Enrichment of a Step I Diet with Almonds: A Randomized Feeding Trial. Am. J. Clin. Nutr..

[147] Salas-Salvadó J., Fernández-Ballart J., Ros E., Martínez-González M.-A., Fitó M., Estruch R., Corella D., Fiol M., Gómez-Gracia E., Arós F. (2008). Effect of a Mediterranean Diet Supplemented with Nuts on Metabolic Syndrome Status: One-Year Results of the PREDIMED Randomized Trial. Arch. Intern. Med..

[148] Sanchis P., Molina M., Berga F., Munoz E., Fortuny R., Costa-Bauza A., Grases F., Buades J.M. (2019). A Pilot Randomized Crossover Trial Assessing the Safety and Short-Term Effects of Walnut Consumption by Patients with Chronic Kidney Disease. Nutrients.

[149] Sapp P.A., Kris-Etherton P.M., Petersen K.S. (2022). Peanuts or an Isocaloric Lower Fat, Higher Carbohydrate Nighttime Snack Have Similar Effects on Fasting Glucose in Adults with Elevated Fasting Glucose Concentrations: A 6-Week Randomized Crossover Trial. J. Nutr..

[150] Sauder K.A., McCrea C.E., Ulbrecht J.S., Kris-Etherton P.M., West S.G. (2015). Effects of Pistachios on the Lipid/Lipoprotein Profile, Glycemic Control, Inflammation, and Endothelial Function in Type 2 Diabetes: A Randomized Trial. Metabolism.

[151] Sayer R.D., Dhillon J., Tamer G.G., Cornier M.A., Chen N., Wright A.J., Campbell W.W., Mattes R.D. (2017). Consuming Almonds vs. Isoenergetic Baked Food Does Not Differentially Influence Postprandial Appetite or Neural Reward Responses to Visual Food Stimuli. Nutrients.

[152] Schutte A.E., Van Rooyen J.M., Huisman H.W., Mukuddem-Petersen J., Oosthuizen W., Hanekom S.M., Jerling J.C. (2006). Modulation of Baroreflex Sensitivity by Walnuts versus Cashew Nuts in Subjects with Metabolic Syndrome. Am. J. Hypertens..

[153] Sheridan M.J., Cooper J.N., Erario M., Cheifetz C.E. (2007). Pistachio Nut Consumption and Serum Lipid Levels. J. Am. Coll. Nutr..

[154] Singh A. (2014). Effect of Bedtime Pistachio Consumption for 6 Weeks on Body Weight, Glycemic and Lipid Profile in Obese Persons. Int. J. Food Nutr. Sci..

[155] Spiller G.A., Jenkins D.A., Bosello O., Gates J.E., Cragen L.N., Bruce B. (1998). Nuts and Plasma Lipids: An Almond-Based Diet Lowers LDL-C While Preserving HDL-C. J. Am. Coll. Nutr..

[156] Spiller G.A., Miller A., Olivera K., Reynolds J., Miller B., Morse S.J., Dewell A., Farquhar J.W. (2003). Effects of Plant-Based Diets High in Raw or Roasted Almonds, or Roasted Almond Butter on Serum Lipoproteins in Humans. J. Am. Coll. Nutr..

[157] Tan S.Y., Mattes R.D. (2013). Appetitive, Dietary and Health Effects of Almonds Consumed with Meals or as Snacks: A Randomized, Controlled Trial. Eur. J. Clin. Nutr..

[158] Tapsell L., Batterham M., Tan S.Y., Warensjo E. (2009). The Effect of a Calorie Controlled Diet Containing Walnuts on Substrate Oxidation during 8-Hours in a Room Calorimeter. J. Am. Coll. Nutr..

[159] Tapsell L.C., Batterham M.J., Teuss G., Tan S.Y., Dalton S., Quick C.J., Gillen L.J., Charlton K.E. (2009). Long-Term Effects of Increased Dietary Polyunsaturated Fat from Walnuts on Metabolic Parameters in Type II Diabetes. Eur. J. Clin. Nutr..

[160] Tapsell L.C., Gillen L.J., Patch C.S., Batterham M., Owen A., Baré M., Kennedy M. (2004). Including Walnuts in a Low-Fat/Modified-Fat Diet Improves HDL Cholesterol-to-Total Cholesterol Ratios in Patients With Type 2 Diabetes. Diabetes Care.

[161] Tey S.L., Gray A.R., Chisholm A.W., Delahunty C.M., Brown R.C. (2013). The Dose of Hazelnuts Influences Acceptance and Diet Quality but Not Inflammatory Markers and Body Composition in Overweight and Obese Individuals. J. Nutr..

[162] Tindall A.M., Petersen K.S., Skulas-Ray A.C., Richter C.K., Proctor D.N., Kris-Etherton P.M. (2019). Replacing Saturated Fat With Walnuts or Vegetable Oils Improves Central Blood Pressure and Serum Lipids in Adults at Risk for Cardiovascular Disease: A Randomized Controlled-Feeding Trial. J. Am. Heart Assoc..

[163] Wang D., Sun L., Liu X., Niu Z., Chen S., Tang L., Zheng H., Chen X., Li H., Lu L. (2021). Replacing White Rice Bars with Peanuts as Snacks in the Habitual Diet Improves Metabolic Syndrome Risk among Chinese Adults: A Randomized Controlled Trial. Am. J. Clin. Nutr..

[164] Wang S., Zhang L., Wang D., Huang M., Zhao J., Malik V., Liu X., Sun L., Lin X., Chen Y. (2021). Gut Microbiota Composition Is Associated with Responses to Peanut Intervention in Multiple Parameters Among Adults with Metabolic Syndrome Risk. Mol. Nutr. Food Res..

[165] Wang X., Li Z., Liu Y., Lv X., Yang W. (2012). Effects of Pistachios on Body Weight in Chinese Subjects with Metabolic Syndrome. Nutr. J..

[166] Wien M.A., Sabate J.M., Ikle D.N., Cole S.E., Kandeel F.R. (2003). Almonds vs Complex Carbohydrates in a Weight Reduction Program. Int. J. Obes. Relat. Metab. Disord..

[167] Wien M., Bleich D., Raghuwanshi M., Gould-Forgerite S., Gomes J., Monahan-Couch L., Oda K. (2010). Almond Consumption and Cardiovascular Risk Factors in Adults with Prediabetes. J. Am. Coll. Nutr..

[168] Wien M., Oda K., Sabaté J. (2014). A Randomized Controlled Trial to Evaluate the Effect of Incorporating Peanuts into an American Diabetes Association Meal Plan on the Nutrient Profile of the Total Diet and Cardiometabolic Parameters of Adults with Type 2 Diabetes. Nutr. J..

[169] Williams P.T., Bergeron N., Chiu S., Krauss R.M. (2019). A Randomized, Controlled Trial on the Effects of Almonds on Lipoprotein Response to a Higher Carbohydrate, Lower Fat Diet in Men and Women with Abdominal Adiposity. Lipids Health Dis..

[170] Wilson T., DeVaan L.S., LaCasse M.E., Gile E.M., Weis M.J., Ahmann M.D., Schnellman G.I., Lenz M.T., Hooks T.L. (2022). Effect of Walnut Predinner Snack on Mealtime Hunger and Nutrient Intake Among University Students. J. Med. Food.

[171] Zambón D., Sabaté J., Muñoz S., Campero B., Casals E., Merlos M., Laguna J.C., Ros E. (2000). Substituting Walnuts for Monounsaturated Fat Improves the Serum Lipid Profile of Hypercholesterolemic Men and Women. A Randomized Crossover Trial. Ann. Intern. Med..

[172] Zaveri S., Drummond S. (2009). The Effect of Including a Conventional Snack (Cereal Bar) and a Nonconventional Snack (Almonds) on Hunger, Eating Frequency, Dietary Intake and Body Weight. J. Hum. Nutr. Diet..

[173] Ros E. (2010). Health Benefits of Nut Consumption. Nutrients.

[174] Brufau G., Boatella J., Rafecas M. (2006). Nuts: Source of Energy and Macronutrients. Br. J. Nutr..

[175] Varga T., Czimmerer Z., Nagy L. (2011). PPARs Are a Unique Set of Fatty Acid Regulated Transcription Factors Controlling Both Lipid Metabolism and Inflammation. Biochim. Biophys. Acta.

[176] Xue M., Zhang F., Ji X., Yu H., Jiang X., Qiu Y., Yu J., Chen J., Yang F., Bao Z. (2021). Oleate Ameliorates Palmitate-Induced Impairment of Differentiative Capacity in C2C12 Myoblast Cells. Stem Cells Dev..

[177] Arias-Fernández L., Struijk E.A., Rodríguez-Artalejo F., Lopez-Garcia E., Lana A. (2020). Habitual Dietary Fat Intake and Risk of Muscle Weakness and Lower-Extremity Functional Impairment in Older Adults: A Prospective Cohort Study. Clin. Nutr..

[178] Cooper J.A. (2014). Factors Affecting Circulating Levels of Peptide YY in Humans: A Comprehensive Review. Nutr. Res. Rev..

[179] Stevenson J.L., Clevenger H.C., Cooper J.A. (2015). Hunger and Satiety Responses to High-Fat Meals of Varying Fatty Acid Composition in Women with Obesity. Obesity.

[180] Hooper L., Abdelhamid A.S., Jimoh O.F., Bunn D., Skeaff C.M. (2020). Effects of Total Fat Intake on Body Fatness in Adults. Cochrane Database Syst. Rev..

[181] Cao X., Xia J., Zhou Y., Wang Y., Xia H., Wang S., Liao W., Sun G. (2022). The Effect of MUFA-Rich Food on Lipid Profile: A Meta-Analysis of Randomized and Controlled-Feeding Trials. Foods.

[182] Natali F., Siculella L., Salvati S., Gnoni G.V. (2007). Oleic Acid Is a Potent Inhibitor of Fatty Acid and Cholesterol Synthesis in C6 Glioma Cells. J. Lipid Res..

[183] Das U.N. (2008). Essential Fatty Acids and Their Metabolites Could Function as Endogenous HMG-CoA Reductase and ACE Enzyme Inhibitors, Anti-Arrhythmic, Anti-Hypertensive, Anti-Atherosclerotic, Anti-Inflammatory, Cytoprotective, and Cardioprotective Molecules. Lipids Health Dis..

[184] Fernandez M.L., West K.L. (2005). Mechanisms by Which Dietary Fatty Acids Modulate Plasma Lipids1. J. Nutr..

[185] Mustad V.A., Ellsworth J.L., Cooper A.D., Kris-Etherton P.M., Etherton T.D. (1996). Dietary Linoleic Acid Increases and Palmitic Acid Decreases Hepatic LDL Receptor Protein and mRNA Abundance in Young Pigs. J. Lipid Res..

[186] Feingold K.R., Feingold K.R., Anawalt B., Blackman M.R., Boyce A., Chrousos G., Corpas E., de Herder W.W., Dhatariya K., Dungan K. (2000). Introduction to Lipids and Lipoproteins. Endotext.

[187] Kris-Etherton P.M., Zhao G., Pelkman C.L., Fishell V.K., Coval S.M. (2000). Beneficial Effects of a Diet High in Monounsaturated Fatty Acids on Risk Factors for Cardiovascular Disease. Nutr. Clin. Care.

[188] Gumbiner B., Low C.C., Reaven P.D. (1998). Effects of a Monounsaturated Fatty Acid–Enriched Hypocaloric Diet on Cardiovascular Risk Factors in Obese Patients With Type 2 Diabetes. Diabetes Care.

[189] Tholstrup T., Sandström B., Bysted A., Hølmer G. (2001). Effect of 6 Dietary Fatty Acids on the Postprandial Lipid Profile, Plasma Fatty Acids, Lipoprotein Lipase, and Cholesterol Ester Transfer Activities in Healthy Young Men123. Am. J. Clin. Nutr..

[190] Unhapipatpong C., Shantavasinkul P.C., Kasemsup V., Siriyotha S., Warodomwichit D., Maneesuwannarat S., Vathesatogkit P., Sritara P., Thakkinstian A. (2021). Tropical Oil Consumption and Cardiovascular Disease: An Umbrella Review of Systematic Reviews and Meta Analyses. Nutrients.

[191] Weissglas-Volkov D., Pajukanta P. (2010). Genetic Causes of High and Low Serum HDL-Cholesterol. J. Lipid Res..

[192] Mori T.A. (2014). Dietary N-3 PUFA and CVD: A Review of the Evidence. Proc. Nutr. Soc..

[193] Harris W.S. (2013). Are N-3 Fatty Acids Still Cardioprotective?. Curr. Opin. Clin. Nutr. Metab. Care.

[194] Hu F.B., Willett W.C. (2002). Optimal Diets for Prevention of Coronary Heart Disease. JAMA.

[195] Jenkins D.J.A., Kendall C.W.C., Lamarche B., Banach M.S., Srichaikul K., Vidgen E., Mitchell S., Parker T., Nishi S., Bashyam B. (2018). Nuts as a Replacement for Carbohydrates in the Diabetic Diet: A Reanalysis of a Randomised Controlled Trial. Diabetologia.

[196] Mead L.C., Hill A.M., Carter S., Coates A.M. (2024). Effects of Energy-Restricted Diets with or without Nuts on Weight, Body Composition and Glycaemic Control in Adults: A Scoping Review. Nutr. Res. Rev..

[197] Garrido-Miguel M., Martínez-Vizcaíno V., Fernández-Rodríguez R., Martínez-Ortega I.A., Hernández-Castillejo L.E., Bizzozero-Peroni B., Ruiz-Grao M.C., Mesas A.E. (2021). The Role of Physical Fitness in the Relationship between Nut Consumption and Body Composition in Young Adults. Nutrients.

[198] Siegel L., Rooney J., Marjoram L., Mason L., Bowles E., van Keulen T.V., Helander C., Rayo V., Hong M.Y., Liu C. (2023). Chronic Almond Nut Snacking Alleviates Perceived Muscle Soreness Following Downhill Running but Does Not Improve Indices of Cardiometabolic Health in Mildly Overweight, Middle-Aged, Adults. Front. Nutr..

[199] Vilela D.L.d.S., da Silva A., Pelissari Kravchychyn A.C., Bressan J., Hermsdorff H.H.M. (2024). Effect of Nuts Combined with Energy Restriction on the Obesity Treatment: A Systematic Review and Meta-Analysis of Randomized Controlled Trials. Foods.

[200] Witkowska A.M., Waśkiewicz A., Zujko M.E., Szcześniewska D., Śmigielski W., Stepaniak U., Pająk A., Drygas W. (2019). The Consumption of Nuts Is Associated with Better Dietary and Lifestyle Patterns in Polish Adults: Results of WOBASZ and WOBASZ II Surveys. Nutrients.

[201] Tey S.L., Brown R., Gray A., Chisholm A., Delahunty C. (2011). Nuts Improve Diet Quality Compared to Other Energy-Dense Snacks While Maintaining Body Weight. J. Nutr. Metab..

[202] Marra M., Sammarco R., De Lorenzo A., Iellamo F., Siervo M., Pietrobelli A., Donini L.M., Santarpia L., Cataldi M., Pasanisi F. (2019). Assessment of Body Composition in Health and Disease Using Bioelectrical Impedance Analysis and Dual-Energy X-Ray Absorptiometry: A Critical Overview. Contrast Media Mol. Imaging.

