# Supplementary material for: Effects of Nut Consumption on Body Composition: A Systematic Review and Meta-Analysis of Randomized Controlled Trials with Subgroup Analyses by Nut Fatty-Acid Profile
Source: Foods. 2026 Oct 10;15(20):3594. doi: 10.3390/foods15203594 (PMC13703693; doi:10.3390/foods15203594)

Table S1. Search terms and results for the systematic review assessing the effects of nuts consumption (Sept-2023)

Database	Search terms	Filter	Results
**PubMed**	(“Tree nuts” OR nut OR nuts OR "Brazil Nuts" OR Walnuts OR Almonds OR Cashews OR Hazelnuts OR "Macadamia nuts" OR Pecans OR "Pine nuts" OR Pistachios OR Peanuts OR Nuts [MESH] OR Brazil Nuts [MESH] OR Walnuts [MESH] OR Almonds [MESH] OR Cashews [MESH] OR Hazelnuts [MESH] OR Macadamia Nuts [MESH] OR Pecans [MESH] OR Pine Tree [MESH] OR Pistachios [MESH] OR Peanuts [MESH]) AND (Body Size [MESH] OR Body Mass Index [MESH] OR Body Composition [MESH] OR Waist-Hip Ratio [MESH] OR Muscle, Skeletal [MESH] OR anthropometric OR weight OR “body weight” OR “fat mass” OR “fat percentage” OR “fat volume” OR “fat body mass” OR “fat weight” OR “fat body weight” OR “body fat” OR “body fat distribution” OR “visceral fat” OR “abdominal fat” OR “lean body mass” OR “lean mass” OR “muscle mass” OR “skeletal muscle” OR “muscle thickness” OR “fat-free mass” OR “lean mass percentage” OR “muscle volume” OR “lean body weight” OR “lean weight” OR “BMI” OR “body mass index” OR “waist circumference” OR obese OR obesity OR overweight OR “waist-hip ratio” OR “hip circumference” OR appetite OR satiety OR hunger OR fullness OR “desire to eat” OR “prospective food consumption” OR “energy intake” OR “caloric intake” OR “dietary intake” OR “food intake” OR amylin OR cholecystokinin OR CCK OR “corticotropin releasing factor” OR CRF OR ghrelin OR “glucose-dependent insulinotropic polypeptide” OR GIP OR “glucagon-like peptide 1” OR GLP-1 OR glucagon OR insulin OR leptin OR oxyntomodulin OR OXM OR “pancreatic polypeptide” OR PP OR “peptide YY” OR PYY OR “transit time” OR “gastric emptying time” OR Hunger [MESH] OR Satiation [MESH] OR Energy intake [MESH] OR Food Intake [MESH] OR Ghrelin [MESH] OR Amylin [MESH] OR Cholecystokinin [MESH] OR Corticotropin Releasing Factor [MESH] OR Glucagon-Like Peptide 1 [MESH] OR Glucagon [MESH] OR Insulin [MESH] OR Leptin [MESH] OR Oxyntomodulin [MESH] OR Pancreatic Polypeptide [MESH] OR Peptide YY [MESH] OR	Human<br>English<br>Randomized Controlled Trial<br>Adult 19+	596

	thermogenesis OR "diet-induced thermogenesis" OR "thermic effect of food" OR "energy metabolism" OR "energy expenditure" OR "resting metabolic rate" OR "resting energy expenditure" OR "basal metabolism" OR "respiratory quotient" OR Energy Metabolism [MESH] OR Thermogenesis [MESH])		
**Embase**	('Tree nuts:ti,ab,kw' OR nut:ti,ab,kw OR nuts:ti,ab,kw OR 'Brazil Nuts':ti,ab,kw OR Walnuts:ti,ab,kw OR Almonds:ti,ab,kw OR Cashews:ti,ab,kw OR Hazelnuts:ti,ab,kw OR 'Macadamia nuts':ti,ab,kw OR Pecans:ti,ab,kw OR 'Pine nuts':ti,ab,kw OR Pistachios:ti,ab,kw OR Peanuts:ti,ab,kw OR 'Nuts'/exp OR 'Brazil Nuts'/exp OR 'Walnuts'/exp OR 'Almonds'/exp OR 'Cashews'/exp OR 'Hazelnuts'/exp OR 'Macadamia Nuts'/exp OR 'Pecans'/exp OR 'Pine Tree'/exp OR 'Pistachios'/exp OR 'Peanuts'/exp) AND ('Body Size'/exp OR 'Body Mass Index'/exp OR 'Body Composition'/exp OR 'Waist-Hip Ratio'/exp OR 'skeletal muscle'/exp OR anthropometric:ti,ab,kw OR weight:ti,ab,kw OR 'body weight':ti,ab,kw OR 'fat mass':ti,ab,kw OR 'fat percentage':ti,ab,kw OR 'fat volume':ti,ab,kw OR 'fat body mass':ti,ab,kw OR 'fat weight':ti,ab,kw OR 'fat body weight':ti,ab,kw OR 'body fat':ti,ab,kw OR 'body fat distribution':ti,ab,kw OR 'visceral fat':ti,ab,kw OR 'abdominal Fat':ti,ab,kw OR 'lean body mass':ti,ab,kw OR 'lean mass':ti,ab,kw OR 'muscle mass':ti,ab,kw OR 'skeletal muscle':ti,ab,kw OR 'muscle thickness':ti,ab,kw OR 'fat-free mass':ti,ab,kw OR 'lean mass percentage':ti,ab,kw OR 'muscle volume':ti,ab,kw OR 'lean body weight':ti,ab,kw OR 'lean weight':ti,ab,kw OR 'BMI':ti,ab,kw OR 'body mass index':ti,ab,kw OR 'waist circumference':ti,ab,kw OR 'obese':ti,ab,kw OR obesity:ti,ab,kw OR overweight:ti,ab,kw OR 'waist-hip ratio':ti,ab,kw OR 'hip circumference':ti,ab,kw OR appetite:ti,ab,kw OR satiety:ti,ab,kw OR hunger:ti,ab,kw OR fullness:ti,ab,kw OR 'desire to eat':ti,ab,kw OR 'prospective food consumption':ti,ab,kw OR 'energy intake':ti,ab,kw OR 'caloric intake':ti,ab,kw OR 'dietary intake':ti,ab,kw OR 'food intake':ti,ab,kw OR amylin:ti,ab,kw OR cholecystokinin:ti,ab,kw OR CCK:ti,ab,kw OR 'corticotropin releasing factor':ti,ab,kw OR CRF:ti,ab,kw OR ghrelin:ti,ab,kw OR 'glucose-dependent insulinotropic polypeptide':ti,ab,kw OR GIP:ti,ab,kw OR 'glucagon-like peptide	Adults<br>Human<br>Exclude<br>MEDLINE<br>records<br>Randomized<br>Controlled Trial	202

	1’:ti,ab,kw OR GLP-1:ti,ab,kw OR glucagon:ti,ab,kw OR insulin:ti,ab,kw OR leptin:ti,ab,kw OR oxyntomodulin:ti,ab,kw OR OXM:ti,ab,kw OR ‘pancreatic polypeptide’:ti,ab,kw OR PP:ti,ab,kw OR ‘peptide YY’:ti,ab,kw OR PYY:ti,ab,kw OR ‘transit time’:ti,ab,kw OR ‘gastric emptying time’:ti,ab,kw OR ‘hunger’/exp OR ‘Satiation’/exp OR ‘Energy intake’/exp OR ‘Food Intake’/exp OR ‘Ghrelin’/exp OR ‘Amylin’/exp OR ‘cholecystokinin’/exp OR ‘Corticotropin Releasing Factor’/exp OR ‘Glucagon-Like Peptide 1’/exp OR ‘glucagon’/exp OR ‘Insulin’/exp OR ‘Leptin’/exp OR ‘oxyntomodulin’/exp OR ‘Pancreatic Polypeptide’/exp OR ‘Peptide YY’/exp OR thermogenesis:ti,ab,kw OR ‘diet-induced thermogenesis’:ti,ab,kw OR ‘thermic effect of food’:ti,ab,kw OR ‘energy metabolism’:ti,ab,kw OR ‘energy expenditure’:ti,ab,kw OR ‘resting metabolic rate’:ti,ab,kw OR ‘resting energy expenditure’:ti,ab,kw OR ‘basal metabolism’:ti,ab,kw OR ‘respiratory quotient’:ti,ab,kw OR ‘Energy Metabolism’/exp OR ‘Thermogenesis’/exp)		
**CINAHL**	((AB (“Tree nuts”) OR (nut) OR (nuts) OR ("Brazil Nuts") OR (Walnuts) OR (Almonds) OR (Cashews) OR (Hazelnuts) OR ("Macadamia nuts") OR (Pecans) OR ("Pine nuts") OR (Pistachios) OR (Peanuts)) OR (MH Nuts) OR (MH Brazil Nuts) OR (MH Walnuts) OR (MH Almonds) OR (MH Cashews) OR (MH Hazelnuts) OR (MH Macadamia Nuts) OR (MH Pecans) OR (MH Pine Tree) OR (MH Pistachios) OR (MH Peanuts)) AND ((MH Body Size) OR (MH Body Mass Index) OR (MH Body Composition) OR (MH Waist-Hip Ratio) OR (MH Muscle, Skeletal) OR (AB (anthropometric) OR (weight) OR (“body weight”) OR (“fat mass”) OR (“fat percentage”) OR (“fat volume”) OR (“fat body mass”) OR (“fat weight”) OR (“fat body weight”) OR (“body fat”) OR (“body fat distribution”) OR (“visceral fat”) OR (“abdominal fat”) OR (“lean body mass”) OR (“lean mass”) OR (“muscle mass”) OR (“skeletal muscle”) OR (“muscle thickness”) OR (“fat-free mass”) OR (“lean mass percentage”) OR (“muscle volume”) OR (“lean body weight”) OR (“lean weight”) OR (“BMI”) OR (“body mass index”) OR (“waist circumference”) OR (obese) OR (obesity) OR (overweight) OR (“waist-hip ratio”) OR (“hip circumference”) OR (appetite) OR (satiety) OR (hunger) OR (fullness) OR (“desire to eat”) OR (“prospective food consumption”) OR (“energy intake”) OR (“caloric intake”)	Human<br>English<br>Exclude MEDLINE records<br>Randomized Controlled Trial<br>All adults	80

	OR (“dietary intake”) OR (“food intake”) OR (amylin) OR (cholecystokinin) OR (CCK) OR (“corticotropin releasing factor”) OR (CRF) OR (ghrelin) OR (“glucose-dependent insulinotropic polypeptide”) OR (GIP) OR (“glucagon-like peptide 1”) OR (GLP-1) OR (glucagon) OR (insulin) OR (leptin) OR (oxyntomodulin) OR (OXM) OR (“pancreatic polypeptide”) OR (PP) OR (“peptide YY”) OR (PYY) OR (serotonin) OR (“transit time”) OR (“gastric emptying time”)) OR (MH hunger) OR (MH Satiation) OR (MH Energy intake) OR (MH Food Intake) OR (MH Ghrelin) OR (MH Amylin) OR (MH cholecystokinin) OR (MH Corticotropin Releasing Factor) OR (MH Glucagon-Like Peptide 1) OR (MH glucagon) OR (MH Insulin) OR (MH Leptin) OR (MH oxyntomodulin) OR (MH Pancreatic Polypeptide) OR (MH Peptide YY) OR (AB (thermogenesis) OR (“diet-induced thermogenesis”) OR (“thermic effect of food”) OR (“energy metabolism”) OR (“energy expenditure”) OR (“resting metabolic rate”) OR (“resting energy expenditure”) OR (“basal metabolism”) OR (“respiratory quotient”)) OR (MH Energy Metabolism) OR (MH Thermogenesis))		
**Cochrane**	((anthropometric OR weight OR “body weight” OR (MeSH “body weight”) OR “fat mass” OR “fat percentage” OR “fat mass” OR “fat percentage” OR “fat volume” OR “fat body mass” OR “fat weight” OR “fat body weight” OR “body fat” OR “body fat distribution” OR “visceral fat” OR “abdominal fat” OR “lean body mass” OR “lean mass” OR “muscle mass” OR “skeletal muscle” OR (MeSH “skeletal muscle”) OR (MeSH “skeletal muscle”) OR “muscle thickness” OR “fat-free mass” OR “lean mass percentage” OR “muscle volume” OR “lean body weight” OR “lean weight” OR (MeSH “body composition”) OR “BMI” OR “body mass index” OR “waist circumference” OR obese OR obesity OR overweight OR “body fat” OR “waist-hip ratio” OR (MeSH “waist-hip ratio”) OR “hip circumference” OR appetite OR satiety OR (MeSH “satiety”) OR hunger OR (MeSH “hunger”) OR fullness OR “desire to eat” OR (MeSH “eating”) OR “prospective food consumption” OR “energy intake” OR (MeSH “energy intake”) OR “caloric intake” OR “dietary intake” OR “food intake” OR amylin OR (MeSH “amylin”) OR cholecystokinin OR (MeSH “cholecystokinin”) OR CCK OR “corticotropin releasing factor” OR (MeSH “corticotropin releasing factor”) OR CRF OR ghrelin OR	-	2187

(MeSH “ghrelin”) OR “glucose-dependent insulinotropic polypeptide” OR GIP OR “glucagon-like peptide 1” OR (MeSH “glucagon-like peptide 1”) OR GLP-1 OR glucagon OR (MeSH “glucagon”) OR insulin OR (MeSH “insulin”) OR leptin OR (MeSH “leptin”) OR oxyntomodulin OR (MeSH “oxyntomodulin”) OR OXM OR “pancreatic polypeptide” OR (MeSH “pancreatic polypeptide”) OR PP OR “peptide YY” OR (MeSH “peptide YY”) OR PYY OR “transit time” OR “gastric emptying time” OR thermogenesis OR (MeSH “thermogenesis”) OR “diet-induced thermogenesis” OR “thermic effect of food” OR “energy metabolism” OR (MeSH “energy metabolism”) OR “energy expenditure” OR “resting metabolic rate” OR “resting energy expenditure” OR “Basal Metabolism” OR “respiratory quotient”) AND (“Tree nuts” OR nut OR nuts OR (MeSH “nuts”) OR “Brazil Nuts” OR (MeSH “Brazil Nuts”) OR Walnuts OR (MeSH “Walnuts”) OR Almonds OR (MeSH “Almonds”) OR Cashews OR (MeSH “Cashews”) OR Hazelnuts OR (MeSH “Hazelnuts”) OR "Macadamia nuts" OR (MeSH “Macadamia nuts”) OR Pecans OR (MeSH “Pecans”) OR "Pine nuts" OR (MeSH “Pine nuts”) OR Pistachios OR (MeSH “Pistachios”) OR Peanuts OR (MeSH “Peanuts”)))	
**Total**	**3065**

Table S2: Fatty-acid composition, calculated MUFA ratio, and operational classification of included nut types

Nut type	SFA (% of lipid)	MUFA (% of lipid)	PUFA (% of lipid)	MUFA:PUFA ratio	SFA:MUFA:PUFA	Classification
**Almonds**	8.148	61.667	25.556	2.41	0.3:2.4:1	MUFA-rich
**Brazil nuts**	23.994	35.618	36.364	0.98	0.7:1.0:1	PUFA-rich
**Cashews**	19.375	57.917	18.333	3.16	1.1:3.2:1	MUFA-rich
**Hazelnuts**	7.401	75.164	12.993	5.78	0.6:5.8:1	MUFA-rich
**Macadamia nuts**	15.637	77.924	1.971	39.54	7.9:39.5:1	MUFA-rich
**Pecans**	8.791	56.044	30.495	1.84	0.3:1.8:1	MUFA-rich
**Pine nuts**	7.164	27.485	49.854	0.55	0.1:0.6:1	PUFA-rich
**Pistachios**	12.447	52.954	29.958	1.77	0.4:1.8:1	MUFA-rich
**Walnuts**	9.356	13.650	72.393	0.19	0.1:0.2:1	PUFA-rich
**Peanuts**	15.493	52.716	19.718	2.67	0.8:2.7:1	MUFA-rich

SFA, MUFA, and PUFA values are expressed as percentages of total lipid and were calculated from the fatty-acid composition reported by USDA. The MUFA ratio was calculated as MUFA divided by PUFA. Nuts with a MUFA ratio ≥1.3 were operationally classified as MUFA-rich, and those with a ratio <1.3 were classified as PUFA-rich. The cutoff was adapted from the MUFA and PUFA components of the approximate whole-diet SFA:MUFA ratio of 1:1.3:1 discussed by Hayes [19].

Table S3. Summary of the 154 articles eligible for systematic review

Trial	Heath Status	Age Mean	Design	Duration	N	Dose	Group size, no	Reported Outcome
**Abazarfard et al. 2014**	OW or OB	43	P	12	108[100]<br>0M: 108F	50g Almonds<br>Isocaloric	50<br>50	BMI↓, body fat↓, body weight↓, FBG↓, lipid profile, WC↓
**Abazarfard et al. 2016**	OW or OB	43	P	12	108[100]<br>0M: 108F	50g Almonds<br>Isocaloric	50<br>50	BMI↓, WC↓
**Abdrabalnabi et al. 2019**	H	69	P	48	698[625]<br>279M:419	45g Walnuts<br>Habitual	324<br>312	BMI↓, FBG↓, lipid profile
**Alisa et al. 2011**	T2DM	39	C	5	14[14]<br>8M:6F	42.5g Almonds<br>FAT	14<br>14	FBG↓, insulin↑
**Alper et al. 2002**	H	33	P	31	15[15]<br>8M:7F	P: FF<br>P: ADD<br>P: SUB	15<br>15<br>15	energy intake↑
**Alvarez et al. 2016**	T2DM or CVD	68	P	52	531[305]<br>121M:230	30g MN<br>1L/wk EVOO	102<br>112	BMI, body fat, body weight, energy intake, lipid profile, WC↓
**Alves et al. 2014**	OW or OB	34	P	4	76[65]<br>65M:0F	56g CVP<br>56g HOP<br>Isoenergetic	22<br>21<br>22	BMI↓, body fat↓, body weight↓, energy intake, WC↓
**Arpon et al. 2018**	H	64	P	52	24[24]<br>12:12	30g MN<br>1L/wk EVOO	12<br>12	BMI, body fat, body weight, FBG, lipid profile,WC
**Ashwini et al. 2025**	Prediabetes	41	P	12	120[109]<br>54M:45F	60g Pistachio<br>Isoenergetic	56<br>53	BMI, body weight, lipid profile, WC↓

**Baer et al. 2018**	H	57	C	4	18[18]<br>9M:9F	42g Cashew<br>Isoenergetic	NR	body weight, energy intake
**Baer et al. 2019**	H	57	C	4	42[40]<br>9M:9F	42g Cashew<br>Isoenergetic	20<br>20	body weight, lipid profile
**Barbour et al. 2014**	OW or OB	61	C	3	29[24]<br>13M:11F	70g CVP<br>75g HOP<br>CARB	6<br>8<br>10	appetite, fullness, hunger
**Barbour et al. 2015**	H	65	C	6	67[61]<br>29M:32F	69g peanuts<br>Nut-Free	32<br>29	BMI↑, body weight, energy intake↑, FBG, insulin, lipid profile, WC
**Barbour et al. 2016**	H	65	C	6	67[61]<br>29M:32F	69g peanuts<br>Nut-Free	32<br>29	BMI↑, energy intake↑
**Barbour et al. 2017**	H	65	C	6	67[61]<br>29M:32F	69g peanuts<br>Nut-Free	32<br>29	body weight↑, energy expenditure, energy intake↑
**Bento et al. 2014**	HC	35	C	6	25[20]<br>8M:12F	20g Almonds<br>Nut-Free	20<br>20	BMI, body fat, body weight, energy intake, lipid profile
**Berryman et al. 2015**	OW or OB	48	C	6	61[48]<br>22M:26F	42.5g Almonds<br>CARB	48<br>48	BMI, body fat, body weight, energy intake, FBG, lipid profile, WC↑
**Bitok et al. 2018**	H	69	P	104	356[307]<br>119M:237F	42g Walnuts<br>Habitual	39<br>37	BMI, body fat↑, body weight↓, energy intake, WC
**Bowen et al. 2018**	HC	35	C	6	25[20]<br>8M:12F	20g Almonds<br>Nut-Free	20<br>20	BMI, body fat, body weight, energy intake, lipid profile
**Brown et al. 2021**	H	29	C	NR	106[100]<br>25M:75F	42.5g Almonds<br>Isocaloric	50<br>50	appetite, hunger, fullness, energy intake↓

**Brown et al. 2023**	H	36	P	52	136[125]<br>35M:101F	42.5g Almonds<br>CARB	57<br>52	body weight, lipid profile, WC
**Caldas et al. 2020**	OW or OB	27	P	4	76[64]<br>64M:0F	56g CVP<br>56g HOP<br>Hypoenergeyic	21<br>21<br>22	BMI, body weight↓, WC↓, energy intake
**Caldas et al. 2022**	OW or OB	31	P	8	40[29]<br>0M:29F	30g MN<br>Hypoenergeyic	14<br>15	BMI, body fat, body weight↓, energy intake↑, FBG, insulin, lipid profile, WC↓
**Carter et al. 2023**	OW or OB	48	P	36	147[140]<br>42M:98F	40g Amonds<br>CARB	68<br>72	CCK, Ghrelin, GLP-1, Glucagon, leptin, PP, PYY
**Carter et al. 2023**	OW or OB	48	P	36	140[120]<br>42M:98F	40g Amonds<br>CARB	68<br>72	BMI, body fat↓, body weight↓, energy intake↓, FBG↓, insulin↓, lipid profile, WC
**Carughi et al. 2019**	H	35	P	4	60[60]<br>0M:60F	56g Pistachio<br>Isoenergetic	30<br>30	BMI, body fat, body weight, energy intake↓, FBG, WC
**Casas et al. 2019**	MetS	52	P	12	52[50]<br>28M:22F	30g MN<br>Nut-Free	25<br>25	body fat, body weight↓, energy intake, FBG, insulin, lipid profile, WC↓
**Chen et al. 2017**	T2DM	55	C	12	40[33]<br>13M:20F	60g Almonds<br>Hypoenergeyic	33<br>33	BMI, body fat, body weight, FBG, insulin, lipid profile, WC
**Claesson et al. 2009**	H	23	P	2	26[25]<br>11M:14F	232g peanuts<br>CARB	13<br>12	BMI ↑, body fat, body weight↑, energy intake↑, FBG, insulin↑, lipid profile, WC↑
**Coates et al. 2020**	OW or OB	65	P	12	151[128]<br>70M:78F	Almonds<br>Isoenergetic	63<br>65	BMI, body fat, body weight, energy intake, FBG, insulin, lipid profile, WC
**Cogan et al. 2023**	H	59	P	4	49[44]<br>13M:31F	68g Pecan<br>Isoenergetic	21<br>23	appetite, BMI, body fat, body weight, energy intake, WC, PYY↑, CCK↑, ghrelin↑
**Cogan et al. 2023**	H	59	P	4	49[44]	68g Pecan	21	

					13M:31F	Isoenergetic	23	BMI, body weight, energy intake↑, lipid profile, WC
**Cogan et al. 2024**	H	59	P	4	49[41]<br>13M:31F	68g Pecan<br>Isoenergetic	21<br>20	glucose, insulin
**Cofán et al. 2024**	H	68	W	104	115[115]<br>35M:80F	15% energy<br>Habitual	64<br>51	energy intake↑
**Cohen et al. 2011**	H or T2D	66	P	12	20[19]<br>6M:14F	28g Almonds<br>Isoenergetic	6<br>7	BMI↓, body fat, body weight, energy intake, FBG ↓, insulin, lipid profile, nutrients intake, WC
**Conles et al. 2022**	OW or OB	47	P	12	38[36]<br>0M:36F	30g Almonds<br>Habitual	19<br>17	BMI, body fat, body weight, FBG, insulin, lipid profile
**Damasceno et al. 2011**	HC	56	C	4	26[18]<br>9M:9F	65g Walnuts<br>62.5g Almonds<br>FAT	18<br>18<br>18	BMI, body weight, FBG, lipid profile
**Damasceno et al. 2013**	CVD	67	P	52	117[117]<br>53M:64F	30g MN<br>1L/wk EVOO	58<br>59	BMI, body fat, body weight, energy intake, FBG, insulin, lipid profile, nutrients intake, WC↓
**Damavandi et al. 2014**	T2DM	NR	P	8	50[48]<br>15M:33F	29g Hazelnuts<br>Habitual	23<br>25	BMI, body fat, body weight, energy intake, FBG, insulin, lipid profile, nutrients intake
**Damavandi et al. 2019**	T2DM	54	P	8	50[43]<br>9M:34F	28g Cashews<br>Habitual	22<br>21	BMI, body fat, body weight, energy intake, FBG, insulin, lipid profile, nutrients intake
**Dikariyanto et al. 2020**	CVD	56	P	6	107[105]<br>32M:75F	Almond<br>CARB	54<br>51	BMI, body fat, energy intake, FBG, insulin, lipid profile, nutrients intake, WC
**Djousse et al. 2016**	DM	65	P	12	27[26]	28g Walnuts	13	

					10M:16F	Habitual	13	BMI, body weight, energy intake, nutrients intake
**Dos Santos et al. 2022**	CVD	60	P	12	204[204]<br>155M:49F	30g Pecan<br>HLS	68<br>67	BMI, body weight, FBG, insulin
**Duarte et al. 2014**	OW or OB	27	P	NR	71[71]<br>71M:0F	56g CVP<br>56 HOP<br>Nut-Free	23<br>24<br>24	DIT↑, fullness, hunger, REE, satiety
**Dusanov et al. 2020**	OW or OB	57	P	24	87[76]<br>37M:50F	200g MN<br>Habitual/Nut-Free	45<br>42	body fat, body weight, energy intake, FBG, insulin, lipid profile, WC
**Estruch et al. 2006**	CVD	69	P	12	515[514]<br>339M:176F	30g MN<br>1L/wk EVOO	257<br>257	BMI, body weight, energy intake, FBG, HC, insulin, lipid profile, WC
**Estruch et al. 2019**	CVD	67	P	208	4904[2484<br>2178M:306F	30g MN<br>1L/wk EVOO	129<br>113	body weight, WC↓
**Fantino et al. 2020**	H	32	P	16	63[60]<br>0M:60F	44g Peanuts<br>Habitual	30<br>30	body weight↓
**Fialho et al. 2022**	OW or OB	33	P	8	26[24]<br>0M:24F	56g whole Peanuts<br>56g skinned Peanuts<br>Habitual	8<br>8<br>8	BMI↓, body fat↓, body weight↓, energy intake↓, FBG, insulin, lipid profile, WC↓
**Foster et al. 2012**	OW or OB	47	P	72	123[123]<br>11M:24F	56g Almonds<br>Hypoenergeyic	61<br>62	body fat, body weight, lean mass↓, lipid profile
**Fraser et al. 2002**	H	49	C	8	100[81]<br>43M:38F	Almonds<br>Habitual	NR	body weight
**Gayathri et al. 2023**	OW or OB	38	P	12	400[352]<br>NR	43g Almonds<br>Habitual/Nut-Free	174<br>178	BMI, body weight↓, energy intake, FBG↓, lipid profile, WC↓

**Gebauer et al. 2008**	HLDL	48	C	4	29[28]<br>10M:18F	47.5g Pistachio<br>94.5g Pistachio<br>LOW FAT	28<br>28<br>28	body weight, lipid profile
**Gepner et al. 2016**	OW or OB	45	C	3	40[40]<br>40M:0F	56g Walnuts<br>CARB	40<br>40	REE
**Gozde et al. 2019**	HL	43	P	6	39[37]<br>11M:6F	40g Walnuts<br>HLS/Nut-Free	20<br>17	BMI, body fat, body weight, FBG↓, insulin↓, lipid profile, WC
**Gravesteijn et al. 2023**	DM	66	C	20	43[34]<br>22M:12F	50g Almonds<br>HLS/Nut-Free	34<br>34	BMI↑, FBG, insulin, lipid profile, WC↑
**Guarneiri et al. 2021**	HC or OW or OB	48	P	8	56[47]<br>14M:33F	68g ADD Pecan<br>68g SUB Pecan<br>FAT	17<br>19<br>20	RMR↑, RER, DIT↑
**Guarneiri et al. 2022**	HC or OW or OB	48	P	8	56[47]<br>14M:33F	68g ADD Pecan<br>68g SUB Pecan<br>FAT	15<br>16<br>16	CCK, ghrelin, PYY
**Guarneiri et al. 2021**	H	46	P	8	106[93]<br>29M:64F	68g ADD Pecan<br>68g SUB Pecan<br>Nut-Free	30<br>31<br>32	BMI, body fat, body weight, energy intake, WC
**Gulati et al. 2014**	MetS	43	P	24	68[60]<br>37M:31F	Pistachio<br>LOW FAT	33<br>35	body fat, body weight, FBG↑, insulin, lipid profile, WC↓
**Gulati et al. 2023**	T2DM	42	P	12	66[60]<br>28M:38F	60g Almonds<br>Nut-Free	30<br>30	BMI, body fat, body weight↓, FBG↓, insulin, lipid profile, WC
**Gulati et al. 2023**	Prediabetes	41	C	4	60[60]<br>27M:33F	60g Almonds<br>Nut-Free	30<br>30	FBG↓, glucagon↓, insulin↓

**Hardman et al. 2019**	Breast canccer	54	P	2	38[14]<br>0M:14F	56.7g Walnuts<br>Habitual	7<br>7	body weight
**Hern´andez-Alonso et al. 2014**	Prediabetic	55	C	32	54[49]<br>29F:25M	57g Pistachio<br>FAT	54<br>54	BMI, body weight, FBG↓, insulin↓, lipid profile, WC
**Holligan et al. 2014**	H	48	C	6	28[28]<br>10M:18F	75g Pistachio<br>150 Pistachio<br>LOW FAT	28<br>28<br>28	body weight, BMI, FBG, insulin
**Hollingwort et al. 2019**	H	26	C	8	42[42]<br>0M:42F	52.7g Almonds<br>CARB	NR	appetite, energy intake
**Hollis et al. 2007**	H	24	C	10	24[20]<br>NR	Almonds<br>Habitual	20<br>20	BMI, body fat, body weight
**Hou et al. 2018**	T2DM	67	P	12	32[25]<br>15M:10F	60g Peanuts<br>55g Almods	11<br>14	BMI, FBG↓, lipid profile, WC
**Hull et al. 2015**	H	48	C	12	32[32]<br>0M:32F	28g Almonds<br>Nut-Free	32<br>32	energy intake, fullness, hunger
**Hunter et al. 2022**	H	37	P	24	134[134]<br>33M:101F	42.52g Almonds<br>Habitual	69<br>65	appetite, body fat, body weight, energy intake, FBG↓, insulin, lipid profile
**Huang et al. 2024**	High risk for T2DM	49	P	16	81[74]<br>23M:58F	45g Walnuts<br>CARB	39<br>42	body fat, body lean, BMI, body weight, energy intake↑,FBG
**Hwang et al. 2019**	MetS	39	C	16	119[84]<br>42M:42F	45g Walnuts<br>CARB	84<br>84	energy intake, FBG, insulin, lipid profile↑, WC
**Dhillon et al. 2018**	H	36	P	8	80[73]<br>32M:41F	56.7g Almonds<br>CARB	38<br>35	appetite, body fat, body weight, energy intake, FBG, insulin, lipid profile↓, nutrients intake, WC

**Jamshed et al. 2015**	CVD	NR	P	12	150[113]<br>113M:37F	10g Pakistani Almonds<br>10g American almonds<br>Nut-Free	38<br>41<br>34	body weight, lipid profile
**Jenkins et al. 2002**	H	64	C	4	43[27]<br>15F:12M	73g Almonds<br>37g Almonds<br>CRAB	27<br>27<br>27	body weight, lipid profile
**Jenkins et al. 2008**	H	64	C	4	43[27]<br>15F:12M	73g Almonds<br>37g Almonds<br>CRAB	27<br>27<br>27	body weight, lipid profile
**Johnston et al. 2013**	H	41	P	8	64[44]<br>16M:28F	28g Peanuts<br>CARB	22<br>21	body fat, body weight↓, energy intake, FBG, insulin, WC
**Jones et al. 2023**	OW or OB	62	C	8	38[35]<br>7M:28F	Macadamia Nuts<br>Habitual	15<br>13	body weight↓, energy intake, FBG, insulin, lipid profile
**Jung et al. 2017**	OW or OB	52	C	4	90[84]<br>11M:73F	56g Almonds<br>CARB	42<br>42	BMI, body fat, body weight, energy intake, FBG, lipid profile
**Kalgaonkar et al. 2011**	PCOS	33	P	6	31[31]<br>0M:31F	36g Walnuts<br>46g Almonds	17<br>14	BMI, body weight, energy intake, FBG, insulin, lipid profile, nutrients intake
**Katz et al. 2012**	OW or OB	57	C	8	46[40]<br>18M:28F	56g Walnuts<br>Habitual	22<br>18	BMI, body weight, FBG, insulin, lipid profile, WC
**Kendall et al. 2014**	MetS	54	C	NR	20[20]<br>8M:12F	85g Pistachio<br>CARB	20<br>20	GLP-1, ghrelin
**Khorramirad et al. 2021**	T2DM	52	C	12	48[44]	50g Pistachio<br>Habitual	21<br>23	BMI, body weight, energy intake, FBG, lipid profile, WC↑

**Kocyigit et al. 2006**	H	33	P	3	44[44]<br>24M:24F	Pistachio<br>Habitual l	22<br>22	BMI, body weight, energy intake, FBG, lipid profile
**Konieczna et al. 2023**	H	63	P	52	531[305]<br>121M:230	30g MN<br>1L/wk EVOO	102<br>112	BMI, body fat↓, body lean↑, body weight, energy intake, lipid profile
**Lasa et al. 2014**	T2DM	67	P	52	141[141]<br>61M:80F	30g MN<br>1L/wk EVOO	50<br>74	BMI, body weight↓, FBG, GLP-1, insulin
**Le et al. 2016**	OW or OB	50	P	24	245[213]<br>0M:213F	42g Walnuts<br>LOW FAT<br>LOW CARB	82<br>82<br>81	BMI, body weight, lipid profile
**Lee et al. 2014**	MetS	NR	P	6	61[60]<br>0M:60F	30g MN<br>HLS	30<br>30	BMI↓, body weight, FBG, insulin, lipid profile
**Li et al. 2010**	OW or OB	46	P	12	70[62]<br>13M:57F	53g Pistachio<br>CARB	27<br>25	BMI↓, body weight, FBG, insulin, lipid profile
**Li et al. 2011**	T2DM	58	C	4	22[20]<br>9M:11F	56g Almonds<br>Nut-Free	20<br>20	BMI, body fat, body weight, FBG, lipid profile, insulin
**Liu et al. 2017**	H	26	P	16	227[169]<br>77M:92F	56g Preload Almonds<br>56g Snack Almonds<br>CARB	58<br>55<br>56	BMI, body fat↓, body weight, energy intake, lipid profile
**Liu et al. 2018**	H	27	P	20	169[85]<br>45M:40F	56g Almonds<br>CARB	57<br>28	BMI, body fat↓, body weight, energy intake, lipid profile, WC
**Ma et al. 2010**	T2DM	58	C	8	24[21]<br>10M:14F	56g Walnuts<br>Nut-Free	11<br>12	BMI, body weight, FBG, insulin, lipid profile, WC
**McKay et al. 2018**	H	60	C	4	26[26]<br>21M:5F	42.5g<br>Pecan	15<br>11	insulin, lipid profile

**Mercanlıgil et al. 2007**	HC	48	C	8	15[15]<br>15M:0F	40g Hazelnuts<br>LOW FAT	15<br>15	energy intake, FBG, insulin, lipid profile
**Mohan et al. 2018**	T2DM	51	P	12	300[269]<br>145M:124	30g Cashews<br>Diabetic Diet	129<br>140	BMI, body weight, energy intake↑, FBG, insulin, lipid profile, WC
**Moreira et al. 2014**	OW or OB	28	P	4	65[65]<br>65M:0F	56g CVP<br>56g HOP<br>CARB	22<br>21<br>22	insulin↑
**Moreira et al. 2014**	OW or OB	27	P	4	76[65]<br>65M:0F	56g CVP<br>56g HOP<br>CARB	22<br>21<br>22	body fat, energy intake, FBG, insulin, lipid profile
**Morgan et al. 2000**	H	41	P	8	19[19]<br>4M:15F	68g Pecan<br>Nut-Free	10<br>9	body weight, energy intake, lipid profile
**Morgan et al. 2002**	HC	56	C	6	67[42]<br>17M:25F	64g Walnuts<br>LOW FAT	42<br>42	BMI, lipid profile
**Mukuddem et al. 2007**	MetS	45	P	8	64[64]<br>29M:35F	85.5g Walnuts<br>85.5g Cashews<br>Nut-Free	21<br>21<br>22	BMI, body weight, lipid profile
**Ndanuko et al. 2018**	H	44	P	12	620[211]<br>60M:151F	30g Walnuts<br>HLS	82<br>62	body weight↓
**Neale et al. 2017**	OW or OB	43	P	52	251[115]<br>65M:186F	30g Walnuts<br>HLS	23<br>43	body weight↓, energy intake
**Nijssen et al. 2023**	H	65	C	8	35[28]<br>14M:14F	60g MN<br>Nut-Free	13<br>15	BMI, body fat, body weight, WC

**Nijssen et al. 2024**	H	65	C	8	35[28]<br>14M:14F	60g MN<br>Nut-Free	13<br>15	body weight, lipid profile
**Njike et al. 2015**	D	56	C	12	112[97]<br>31M:81F	56g Walnuts<br>Habitual	48<br>49	BMI, body fat↑, FBG, lipid profile, WC↑
**Palacios et al. 2019**	PD	48	C	6	54[33]<br>17M:16F	85g Almonds<br>CARB	15<br>18	energy intake, FBG, insulin, lipid profile
**Petersen et al. 2022**	T2DM	59	P	24	107[107]<br>37M:70F	35g Peanuts<br>LOW FAT	44<br>32	body weight↓, BMI, energy intake, FBG↓, insulin↓
**Pinto et al. 2019**	CVD	64	P	52	106[100]<br>63M:37F	30g MN<br>1L/wk EVOO	36<br>34	BMI, energy intake↓, FBG, lipid profile, WC
**Prater et al. 2024**	T2DM	59	P	24	107[107]<br>37M:70F	35g Peanuts<br>LOW FAT	44<br>32	appetite↓, fullness↑, ghrelin↓, PYY↑
**Rajaram et al. 2010**	H	41	C	4	27[25]<br>11M:14F	34g Almonds<br>68g Almonds<br>LOW FAT	27<br>27<br>27	body weight
**Ravi et al. 2015**	Dyslipidemia	39	P	12	56[42]<br>46M:10F	80g Pistachio<br>HLS	21<br>21	BMI, body weight, FBG, lipid profile, WC
**Razquin et al. 2010**	CVD	68	P	52	541[541]<br>248M:293F	30g MN<br>1L/wk EVOO	239<br>302	BMI, body weight, WC
**Reis et al. 2013**	OW or OB	35	C	NR	15[15]<br>0M:15F	42.5g Peanuts<br>CARB	15<br>15	energy intake, FBG, GLP-1, insulin, nutrient intake, PYY↑
**Reis et al. 2019**	OW or OB	40	P	8	72[54]<br>0M:54F	5g Brazil Nuts<br>Habitual	29<br>25	BMI, body fat, FBG, insulin, lipid profile, WC
**Ren et al. 2020**	T2DM	72	P	12	50[45]	56g Almonds	22	BMI↓, body weight↓, GLP-1

					20M:25F	LOW FAT	23	
**Rocha et al. 2023**	OW	32	P	8	40[29]	45g MN (15 g Brazil nuts + 30 g cashew nuts)	14	fullness, hunger
					NR	Nut-Free	14	
**Rock et al. 2016**	IS	50	P	52	119[119]	42g Walnuts	41	body weight↓, FBG↓, insulin, lipid profile, WC
	IR	50			0M: 119F	Low Fat	39	
					126[126]	CARB	39	
					0M: 126F	42g Walnuts	41	
						Low Fat	43	
						CARB	42	
**Rock et al. 2017**	OW or OB	49	C	NR	28[28]	42.5g Walnuts	28	CCK, ghrelin, glucagon, GIP, GLP-1↓, insulin, PP, PYY
					9M: 19F	Nut-Free	28	
**Rock et al. 2017**	OW or OB	53	P	24	100[97]	35g Walnuts	47	BMI↓, body weight, lipid profile, WC↓
					58M: 42F	Hypoenergetic	50	
**Rock et al. 2020**	OW or OB	56	P	16	100[93]	42g Pistachio	45	BMI, body weight, energy intake↓, FBG, lipid profile, WC
					38M: 62F	Hypoenergetic	48	
**Rodrigues et al. 2019**	H	22	C	NR	34[30]	28g Walnuts	30	appetite↓, fullness↑, FBG, hunger, insulin, lipid profile
					14M: 16F	28g Walnuts	30	
						FAT	30	
**Ros et al. 2004**	HC	55	C	4	21[20]	52.5g Walnuts	21	body weight, lipid profile
					8M: 12F	MeDiet	21	
**Riley et al. 2024**	prediabetes	51	C	12	64[51]	100g Almonds	51	BMI, body weight, energy intake
					34M:17F	Low fat	51	
**Ruisinger et al. 2015**	dyslipidemia	60	P	4	50[48]	57 g Pistachios	22	

					24M:24F	CARB	26	BMI, body weight, energy intake, lipid profile, WC
**Sabaté et al. 2003**	H	41	C	4	27[25]<br>14M: 11F	34g Almonds<br>68g Almonds<br>Low fat	25<br>25<br>25	body weight, FBG, lipid profile
**Salas et al. 2008**	CVD	67	P	12	842[820]<br>394M: 426F	30g MN<br>1L/wk EVOO	411<br>409	body weight, energy intake↑
**Sanchis et al. 2019**	KD	71	C	4	13[13]<br>7M:6F	30g Walnuts<br>Isoenergetic	NR	BMI, FBG, lipid profile
**Sapp et al. 2022**	HFG	42	C	6	51[50]<br>0M: 50F	28g Peanuts<br>CARB	50<br>50	body weight, energy intake, FBG, insulin, lipid profile, energy intake
**Sauder et al. 2015**	T2DM	56	C	4	34[30]<br>15M: 15F	93.5g Pistachio<br>HLS	30<br>30	BMI, body weight↓, FBG↓, insulin, lipid profile
**Sayer et al. 2017**	OW or OB	35	P	12	24[22]<br>3M: 19F	28g Almonds<br>Isoenergetic	NR	appetite, body weight↓, body fat↓
**Schutte et al. 2006**	MetS	46	P	8	68[62]<br>28M: 34F	85.5g Walnuts<br>85.5g Cashew nuts<br>Habitual	20<br>21<br>21	BMI, FBG, lipid profile, WC
**Sheridan et al. 2007**	HC	60	C	4	15[15]<br>11M: 4F	42.5g Pistachio<br>Fat	15<br>15	BMI, body weight, energy intake, FBG, lipid profile
**Souza et al. 2018**	OW or OB	NR	P	8	60[46]<br>0M: 46F	20g Almonds<br>Isoenergetic	24<br>22	BMI↓, body fat↓, body weight, energy intake, FBG, lipid profile, WC↓
**Souza et al. 2019**	OW or OB	NR	P	8	60[46]<br>0M: 46F	20g Almonds<br>Isoenergetic	24<br>22	energy intake

**Spiller et al. 1998**	HL	53	P	4	45[45]<br>12M: 33F	100g Almonds<br>Fat<br>Fat	18<br>15<br>12	body weight, energy intake, lipid profile
**Spiller et al. 2003**	HC	61	P	4	38[38]<br>12M: 26F	100g Almonds<br>100g Almonds<br>Almond butter	14<br>14<br>10	body weight, energy intake, FBG, lipid profile
**Tan et al. 2013**	T2DM	NR	P	4	150[137]<br>48M: 49F	43g Almonds<br>43g Almonds<br>43g Almonds<br>43g Almonds<br>Nut-Free	28<br>28<br>26<br>28<br>27	BMI↓, body fat, body weight↓, energy intake, FBG, insulin, lipid profile, WC↓
**Tapsell et al. 2004**	T2DM	59	P	24	58[57]<br>34M: 24F	30g Walnuts<br>Low Fat<br>Low Fat	17<br>20<br>21	BMI, body fat, body weight, energy intake, lipid profile
**Tapsell et al. 2009**	OW and DM	54	P	52	50[35]<br>NR	30g Walnuts<br>Low Fat	18<br>17	body fat, body weight↓, energy intake↓, FBG↓, insulin↓, lipid profile
**Tapsell et al. 2009**	OW	53	C	NR	24[16]<br>12M: 12F	Walnuts<br>Olive oil	16<br>16	energy intake, REE
**Tey et al. 2012**	OW or OB	42	P	12	110[107]<br>61M: 46F	30g Hazelnuts<br>60g Hazelnuts<br>Nut-Free	33<br>37<br>37	BMI, body fat, body weight, lipid profile
**Tindall et al. 2019**	CVD	43	C	6	45[36]<br>25M: 20F	78g Walnuts<br>FAT	36<br>36	body weight, FBG, insulin, lipid profile

						FAT	36	
**Wang et al. 2012**	MetS	52	P	12	90[90]<br>41M: 49F	42g Pistachio<br>70g Pistachio<br>Habitual	27<br>29<br>30	FBG, insulin, lipid profile
**Wang et al. 2021**	MetS	46	P	12	224[109]<br>68M: 156F	56g Peanuts<br>CARB	109<br>100	BMI, body weight, energy intake, FBG, lipid profile, WC
**Wang et al. 2021**	MetS	46	P	12	224[209]	56g Peanuts<br>CARB	109<br>100	body weight, FBG, WC
**Wein et al. 2003**	OW or OB	27	P	24	65[65]<br>28M: 37F	84g Almonds<br>CARB	32<br>33	BMI↓, body fat↓, body weight↓, FBG, insulin, lipid profile, WC↓
**Wein et al. 2010**	Prediabetes	54	P	16	65[65]<br>17M: 48F	60g Almonds<br>Nut-Free	32<br>33	BMI, body weight, energy intake, FBG, insulin↓, lipid profile, WC
**Wein et al. 2013**	T2DM	62	P	24	60[60]<br>30M: 30F	42g Peanuts<br>Nut-Free	30<br>30	BMI↓, body weight, FBG, lipid profile, WC↓
**Williams et al. 2019**	OW or OB	48	C	3	32[24]<br>9M:15F	Almonds<br>CARB	24<br>24	Body weight, lipid profile
**Wilson et al. 2014**	OW or OB	57	C	6	22[22]<br>6M: 16F	35.4g Pistachio<br>Nut-Free	22<br>22	BMI, FBG, insulin, lipid profile, WC
**Wilson et al. 2022**	H	18	C	NR	50[36]<br>3M:33F	29g Walnuts<br>Isocaloric	38<br>38	appetite↓
**Zambo´n et al. 2000**	HC	56	C	6	55[49]<br>28M: 27F	48.5g Walnuts<br>MeDiet	25<br>24	body weight, lipid profile
**Zaveri et al. 2009**	H	40	P	12	45[36]	28g Almonds	11	

		45M: 0F	CARB	13	BMI, body fat, body weight, energy intake, WC
			Nut-Free	12	

OW or OB: Overweight or obese; H: Healthy; T2DM: Type 2 diabetes mellitus; HC: Hypercholesterolemia; CVD: Cardiovascular disease; HL: Hyperlipidemic; MetS: Metabolic syndrome; HFG: High fasting glucose; KD: Kidney disease; IR: Insulin resistance; IS: Insulin sensitivity;; PCOS: Polycystic ovary syndrome; GLP-1: Glucagon-like peptide-1; PP: Pancreatic polypeptide; GIP: Glucose-dependent insulinotropic peptide; PYY: Peptide YY; CCK; Cholesystokinin.

Table S4. Summary of articles comparing two different types of nuts

Trial	Heath Status	Mean Age	Design	Duration (weeks)	N	Group Details	Group size, no	Reported Outcome
Hou et al. 2018	T2DM	67	P	12	32[25]<br>15M:10F	60g Peanuts<br>55g Almonds	11<br>14	BMI , lipid profile, WC
Kalgaonkar et al. 2011	PCOS	33.2	P	6	31[31]<br>0M:31F	36g Walnuts<br>46g Almonds	17<br>14	BMI, body weight, energy intake, lipid profile, nutrients intake
Mukuddem et al. 2007	MetS	45	P	8	64[64]<br>29M:35F	85.5g Walnuts<br>85.5g Cashews<br>Control	21<br>21<br>22	BMI, body weight, lipid profile
Schutte et al. 2006	MetS	45.5	P	8	68[62]<br>28M:34F	85.5g Walnuts<br>85.5g Cashew<br>Control	20<br>21<br>21	BMI, FBG, lipid profile, WC

Table S5. Summary of articles reporting changes in appetite, appetite-regulating hormones, and energy metabolism in response to nut interventions

	**Appetite**		**Appetite Regulating Hormones**				**Energy Metabolism**	
	Hunger	Fullness	CCK	Ghrelin	GLP-1	PYY	DIT	REE
# of articles reporting changes/ # of total reporting articles	↓ **2/7**	↑ **2/7**	↑ **1/5**	↑ **2/6**	↓ **1/6**	↑ **2/6**	↑ **2/3**	**0/3**

Table S6. GRADE certainty of evidence for effects of nut consumption on anthropometric measures, body composition, energy intake, and blood lipids. Randomized evidence started at high certainty and was downgraded for serious or very serious concerns. Ratings are provisional pending confirmation of clinically important decision thresholds, completion of missing risk-of-bias assessments, and resolution of dependent comparisons from the same randomized trial.

Outcome	k	Mean difference (95% CI)	Risk of bias	Inconsistency	Indirectness	Imprecision	Publication bias	Certainty	Rationale
**Body mass**	114	0.03 kg (-0.25 to 0.31)	Not serious*	Serious	Not serious†	Serious‡	Not suspected	**Low**	I^2 = 65.3%; CI and PI cross the null.
**BMI**	80	-0.02 kg/m² (-0.10 to 0.06)	Not serious*	Serious	Not serious†	Serious‡	Not suspected	**Low**	I^2 = 63.5%; CI and PI cross the null.
**Waist circumference**	63	-0.24 cm (-0.95 to 0.47)	Not serious*	Very serious	Not serious†	Serious‡	Not suspected	**Very low**	I^2 = 92.5%; PI -4.95 to 4.47 cm.
**Energy intake**	66	120 kcal/day (35 to 205)	Not serious*	Very serious	Not serious†	Serious‡	Suspected§	**Very low**	I^2 = 89.9%; PI crosses the null; Egger p = 0.046.
**Percentage fat mass**	37	-0.29 points	Not serious*	Not serious	Not serious†	Serious‡	Not suspected	**Moderate**	I^2 = 6.7%; PI crosses the null.

		(-0.54 to -0.04)							
**Fat mass**	29	-0.41 kg (-0.57 to -0.26)	Not serious*	Not serious	Not serious†	Serious‡	Not suspected	**Moderate**	I² = 0%; PI -0.75 to -0.08 kg.
**Percentage lean mass**	8	0.71 points (0.32 to 1.10)	Not serious*	Not serious	Not serious†	Serious‡	Unclear¶	**Moderate**	k = 8; relative composition measure only.
**Lean mass**	23	0.25 kg (-0.07 to 0.57)	Not serious*	Serious	Not serious†	Serious‡	Suspected§	**Very low**	I² = 80.2%; CI/PI cross the null; Egger p = 0.006.
**Total cholesterol**	97	-0.15 mmol/L (-0.27 to -0.03)	Not serious*	Serious	Not serious†	Serious‡	Not suspected	**Low**	I² = 87.5%; PI crosses the null.
**LDL-C**	102	-0.17 mmol/L (-0.24 to -0.10)	Not serious*	Serious	Not serious†	Threshold needed‡	Suspected§	**Low**	I² = 85.8%; PI crosses the null; Egger p = 0.001.
**HDL-C**	99	0.02 mmol/L (-0.02 to 0.07)	Not serious*	Very serious	Not serious†	Serious‡	Not suspected	**Very low**	I² = 99.1%; CI/PI cross the null.

**Triglycerides**	103	-0.57 mmol/L (-1.04 to -0.10)	Not serious*	Very serious	Not serious†	Serious‡	Suspected§	**Very low**	I² = 99.2%; PI crosses the null; Egger p = 0.039.

Note. CI, confidence interval; GRADE, Grading of Recommendations Assessment, Development and Evaluation; PI, prediction interval.

k denotes intervention comparisons/effect estimates, not necessarily unique randomized trials. Unique participant totals are not shown because crossover designs, shared controls, and multiple publications require study-level de-duplication.

* Most contributing publications were judged at low risk of bias, but several had some concerns or high risk, and updated studies remain unassessed. Confirm using outcome-specific sensitivity analyses excluding high-risk studies.

† No downgrade for indirectness assumes that the review intentionally addresses broad adult populations. Reassess if the target population is narrower.

‡ Final imprecision judgments require prespecified outcome-specific thresholds for clinically important benefit and harm.

§ Significant Egger regression was interpreted as possible small-study effects, not proof of publication bias. Reassess after accounting for dependent comparisons from the same trial.

¶ Funnel-plot asymmetry was not assessed for percentage lean mass because

Figure S1. Random-effects meta-analysis of the weighted mean difference (WMD) in body weight (kg) following nut supplementation, with subgroup analysis by nut type.

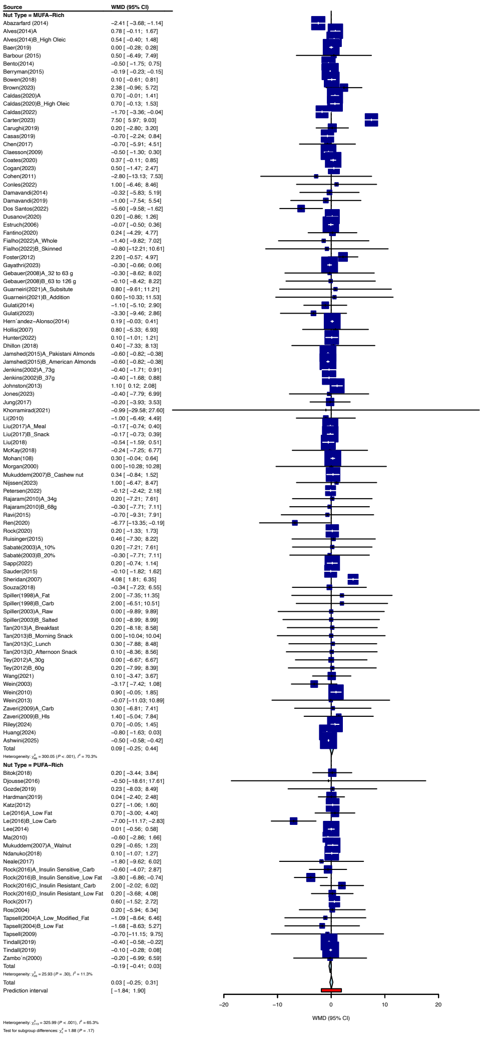

Figure S2. Random-effects meta-analysis of the weighted mean difference (WMD) in body weight (kg) following nut supplementation, with subgroup analysis by control group diets.

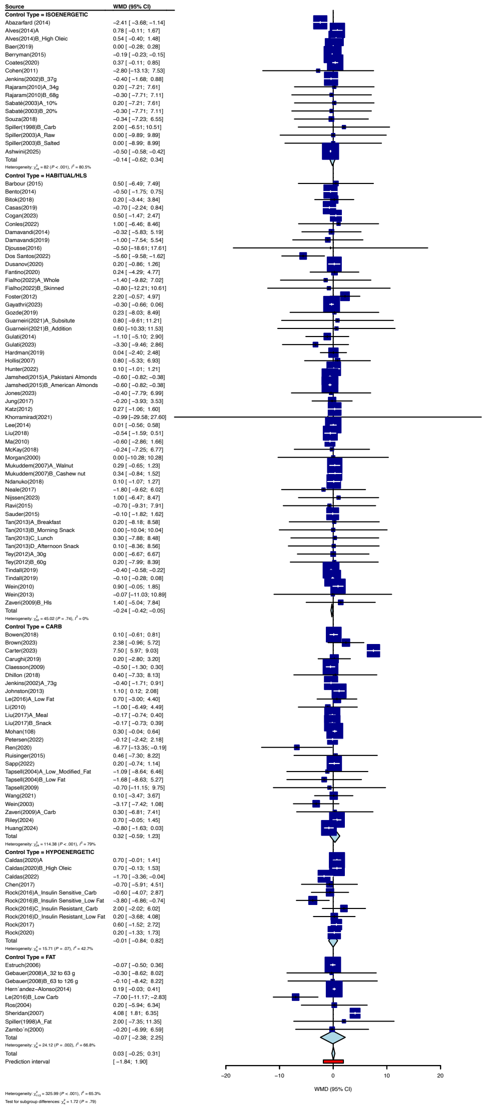

Figure S3. Random-effects meta-analysis of the weighted mean difference (WMD) in BMI (kg/m^2) following nut supplementation, with subgroup analysis by nut type.

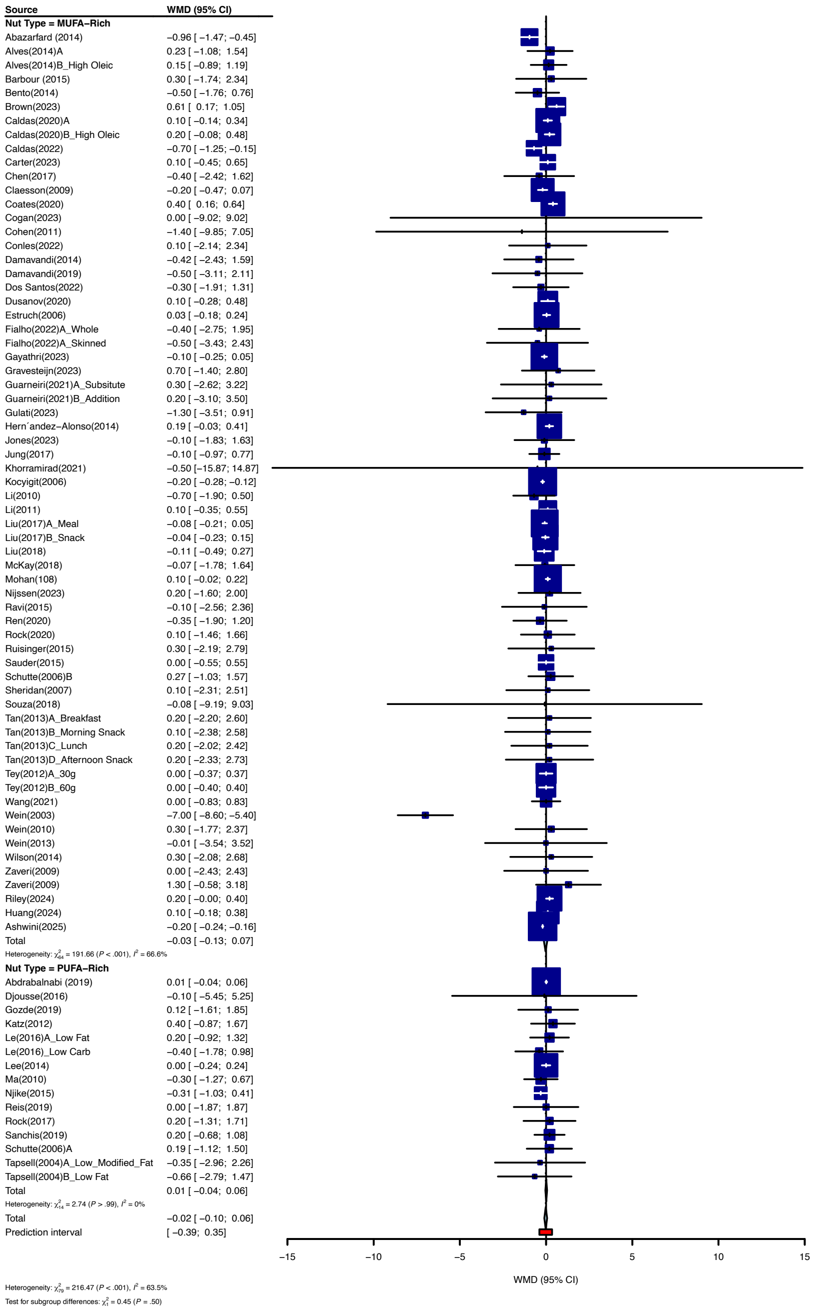

Figure S4. Random-effects meta-analysis of the weighted mean difference (WMD) in BMI (kg/m^2) following nut supplementation, with subgroup analysis by control group diets.

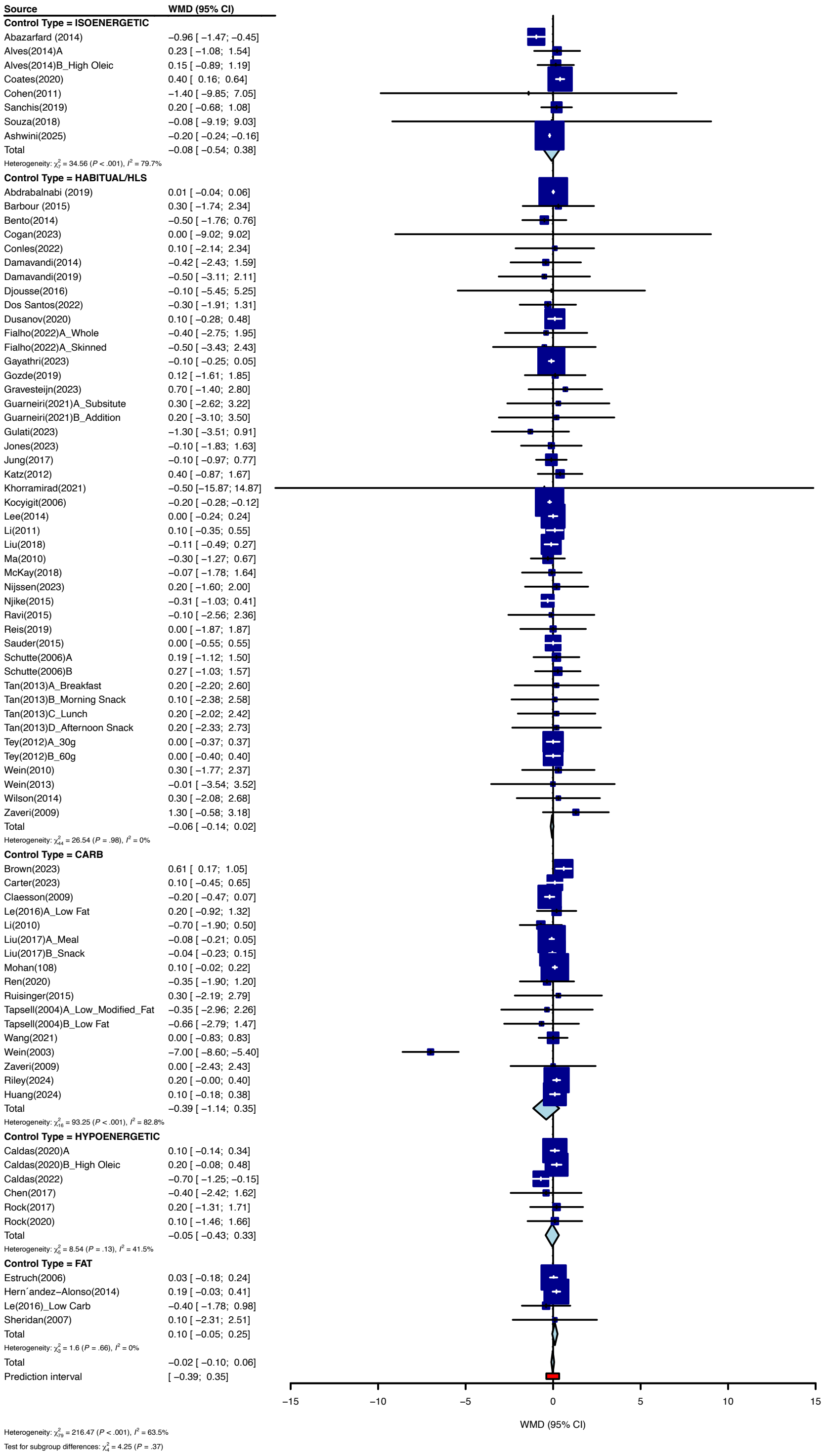

Figure S5. Random-effects meta-analysis of the weighted mean difference (WMD) in WC (cm) following nut supplementation, with subgroup analysis by nut type.

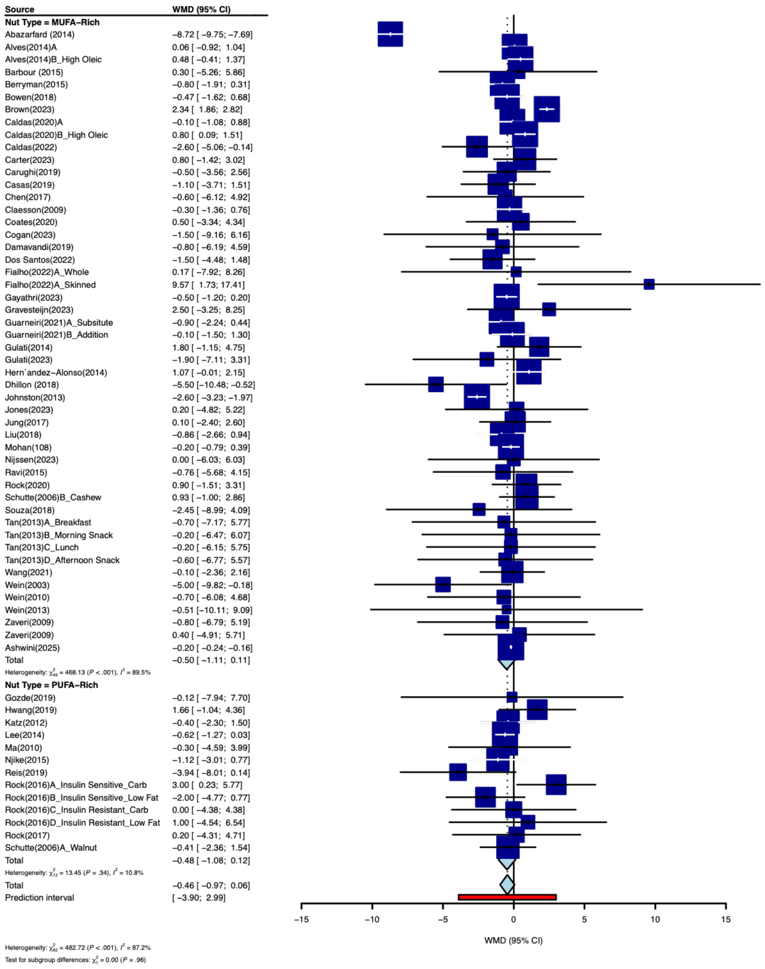

Figure S6. Random-effects meta-analysis of the weighted mean difference (WMD) in WC (cm) following nut supplementation, with subgroup analysis by control group diets.

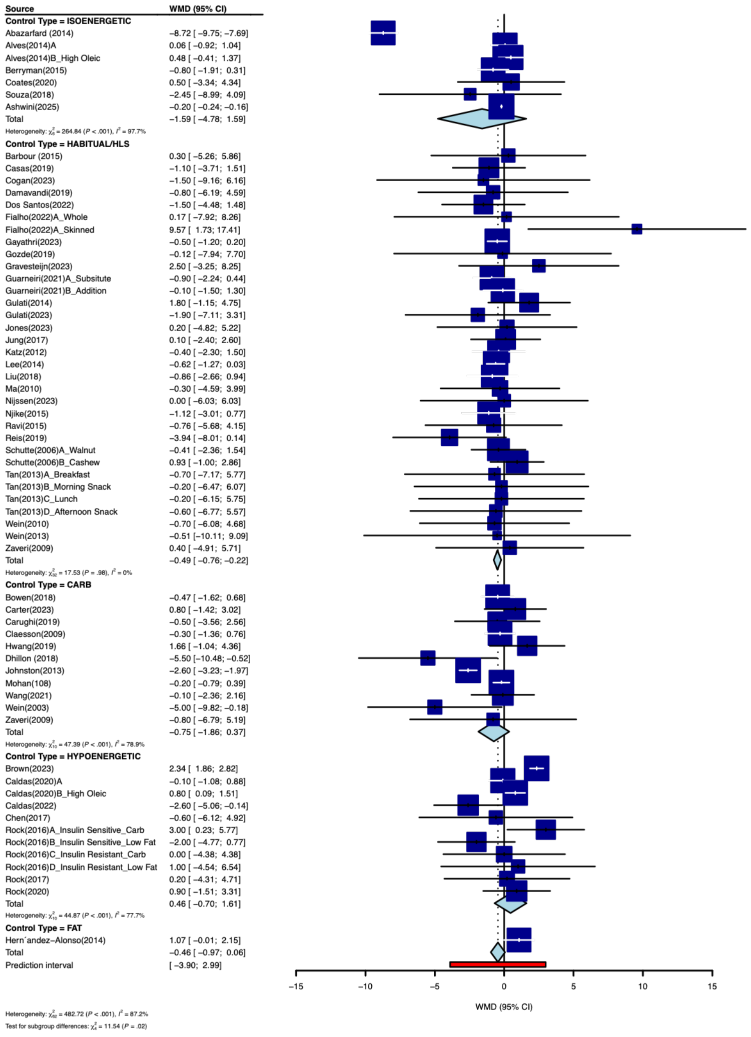

Figure S7. Random-effects meta-analysis of the weighted mean difference (WMD) in total energy intake per day (kcal/d) following nut supplementation, with subgroup analysis by nut type.

Source	WMD (95% CI)
**Nut Type = MUFA–Rich**	
Alper(2002)A_ADD	45.41 [−88.43; 179.25]
Alper(2002)B_SUB	−167.30 [−286.24; −48.37]
Barbour (2015)	223.47 [−7.62; 454.56]
Bento(2014)	−30.00 [−354.01; 294.01]
Bowen(2018)	−71.94 [−310.41; 166.53]
Brown(2023)	283.94 [252.68; 315.20]
Caldas(2020)A	15.30 [−619.50; 650.10]
Caldas(2020)B_High Oleic	79.00 [−421.21; 579.21]
Caldas(2022)	−85.80 [−542.08; 370.48]
Carter(2023)	−72.66 [−229.34; 84.03]
Carughi(2019)	−192.16 [−383.30; −1.02]
Casas(2019)	184.00 [−46.94; 414.94]
Claesson(2009)	98.19 [−301.66; 498.04]
Coates(2020)	167.07 [−263.65; 597.78]
Cogan(2023)	−140.00 [−622.40; 342.40]
Cohen(2011)	98.19 [−807.30; 1003.68]
Damavandi(2014)	1049.19 [739.62; 1358.76]
Damavandi(2019)	−142.90 [−352.52; 66.72]
Dhillon(2016)	59.00 [−227.91; 345.91]
Dikariyanto(2020)	−79.50 [−250.88; 91.88]
Dusanov(2020)	13.00 [−213.53; 239.53]
Estruch(2006)	146.00 [8.19; 283.81]
Gayathri(2023)	86.00 [9.11; 162.89]
Guarneiri(2021)A_Subsitute	252.00 [−107.79; 611.79]
Guarneiri(2021)B_Addition	44.00 [−239.42; 327.42]
Hunter(2022)	17.93 [−198.95; 234.80]
Dhillon (2018)	38.50 [−226.97; 303.97]
Johnston(2013)	146.00 [−270.89; 562.89]
Jones(2023)	−79.00
Jung(2017)	84.90 [−67.65; 237.45]
Khorramirad(2021)	−248.00 [−1893.75; 1397.75]
Liu(2017)A_Meal	−90.10 [−217.43; 37.23]
Liu(2017)B_Snack	−179.10 [−321.51; −36.69]
Liu(2018)	−171.71 [−366.94; 23.52]
Mercanligil(2007)	251.00 [−72.56; 574.56]
Moreira Alves(2014)A	276.00 [153.16; 398.84]
Moreira Alves(2014)B_High Oleic	340.00 [237.08; 442.92]
Morgan(2000)	387.00 [5.92; 768.08]
Palacios(2019)	−26.00 [−376.79; 324.79]
Petersen(2022)	516.49 [389.81; 643.17]
Rock(2020)	114.00 [−100.91; 328.91]
Ruisinger(2015)	351.00 [61.68; 640.32]
Sapp(2022)	−52.00 [−380.87; 276.87]
Sheridan(2007)	84.00 [−324.77; 492.77]
Souza(2018)	0.29 [−175.12; 175.70]
Spiller(1998)A_Fat	−135.00 [−372.21; 102.21]
Spiller(1998)B_Carb	−30.00 [−249.53; 189.53]
Spiller(2003)A_Raw	37.00 [−583.64; 657.64]
Spiller(2003)A_Roasted	116.00 [−429.79; 661.79]
Tan(2013)A_Breakfast	114.00 [−232.70; 460.70]
Tan(2013)B_Morning Snack	2164.00 [1643.52; 2684.48]
Tan(2013)C_Lunch	217.00 [−138.94; 572.94]
Tan(2013)D_Afternoon Snack	101.00 [−247.26; 449.26]
Wang(2021)	−24.14 [−137.75; 89.47]
Wein(2010)	−1.00 [−203.85; 201.85]
Zaveri(2009)	−167.40 [−578.95; 244.15]
Zaveri(2009)	−54.30 [−442.40; 333.80]
Riley(2024)	75.60 [−247.19; 398.39]
Huang(2024)	278.00 [225.92; 330.08]
Total	101.34 [30.61; 172.08]
Heterogeneity: $\chi^2_{57} = 380.08$ ($P < .001$), $I^2 = 85\%$	
**Nut Type = PUFA–Rich**	
Djousse(2016)	75.00 [−795.95; 945.95]
Gozde(2019)	−9.00 [−600.85; 582.85]
Lee(2014)	−72.90 [−252.42; 106.62]
Neale(2017)	227.17 [−87.04; 541.39]
Tapsell(2004)A_Low_Modified_Fat	94.68 [−248.88; 438.24]
Tapsell(2004)B_Low Fat	−110.28 [−403.66; 183.10]
Tapsell(2009)	−254.00 [−677.13; 169.13]
Cofa..n(2024)	1531.55 [1363.62; 1699.48]
Total	204.74 [−222.97; 632.45]
Heterogeneity: $\chi^2_{7} = 224.88$ ($P < .001$), $I^2 = 96.9\%$	
Total	120.07 [35.15; 205.00]
Prediction interval	[−509.82; 749.97]

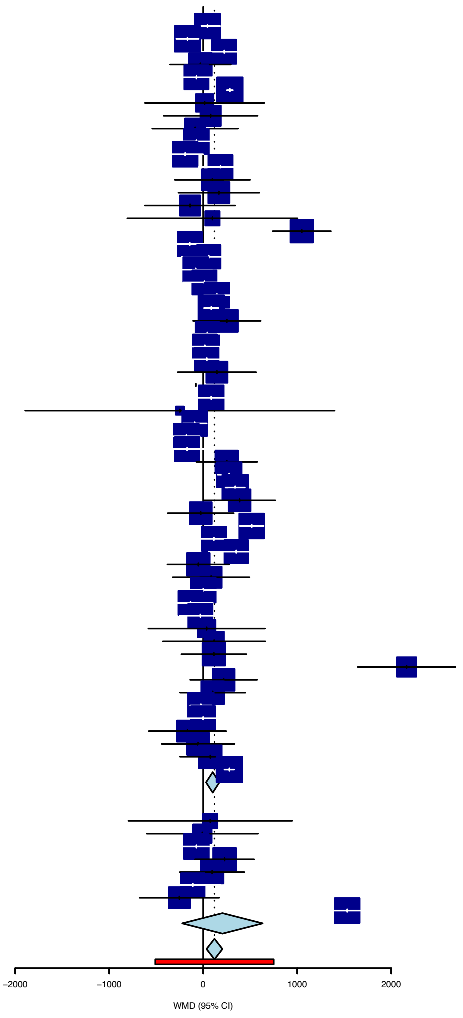

Heterogeneity: $\chi^2_{65} = 643.43$ ($P < .001$), $I^2 = 89.9\%$

Test for subgroup differences: $\chi^2_{1} = 0.22$ ($P = .64$)

Figure S8. Random-effects meta-analysis of the weighted mean difference (WMD) in total energy intake per day (kcal/d) following nut supplementation, with subgroup analysis by control group diets.

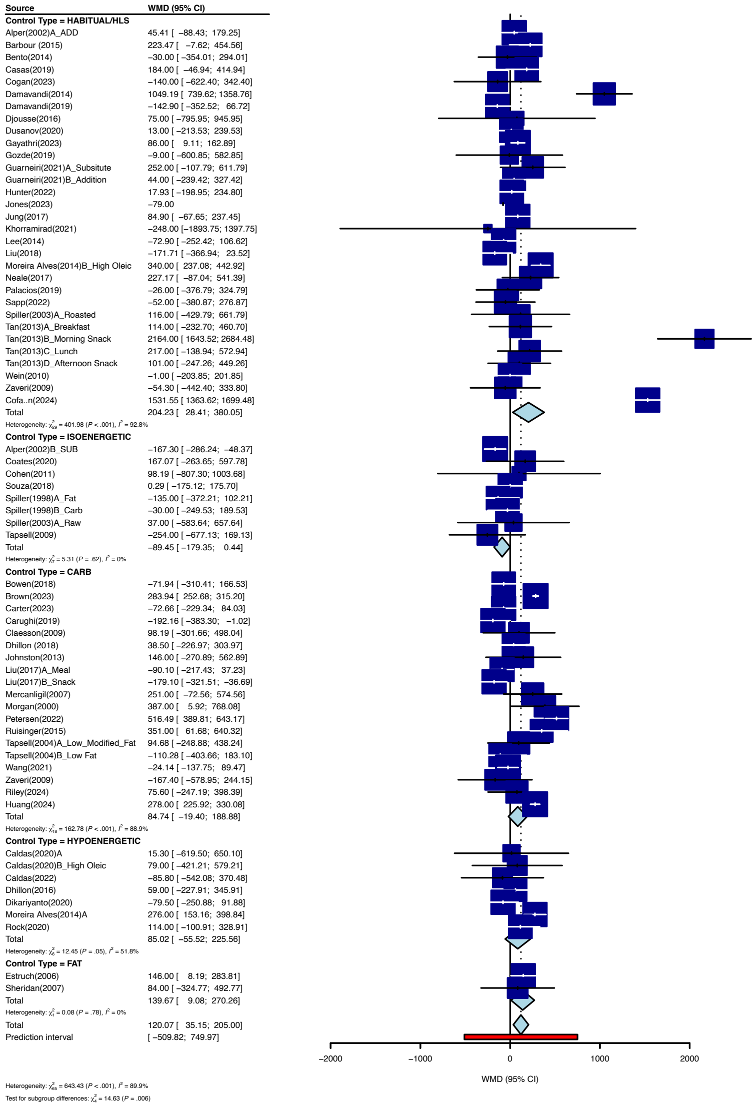

Figure S9. Random-effects meta-analysis of the weighted mean difference (WMD) in body fat percentage (%) following nut supplementation, with subgroup analysis by nut type.

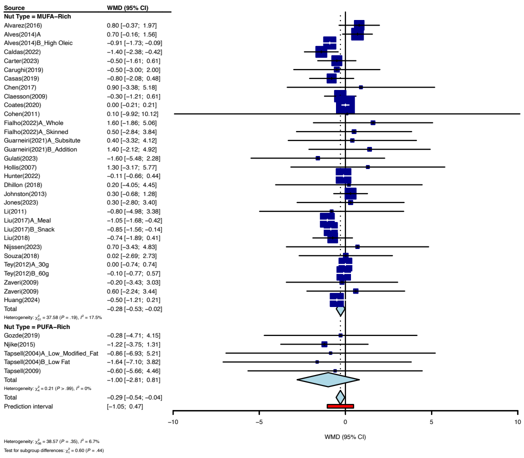

Figure S10. Random-effects meta-analysis of the weighted mean difference (WMD) in body fat percentage (%) following nut supplementation, with subgroup analysis by control group diets.

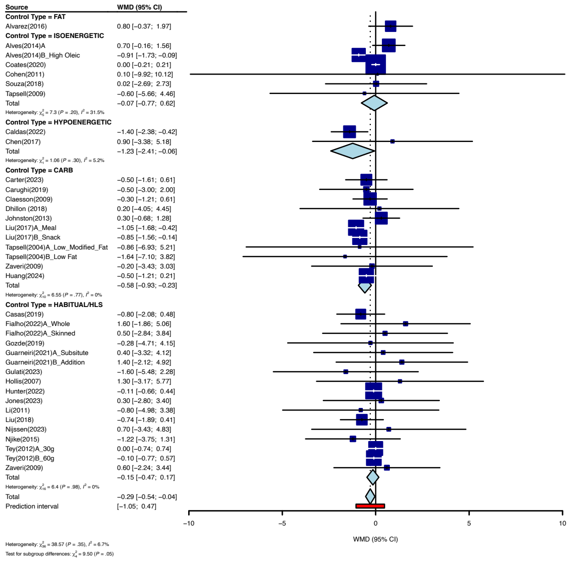

Figure S11. Random-effects meta-analysis of the weighted mean difference (WMD) in body fat mass (kg) following nut supplementation, with subgroup analysis by nut type.

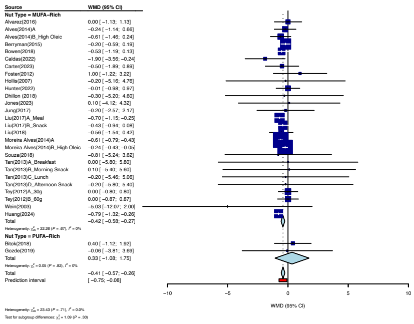

Figure S12. Random-effects meta-analysis of the weighted mean difference (WMD) in body fat mass (kg) following nut supplementation, with subgroup analysis by control group diets.

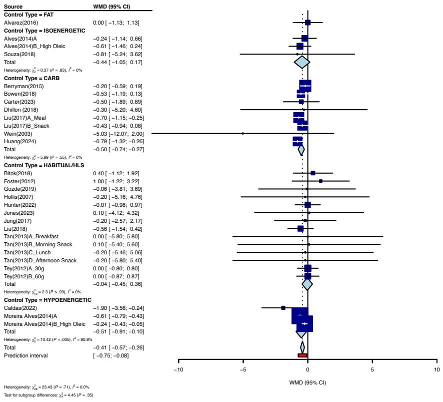

Figure S13. Random-effects meta-analysis of the weighted mean difference (WMD) in body lean percentage (%) following nut supplementation, with subgroup analysis by control group diets.

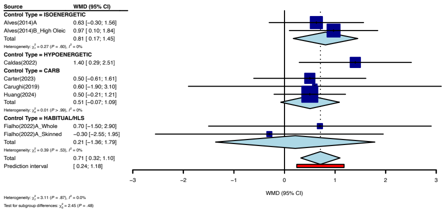

Figure S14. Random-effects meta-analysis of the weighted mean difference (WMD) in body lean mass (kg) following nut supplementation, with subgroup analysis by nut type.

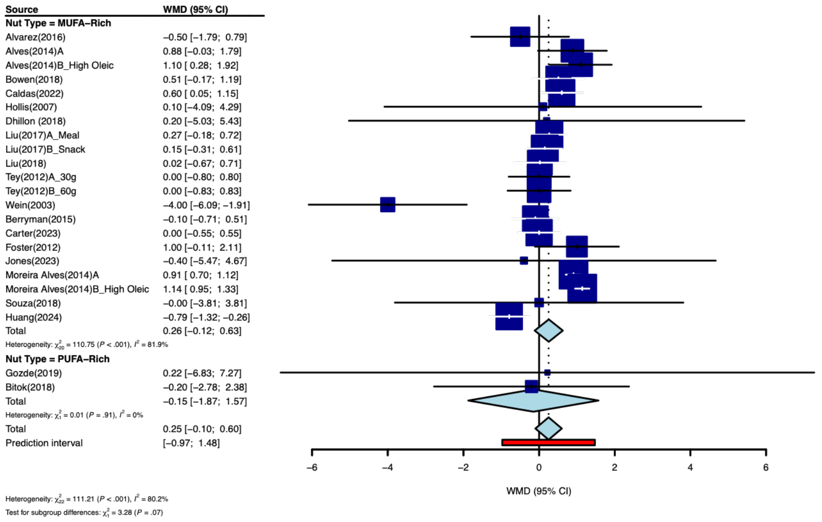

Figure S15. Random-effects meta-analysis of the weighted mean difference (WMD) in body lean mass (kg) following nut supplementation, with subgroup analysis by control group diets.

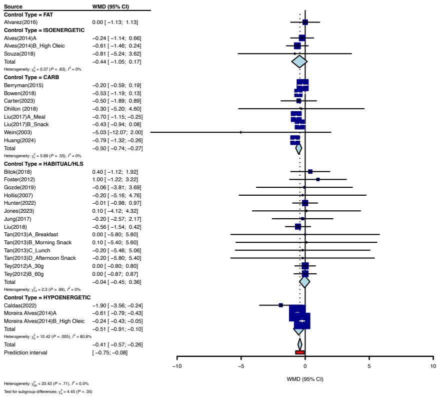

Figure S16. Random-effects meta-analysis of the weighted mean difference (WMD) in TC (mmol/L) following nut supplementation, with subgroup analysis by nut type.

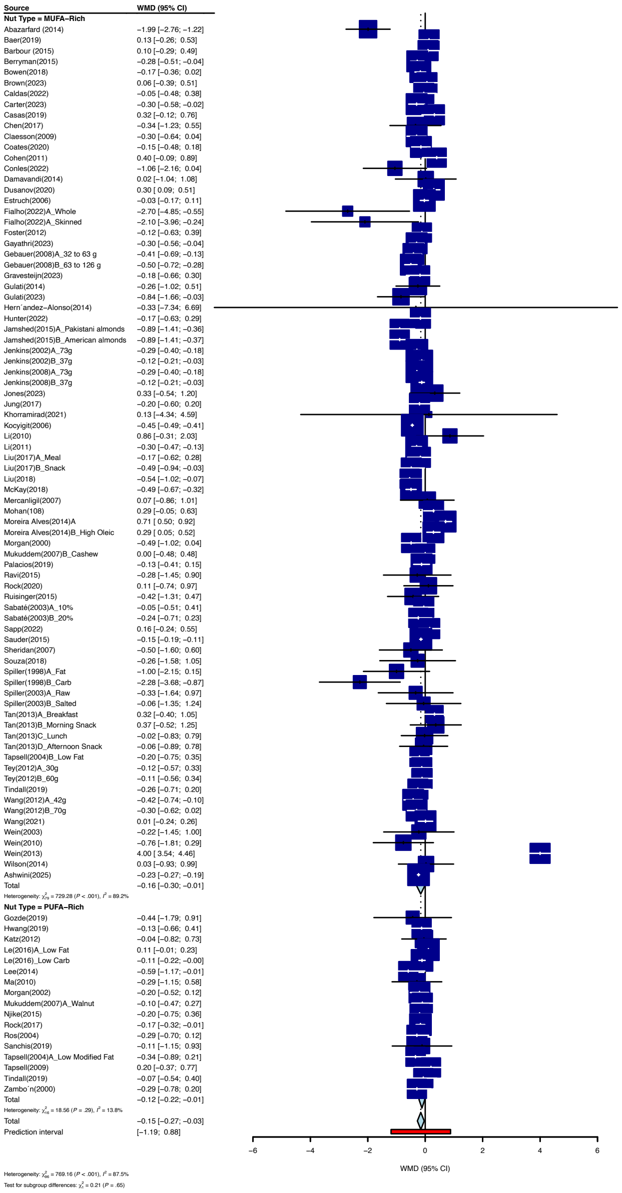

Figure S17. Random-effects meta-analysis of the weighted mean difference (WMD) in TC (mmol/L) following nut supplementation, with subgroup analysis by control group diets.

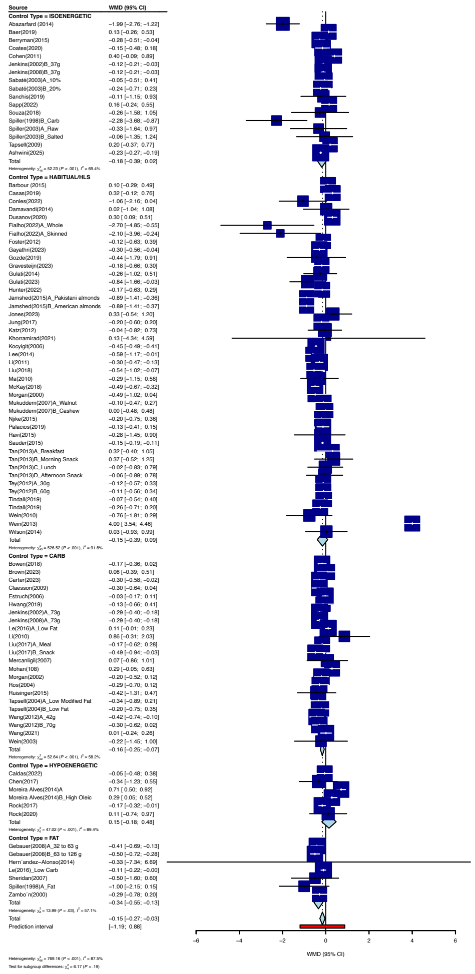

Figure S18. Random-effects meta-analysis of the weighted mean difference (WMD) in LDL-C (mmol/L) following nut supplementation, with subgroup analysis by nut type.

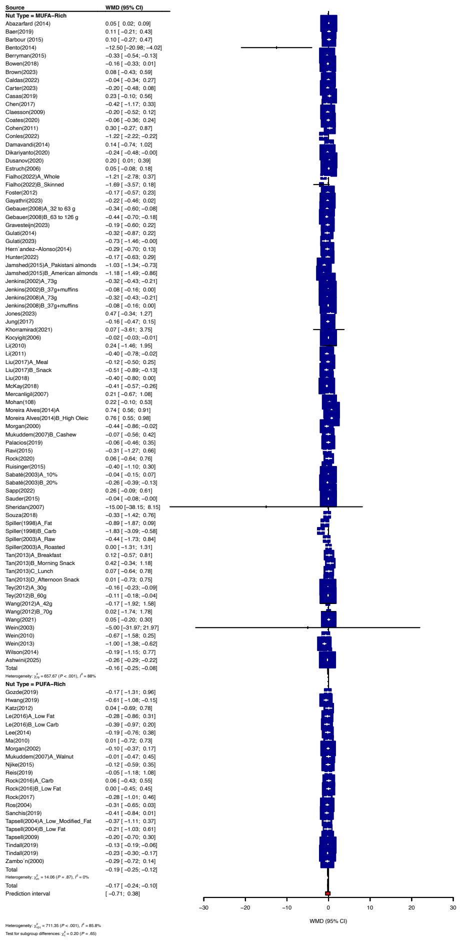

Figure S19. Random-effects meta-analysis of the weighted mean difference (WMD) in LDL-C (mmol/L) following nut supplementation, with subgroup analysis by control group diets.

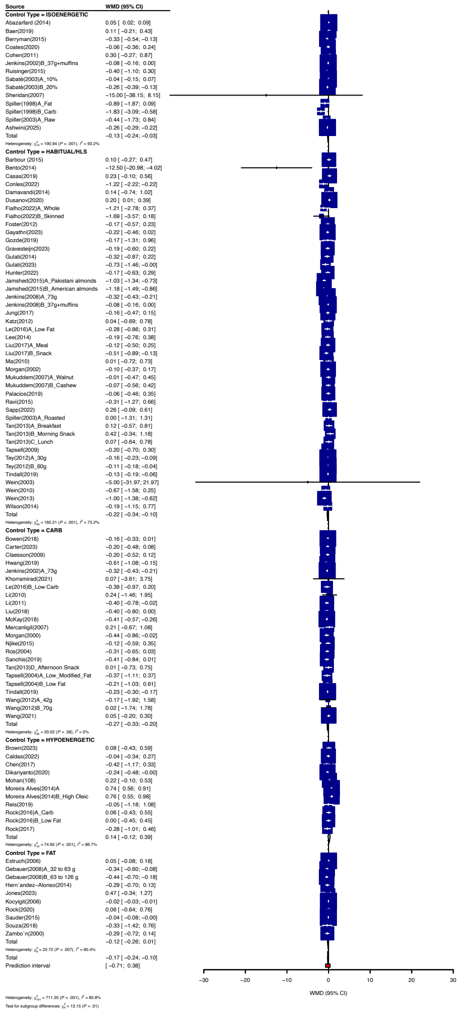

Figure S20. Random-effects meta-analysis of the weighted mean difference (WMD) in HDL-C (mmol/L) following nut supplementation, with subgroup analysis by nut type.

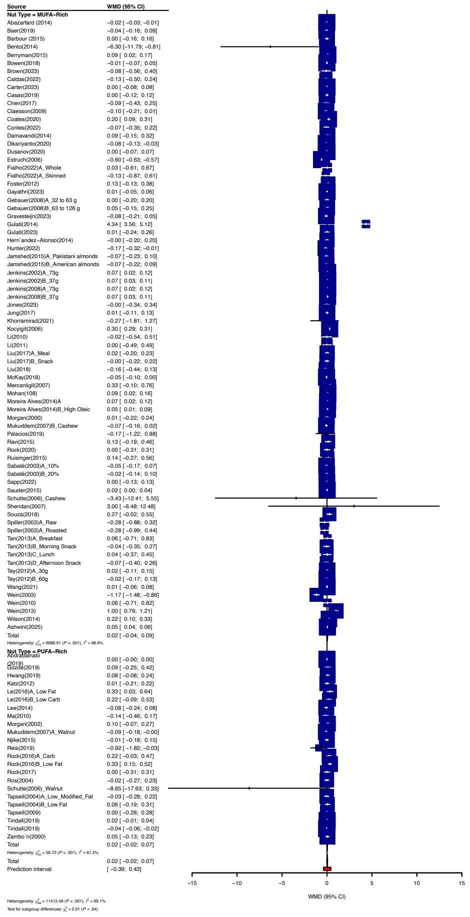

Figure S21. Random-effects meta-analysis of the weighted mean difference (WMD) in HDL-C (mmol/L) following nut supplementation, with subgroup analysis by control group diets.

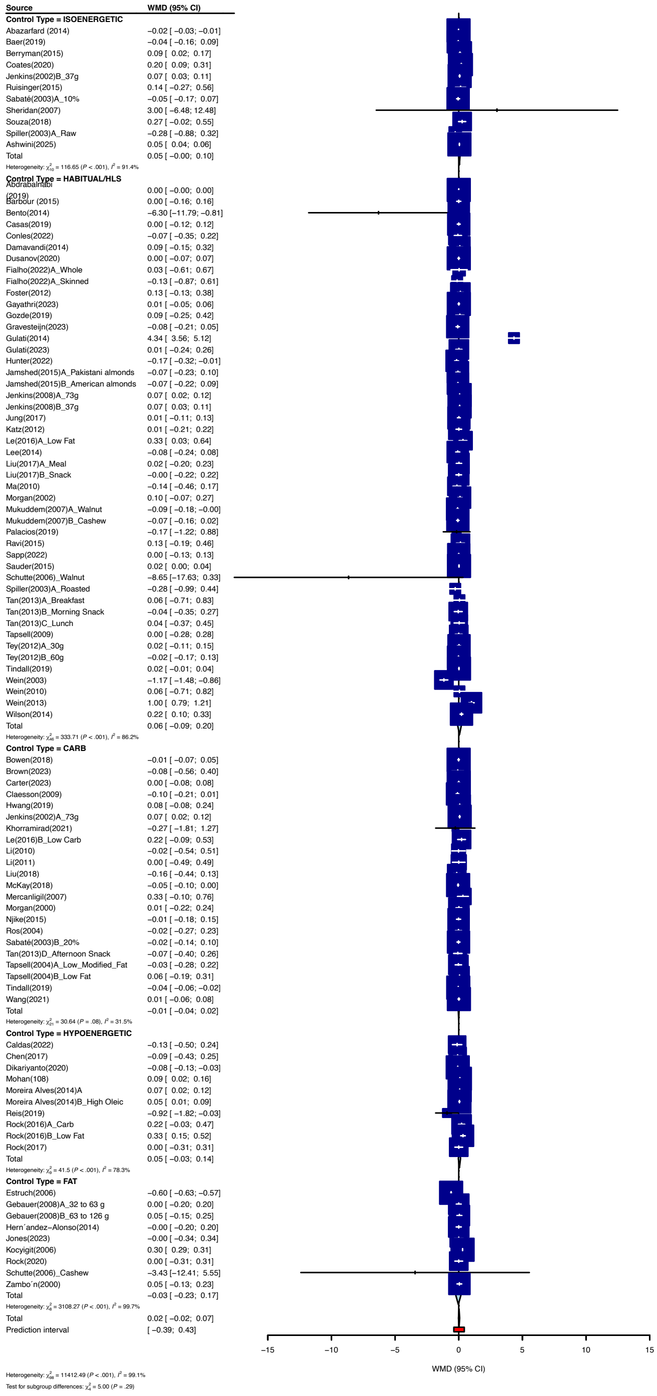

Figure S22. Random-effects meta-analysis of the weighted mean difference (WMD) in TG (mmol/L) following nut supplementation, with subgroup analysis by nut type.

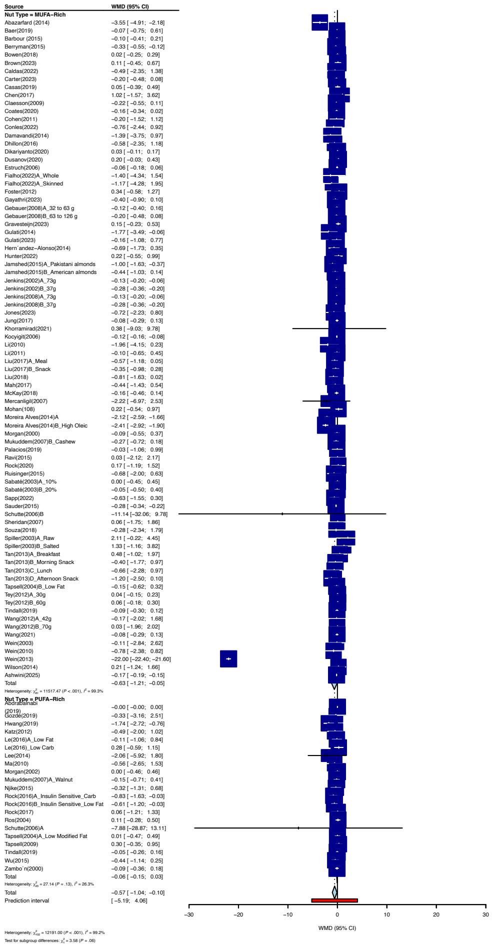

Figure S23. Random-effects meta-analysis of the weighted mean difference (WMD) in TG (mmol/L) following nut supplementation, with subgroup analysis by control group diets.

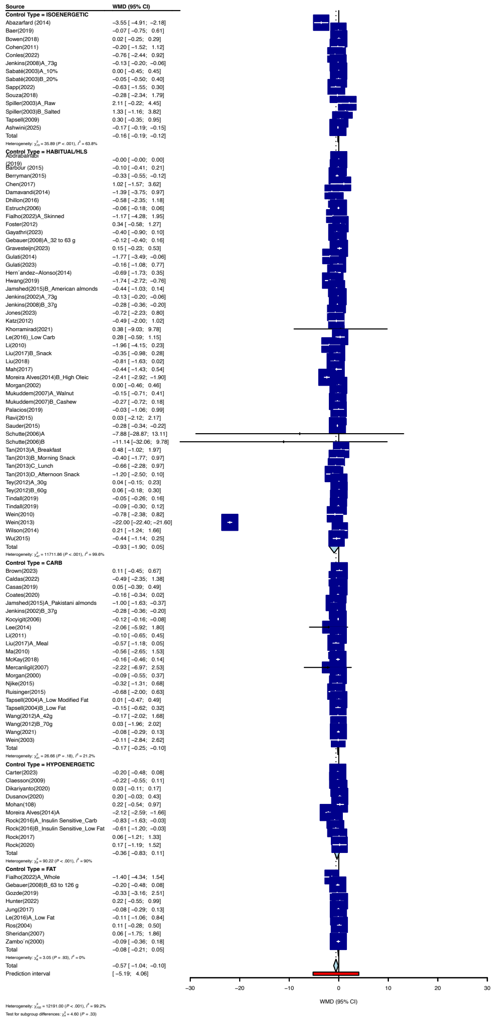

Figure S24. Risk of bias proportion graph (as percentage) for all included A) parallel, B) crossover, included randomized controlled trials.

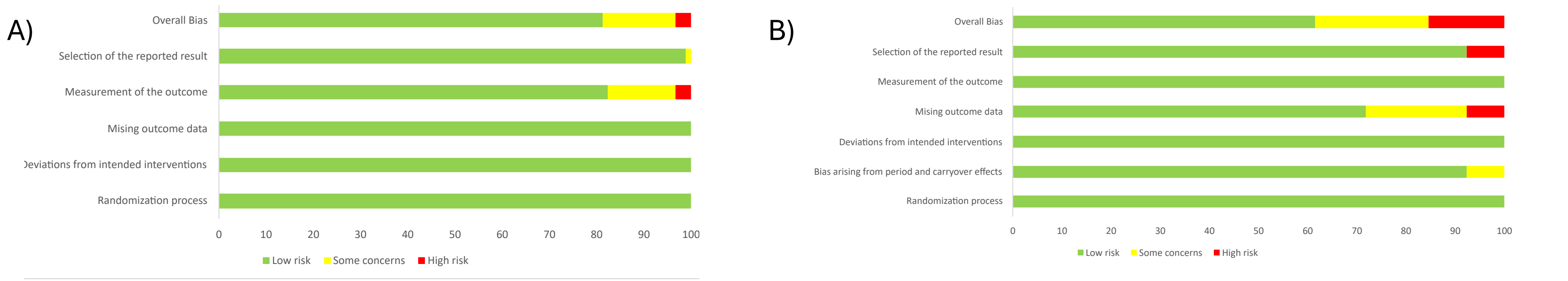

Figure S25. Funnel plots for outcomes with at least 10 contributing intervention comparisons. Panels show (A) body mass, (B) body mass index, (C) waist circumference, (D) energy intake, (E) percentage fat mass, (F) fat mass, (G) lean mass, (H) total cholesterol, (I) LDL-C, (J) HDL-C, and (K) triglycerides. Each point represents one intervention comparison. The vertical dashed line indicates the pooled random-effects mean difference, and the diagonal dashed lines indicate approximate 95% pseudo-confidence limits. Shaded regions correspond to two-sided significance contours. Egger regression suggested possible small-study effects for energy intake ($p = 0.046$), lean mass ($p = 0.006$), LDL-C ($p = 0.001$), and triglycerides ($p = 0.039$); no evidence of asymmetry was detected for the other assessed outcomes. Percentage lean mass was not assessed because fewer than 10 comparisons contributed ($k = 8$). Funnel-plot asymmetry may have causes other than publication bias and should therefore be interpreted cautiously.

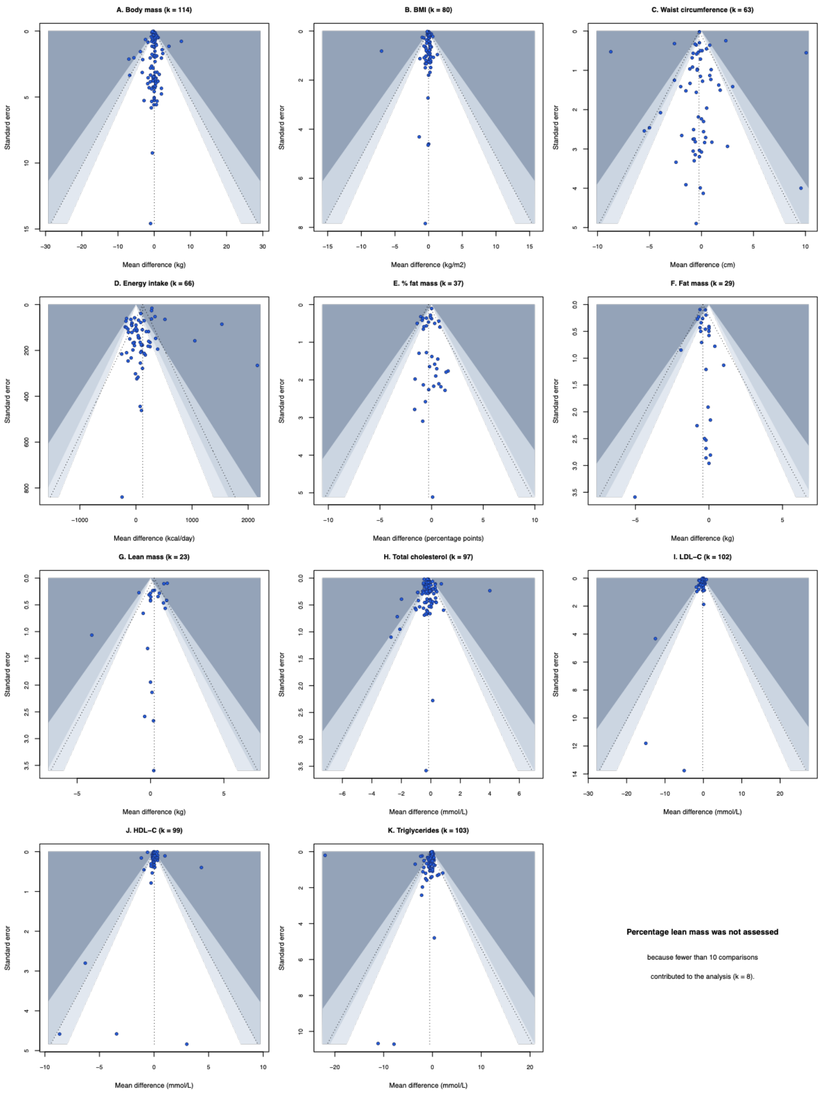

Supplement: Supplementary file 1 [file foods-15-03594-s001.zip › foods-4543973-supplementary.pdf]
